# Discovery of the Active Compounds of the Ethyl Acetate Extract Site of *Ardisia japonica* (Thunb.) Blume for the Treatment of Acute Lung Injury

**DOI:** 10.3390/molecules29040770

**Published:** 2024-02-07

**Authors:** Shuding Sun, Xuefang Liu, Di Zhao, Lishi Zheng, Xiaoxiao Han, Yange Tian, Suxiang Feng

**Affiliations:** 1 Academy of Chinese Medical Sciences, Henan University of Chinese Medicine, Zhengzhou 450003, China; sunshuding@126.com (S.S.); liuxf0213@163.com (X.L.); zhaodiabcd@sina.com (D.Z.); zhenglishia129@126.com (L.Z.); 2 Collaborative Innovation Center for Chinese Medicine and Respiratory Diseases Co-Constructed by Henan Province & Education Ministry of China, Zhengzhou 450046, China; 3 College of Pharmacy, Zhejiang Chinese Medical University, Hangzhou 310053, China; hanxx320@163.com

**Keywords:** Ardisiae Japonicae Herba, anti-acute lung injury, UPLC–Orbitrap Fusion–MS, active compounds

## Abstract

The objective of this study was to identify and evaluate the pharmacodynamic constituents of Ardisiae Japonicae Herba (AJH) for the treatment of acute lung injury (ALI). To fully analyze the chemical contents of various extraction solvents (petroleum ether site (PE), ethyl acetate site (EA), *n*-butanol site (NB), and water site (WS)) of AJH, the UPLC–Orbitrap Fusion–MS technique was employed. Subsequently, the anti-inflammatory properties of the four extracted components of AJH were assessed using the lipopolysaccharide (LPS)-induced MH-S cellular inflammation model. The parts that exhibited anti-inflammatory activity were identified. Additionally, a technique was developed to measure the levels of specific chemical constituents in the anti-inflammatory components of AJH. The correlation between the “anti-inflammatory activity” and the constituents was analyzed, enabling the identification of a group of pharmacodynamic components with anti-inflammatory properties. ALI model rats were created using the tracheal drip LPS technique. The pharmacodynamic indices were evaluated for the anti-inflammatory active portions of AJH. The research revealed that the PE, EA, NB, and WS extracts of AJH included 215, 289, 128, and 69 unique chemical components, respectively. Additionally, 528 chemical components were discovered after removing duplicate values from the data. The EA exhibited significant anti-inflammatory activity in the cellular assay. A further analysis was conducted to determine the correlation between anti-inflammatory activity and components. Seventeen components, such as caryophyllene oxide, bergenin, and gallic acid, were identified as potential pharmacodynamic components with anti-inflammatory activity. The pharmacodynamic findings demonstrated that the intermediate and high doses of the EA extract from AJH exhibited a more pronounced effect in enhancing lung function, blood counts, and lung histology in a way that depended on the dosage. To summarize, when considering the findings from the previous study on the chemical properties of AJH, it was determined that the EA contained a group of 13 constituents that primarily contributed to its pharmacodynamic effects against ALI. The constituents include bergenin, quercetin, epigallocatechingallate, and others.

## 1. Introduction

Acute lung injury (ALI) is a condition triggered by a variety of insults including sepsis, viral or bacterial pneumonia, and mechanical ventilator-induced trauma that frequently progresses to hypoxemic respiratory failure and clinical acute respiratory distress syndrome (ARDS), with a high mortality rate [1,2,3,4]. The primary symptoms include a heightened permeability of the pulmonary blood vessels, an exudation of tissue fluid, a buildup of inflammatory cells, and impaired gas exchange [5,6]. These manifestations pose a significant threat to human health [7]. Presently, there remains a deficiency in the availability of efficacious medications for the management of ALI [8]. Traditional Chinese Medicine (TCM) possesses distinct benefits in the management of ALI, including extensive knowledge, notable clinical effectiveness, and few toxic adverse reactions [9]. Extracting anti-ALI medications from TCM is highly important as it serves as a valuable reference for the practical use of TCM in treating ALI [10].

The clinical symptoms of ALI diagnosed by Chinese medicine include weakness, bloating, dizziness, and heat, with weakness and bloating being particularly prominent [11,12]. The disease is caused by the invasion of pathogenic factors, specifically wind-warm and lung heat [13]. It is commonly treated with a prescription aimed at clearing heat, eliminating toxins, expelling phlegm, and resolving stasis [14]. Ardisiae Japonicae Herba (AJH) is a dried whole grass of *Ardisia japonica* (Thunb.) Blume, a member of the Myrsinaceae plant family. The herb has a flat shape and possesses a pungent and slightly bitter taste. It is associated with the lung and liver meridians, and is effective in treating conditions such as phlegm, cough, dampness, heat, blood circulation, and blood stasis. It is commonly used in the treatment of respiratory diseases like carbuncles, coughs, bronchitis, and tuberculosis [15,16]. The herb is classified as a remedy for respiratory conditions characterized by excessive heat and inflammation in the lungs. The classic *Materia Medica* discusses the treatment of diaphragmatic gas during illness and the elimination of wind and phlegm. Extracts from the *Compendium of Materia Medica* include “Addressing hemoptysis and occupational wounds, alleviating death caused by fearfulness, and relieving prolonged coughing during labor”. 

Initially, the researchers employed UPLC–Orbitrap Fusion–MS technology to methodically analyze the chemical constituents present in AJH [16]. They also developed a quality control approach to concurrently identify and quantify 31 chemical components in various origins and distinct portions of AJH [17]. The study examined the effects of AJH on ALI model rats, focusing on its pharmacodynamics. The results revealed that AJH exhibited significant anti-inflammatory properties and provided protection against LPS-induced ALI in rats. They found that the therapeutic effect of AJH on ALI was related to regulating the AGE-RAGE, PI3K-AKT, and JAK-STAT signaling pathways [18]. However, the specific pharmacodynamic components of AJH remain unclear, necessitating further investigation. The objective of this study was to fully analyze the chemical contents of various extraction solvents of AJH, tracking, identifying, and evaluating the pharmacodynamic constituents of AJH for the treatment of ALI.

## 2. Results

### 2.1. Composition Analysis

The chemical composition of PE, EA, NB, and WE of AJH were compared to a database and a control sample to obtain primary and secondary information (Table 1). A total of 215, 289, 128, and 69 chemical constituents were identified in each respective extract. After removing duplicate values, a total of 528 constituents were identified. These constituents mainly included 63 phenylpropanoids, 59 terpenes, 35 flavonoids, 28 steroids, 14 quinones, and 329 phenolic acids, carboxylic acids, and their derivatives. Furthermore, PE and EA exhibited a predominant enrichment of terpenoids and steroids, whereas EA demonstrated a primary enrichment of phenylpropanoids and flavonoids. The identification results for the PE, EA, NB, and WS are presented in the Abbreviations.

### 2.2. Active Site Screening

**Figure 1:**
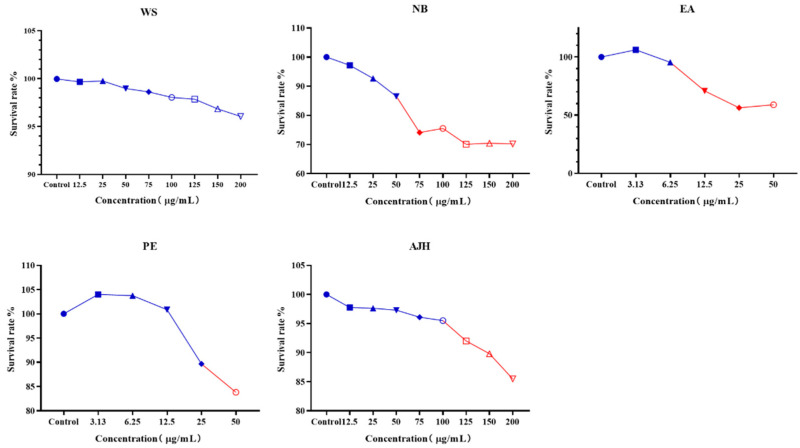
The non-toxic concentration results of different extraction parts of AJH on MH-S cells ($\bar{X}$ ± SD, *n* = 8). The color in blue represent the safe concentration and the red illucidate the harmful concentration. The shape of the points represent always the same mean.

The vitality of MH-S cells was assessed using the CCK-8 assay, and the findings are presented in Figure 1. The concentration range of 3.13–25 μg/mL in the PE group and 12.5–50 μg/mL in the NB group exhibited a more than 85% viability of MH-S cells. However, other concentrations in the PE and the NB had a discernible impact on the growth of MH-S cells. The concentration ranges of 3.13–6.25 μg/mL in the EA group, 12.5–200 μg/mL in the WS group, and 12.5–100 μg/mL in the AJH group had a survival rate exceeding 90% for MH-S cells. This suggested that the concentration ranges mentioned above in the EA, the WS, and the AJH group did not have an impact on the normal growth of MH-S cells and exhibited minimal cytotoxicity. The concentration designed for AJH in the LPS-induced MH-S cell inflammation model is presented in Table 2, based on these findings.

**Figure 2:**
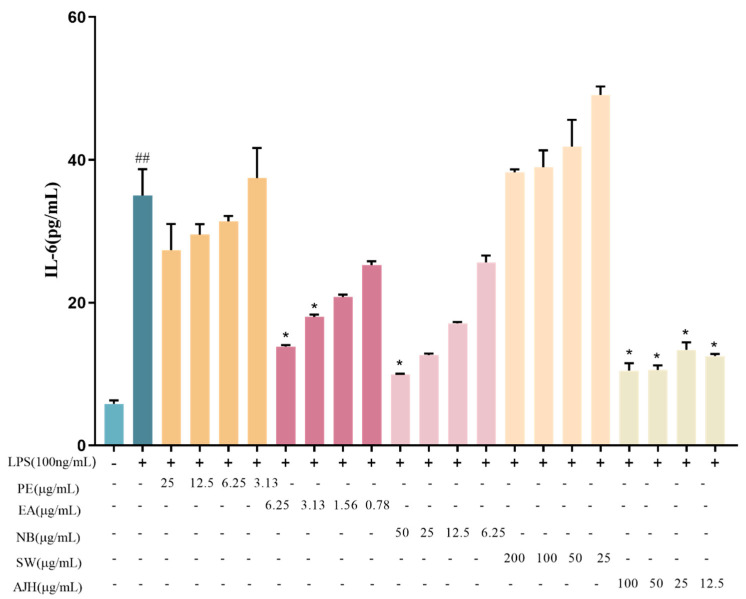
Effects of AJH and different extraction sites on the expression level of IL-6 in MH-S cells ($\bar{X}$ ± SD, *n* = 6). Note: compared with the blank group: ^##^ *p* < 0.01; compared with the model group: * *p* < 0.05. Each color represents a test group, such as PE, EA, NB, WS and AJH.

**Figure 3:**
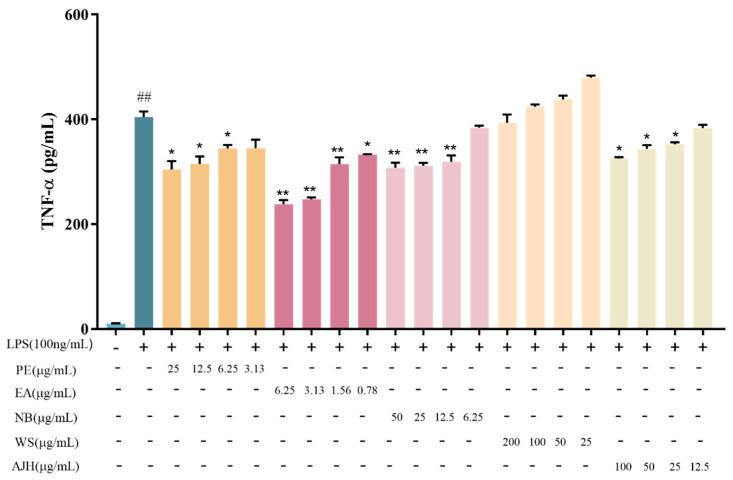
Effects of AJH and different extraction sites on the expression level of TNF-α in MH-S cells ($\bar{X}$ ± SD, *n* = 6). Note: compared with the blank group: ^##^ *p* < 0.01; compared with the model group: * *p* < 0.05, ** *p* < 0.01. Each color represents a test group, such as PE, EA, NB, WS and AJH.

The levels of IL-6 and TNF-α inflammatory factors in the cell supernatant were measured, and the findings are presented in Figure 2 and Figure 3. The expression levels of IL-6 and TNF-α, which are inflammatory factors, were considered equally important. Therefore, a direct summing method was used to calculate a comprehensive score. The *P*_0_ value and the comprehensive score results were calculated for each group to assess the activity of different components of AJH. The results are presented in Table 3.

Figure 2 and Figure 3 demonstrate that the levels of IL-6 and TNF-α in MH-S cells of the model group were markedly higher compared to the blank group, with statistical significance (*p* < 0.01). This suggested the successful establishment of the LPS-induced inflammatory cell model. The four concentrations of PE, EA, and NB exhibited varying degrees of inhibition on the release of IL-6 and TNF-α in MH-S cells. However, the water concentration did not have a significant inhibitory effect on the release of IL-6 and TNF-α. The results in Table 3 demonstrated varying levels of inhibition in the expression of two inflammatory factors, IL-6 and TNF-α, in LPS-induced MH-S cells. The inhibition strength followed the order EA group > AJH group > NB group > PE group > WS group. Therefore, the EA exhibited a stronger anti-inflammatory effect compared to the NB, PE, and WS. Hence, the anti-inflammatory efficacy of EA surpassed that of NB, PE, and WS.

### 2.3. Spectral Effect Relationship Analysis

The concentrations of 19 chemical constituents with significant levels in the EA powder location were measured (μg/g EA powder), and the corresponding components in the raw AJH were also measured (μg/g raw AJH). The content of these components in the EA powder was converted (μg/g raw AJH), indicating that these components are mainly enriched in EA, as presented in Table 4. A gray correlation analysis was conducted to assess the relationship between the expression of IL-6 and TNF-α inflammatory factors. The results, presented in Table 5, revealed a strong correlation between the expression of IL-6 and TNF-α with 17 components, including caryophyllene oxide, bergenin, and gallic acid.

### 2.4. Pharmacodynamic Evaluation

#### 2.4.1. Changes in Lung Function

**Figure 4:**
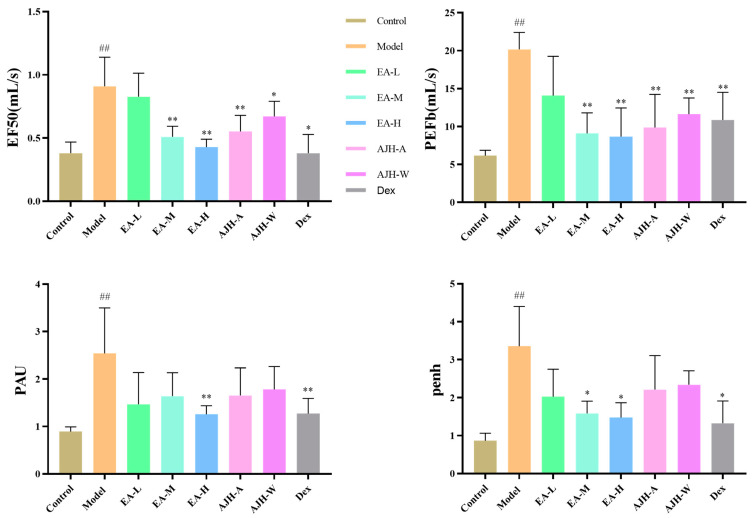
Effects of EA of AJH on the lung function of ALI model rats ($\bar{X}$ ± SD, *n* = 8). Note: comparison with the blank group: ^##^ *p* < 0.01; comparison with the model group: * *p* < 0.05, ** *p* < 0.01. Control: blank group, Model: model group, EA-L: EA low-dose group; EA-M: EA medium-dose group, EA-H: EA high-dose group, AJH-A: AJH 70% alcohol extract group, AJH-W: AJH aqueous extract group, Dex: dexamethasone group.

In comparison to the control group, the model group showed significantly higher levels of EF50, PEFb, PAU, and Penh (*p* < 0.05, 0.01). On the other hand, when compared to the model group, the middle- and the high-dose groups of the EA, the 70% alcoholic extract group of AJH, the aqueous extract group of AJH, and the dexamethasone group exhibited significantly lower levels of PEFb (*p* < 0.01). Additionally, the high-dose group of the EA and the dexamethasone group showed reduced levels of EF50, PAU, and Penh (*p* < 0.05, 0.01). The study demonstrated that the EA played a role in influencing the level of bronchoconstriction and airway resistance in rats with LPS-induced ALI. Furthermore, it greatly enhanced the lung function of rats with ALI, as illustrated in Figure 4.

#### 2.4.2. Changes in Blood Routine

**Figure 5:**
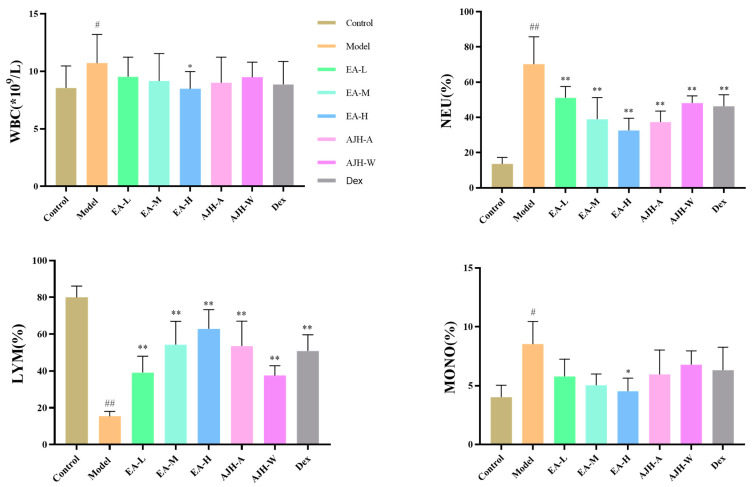
Effects of the EA site of AJH on the blood routine of ALI model rats ($\bar{X}$ ± SD, *n* = 8). Note: comparison with the blank group: ^#^ *p* < 0.05, ^##^ *p* < 0.01; comparison with the model group: * *p* < 0.05, ** *p* < 0.01. Control: blank group, Model: model group, EA-L: EA low-dose group, EA-M: EA medium-dose group, EA-H: EA high-dose group, AJH-A: AJH 70% alcohol extract group, AJH-W: AJH aqueous extract group, Dex: dexamethasone group.

In comparison to the control group, the model group showed a significant increase in peripheral blood white blood cells (WBC), neutrophil percentage (NEU%), and monocyte percentage (MONO%), while lymphocyte percentage (LYM%) significantly decreased (*p* < 0.05, 0.01). When compared to the model group, the ethyl acetate high-dose group exhibited a significant decrease in WBC and MONO%. The EA low-, medium-, and high-dose groups, the AJH 70% alcoholic extract group, and the AJH aqueous extract group showed a significant decrease in NEU% and a significant increase in LYM% (*p* < 0.01). The results are illustrated in Figure 5.

#### 2.4.3. CRP, TNF-α, IL-6, and IL-10 Expression Levels in Serum and Alveolar Lavage Fluid

**Figure 6:**
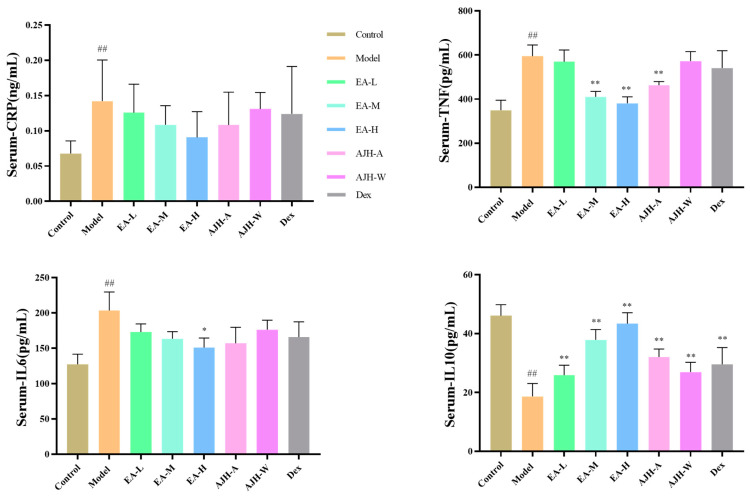
Effects of the EA site of AJH on serum levels of CRP, TNF-α, IL-6, and IL-10 in ALI model rats ($\bar{X}$ ± SD, *n* = 8). Note: comparison with the blank group: ^##^ *p* < 0.01, comparison with the model group: * *p* < 0.05, ** *p* < 0.01. Control: blank group, Model: model group; EA-L: EA low-dose group, EA-M: EA medium-dose group, EA-H: EA high-dose group, AJH-A: AJH 70% alcohol extract group, AJH-W: AJH aqueous extract group, Dex: dexamethasone group.

In comparison to the control group, the model group exhibited a significant increase in the serum levels of TNF-α and IL-6, and a significant decrease in IL-10 (*p* < 0.01). Furthermore, the middle- and high-dose groups of ethyl acetate and the 70% alcoholic extract of the AJH group showed a significant decrease in the serum levels of TNF-α compared to the model group. The high-dose group of ethyl acetate also exhibited a significant decrease in serum levels of IL-6, while IL-10 showed a significant increase (*p* < 0.05, 0.01). There was a tendency to decrease CRP in the serum of the low, medium, and high dose groups of ethyl acetate, the AJH 70% alcoholic extract group, the AJH aqueous extract group, and the dexamethasone group. However, this difference was not statistically significant. These results are illustrated in Figure 6.

In comparison to the control group, the levels of CRP and TNF-α in the BALF of the experimental group showed a significant increase, while the level of IL-10 showed a significant decrease (*p* < 0.01). The level of IL-6 showed a tendency to increase, but the difference was not significant. On the other hand, when compared to the experimental group, the high-dose group of the EA, and the group treated with the 70% alcoholic extract of AJH, the dexamethasone group showed a significant decrease in CRP levels in the BALF (*p* < 0.01). Additionally, the CRP level in the BALF of the experimental group also showed a significant decrease (*p* < 0.01). The levels of TNF-α in BALF were significantly reduced in the EA middle- and high-dose groups, the AJH 70% alcohol extract group, and the dexamethasone group.

**Figure 7:**
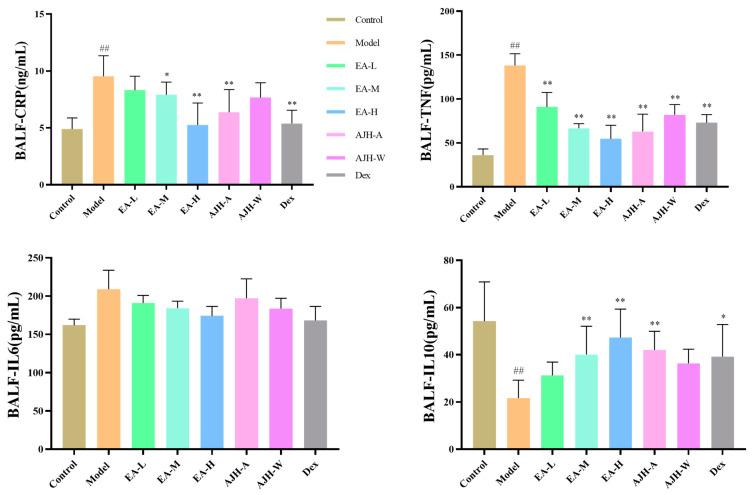
Effects of the EA of AJH on the levels of CRP, TNF-α, IL-6, and IL-10 in the BALF of ALI model rats ($\bar{X}$ ± SD, *n* = 8). Note: comparison with the blank group: ^##^ *p* < 0.01, comparison with the model group: * *p* < 0.05, ** *p* < 0.01. Control: blank group, Model: model group, EA-L: EA low-dose group, EA-M: EA medium-dose group, EA-H: EA high-dose group, AJH-A: AJH 70% alcohol extract group, AJH-W: AJH aqueous extract group, Dex: dexamethasone group.

Conversely, the levels of IL-10 were significantly higher in these groups. There was also a tendency for lower levels of IL-6 in BALF in the EA low-, middle-, and high-dose groups, the AJH 70% alcohol extract group, the AJH aqueous extract group, and the dexamethasone group. However, this difference was not statistically significant. Ultimately, when the dosage of EA increased, the ability of EA to regulate inflammatory variables in ALI model rats also increased in a manner that was dependent on the dosage. These findings are depicted in Figure 7.

#### 2.4.4. Changes in Lung Wet–Dry Weight Ratio and Organ Indexes

Compared with the blank group, the thymus and spleen indices of ALI rats were significantly reduced (*p* < 0.01). The lung wet–dry weight ratio of ALI rats was significantly increased (*p* < 0.01), indicating model animals with severe pulmonary edema.

**Figure 8:**
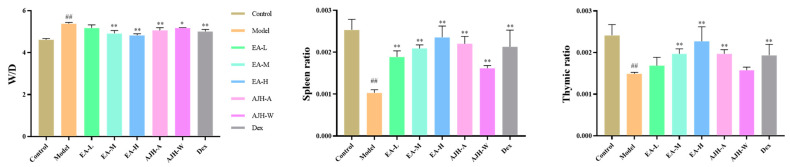
Effects of EA of AJH on lung wet–dry weight ratio and organ index of ALI rats ($\bar{X}$ ± SD, *n* = 8). Note: comparison with the blank group: ^##^ *p* < 0.01; comparison with the model group: * *p* < 0.05, ** *p* < 0.01. Control: blank group, Model: model group, EA-L: EA low-dose group, EA-M: EA medium-dose group, EA-H: EA high-dose group, AJH-A: 70% alcoholic extract group, AJH-W: aqueous extract group, Dex: dexamethasone group.

Compared with the model group, the thymus and spleen indices of rats in the middle- and high-dose groups of EA, the 70% alcoholic extract group of AJH, and the dex group were significantly increased (*p* < 0.01). The wet–dry weight ratio of lungs in the middle- and high-dose groups of ethyl acetate, the 70% alcoholic extract of the AJH group, the aqueous extract of the AJH group, and the dexamethasone group were significantly lower (*p* < 0.05, 0.01). With the increase in the dose of EA, the wet–dry weight of lungs was significantly lower, indicating that the EA had a certain protective effect on the damage of the organs of the ALI rats, and had a certain dose-dependence; the results are shown in Figure 8.

#### 2.4.5. Histopathologic Changes in the Lungs

**Figure 9:**
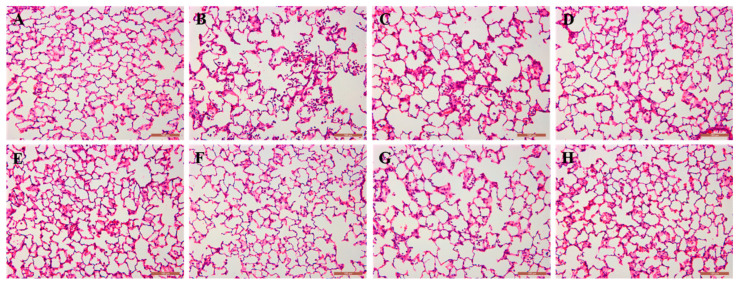
Histopathologic morphology of rat lungs in each group (HE staining, ×200). Note: (**A**): blank group; (**B**): model group; (**C**): low-dose group at EA; (**D**): medium-dose group at EA; (**E**): high-dose group at EA; (**F**): AJH 70% alcoholic extract group; (**G**): AJH aqueous extract group; (**H**): dexamethasone group.

ALI animals exhibited significantly higher levels of inflammatory cell infiltration in the alveolar lumen, a thickening of the alveolar wall, more severe lung injury, and a wider extent of lung injury compared to the control group. In comparison to the model group, the rats in the dexamethasone group and the middle- and high-dose groups of ethyl acetate exhibited significant improvements in lung injury. This suggests that dexamethasone and the middle- and high-dose groups of EA have a protective effect on LPS-induced ALI. The rats in the low-dose group of EA also experienced reduced lung injury, but the effect was relatively weak. These findings are presented in Figure 9.

## 3. Discussion

This study aimed to analyze and characterize the chemical constituents of four extraction sites (PE, EA, NB, and WS) of AJH. The results revealed that the EA had the highest concentration of constituents, followed by the PE, NB, and WS. Phenylpropanoids and flavonoids were predominantly found in the EA, while terpenes and steroids were mainly present in the PE and EA. The NB and WS exhibited relatively lower levels of enrichment compared to the PE and EA. Terpenoids and steroids exhibited higher levels of enrichment in the PE and EA locations, while the NB and WS showed comparatively lower levels of enrichment. Research in the field has demonstrated that flavonoids can mitigate the immune response and diminish inflammatory activation by lowering the levels of expression of inflammatory factors [19,20].

In this study, we employed LPS to stimulate MH-S cells and created a cellular inflammation model [21]. We then examined the impact of AJH, as well as its petroleum ether, ethyl acetate, n-butanol, and water extracts, on the expression levels of IL-6 and TNF-α in these cells. The findings revealed varying degrees of inhibitory effect on the release of IL-6 and TNF-α by AJH and its extracted components. Notably, the ethyl acetate extract exhibited significantly superior inhibitory effects compared to the petroleum ether extract. The anti-inflammatory activity of the EA was markedly superior to that of the PE, NB, and WS. The EA was identified as a potent anti-inflammatory site, which was related to the abundance of phenolpropanoids and flavonoids [22,23].

The chemical constituents of different parts of the AJH were characterized, and the anti-inflammatory activity in vitro was studied. Based on the results and the related literature, 19 components were selected for testing in the ethyl acetate part of AJH. The analysis showed that the contents of these 19 components were not significantly different between the ethyl acetate part and the whole AJH herb. Furthermore, 19 components were found to be mostly concentrated in the EA. The gray correlation analysis revealed the presence of 17 components, such as caryophyllene oxide, bergenin, gallic acid, quercetin, and epigallocatechin gallate, in the EA. These components showed a strong correlation with anti-inflammatory activity, making them suitable for the quality control of AJH. Studies have demonstrated that bergenin could effectively enhance lung injury [24] in mice infected with Klebsiella. It achieved this by suppressing the production of inflammatory cytokines, including IL-6, TNF-α, IL-1β, and PEG2, reducing the levels of MPO and MDA, and increasing the activities of SOD and GSH. These effects are mediated through the NF-κB [25] and MAPKs pathways, resulting in anti-inflammatory effects on Klebsiella-infected mice [26]. Quercetin [27,28], rutin [29,30], and kaempferide [31], all flavonoids, have anti-inflammatory properties to ameliorates acute lung injury. Khazdair Mohammad et al. demonstrated that quercetin can reduce the levels of reactive oxygen species, as well as the expression of inducible nitric oxide synthase and pro-inflammatory mediators such as TNF-α, IL-1α, IL-1β, IL-6, IL-10, and IL-12. These effects suggest that quercetin and kaempferide may have therapeutic potential against SARS-CoV-2 [32].

**Figure 10:**
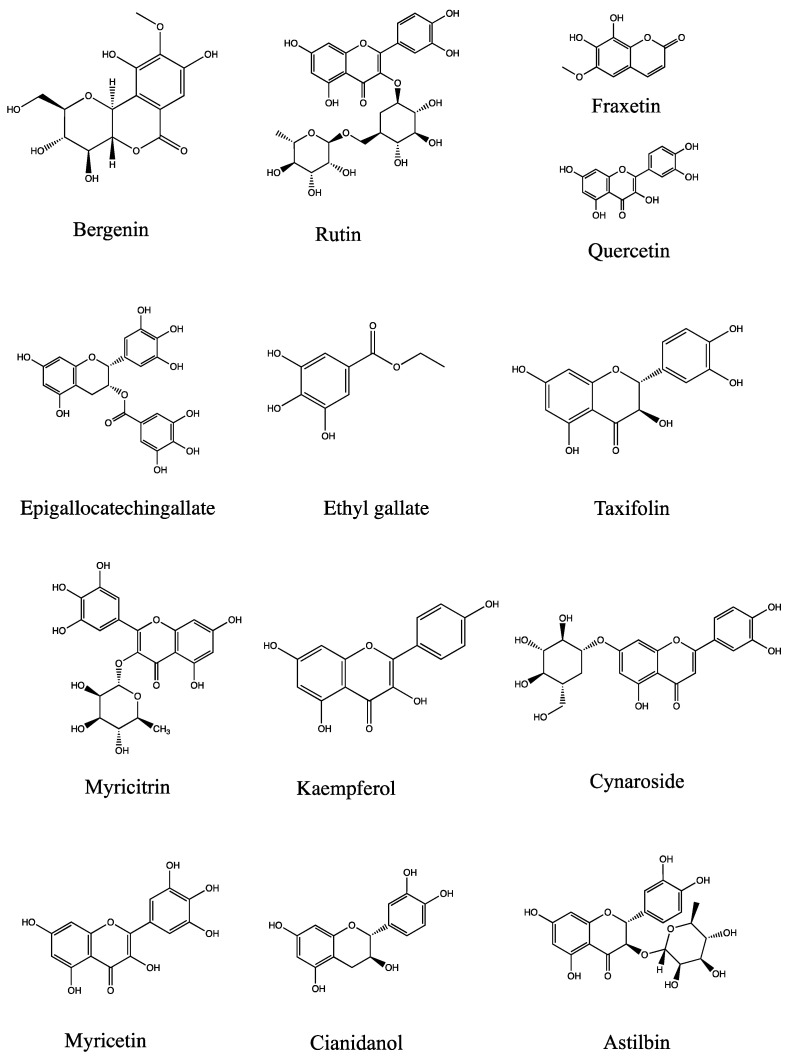
Chemical structure of 13 main compounds (bergenin, quercetin, epigallocatechingallate, rutin, cianidanol, myricitrin, ethyl gallate, kaempferol, taxifolin, cynaroside, myricetin, fraxetin, astilbin).

The pharmacodynamic effects of the ethyl acetate component of AJH on ALI rats were evaluated by examining lung function, blood routine, inflammatory factors, and other pharmacodynamic indexes. The results demonstrated that the ethyl acetate component improved lung injury, suppressed the expression of pro-inflammatory factors, and enhanced the release of anti-inflammatory factors in ALI rats. Furthermore, the intervention effect of the ethyl acetate component on ALI rats was found to be dependent on the dosage. Based on the examination of lung tissue samples, it was shown that the administration of ethyl acetate at high, medium, and low doses resulted in varying degrees of improvement in the infiltration of inflammatory cells surrounding the alveolar lumen and bronchial wall in rats with ALI. The administration of ethyl acetate at low, medium, and high doses had varying degrees of positive effects on the rats’ apnea (PAU) and the medium and high doses of AJH ethyl acetate were particularly effective in improving the rats’ maximum expiratory flow rate (PEFb) and airway narrowing index (Penh). Furthermore, the high-dose group of AJH ethyl acetate showed superior efficacy compared to the AJH group. The blood analysis showed that administering a high dose of the ethyl acetate component of AJH resulted in a significant decrease in WBC and MONO%, while the low, medium, and high doses reduced the neutrophil ratio (NEU%) and increased the lymphocyte ratio (LYM%) in rats. These findings suggested that the ethyl acetate component had anti-inflammatory properties and enhanced the immune system. Furthermore, the concentration of TNF-α in the serum samples from the EA was significantly decreased in the middle- and high-dosage groups. The concentration of IL-6 in the serum was significantly decreased in the high-dosage group. The concentrations of IL-10 in both the serum and BALF were significantly increased. Additionally, the concentration of CRP in BALF was significantly decreased. Hence, the ethyl acetate compound exhibits a specific therapeutic impact on rats with ALI, demonstrating a clear dependence on dosage. The serum pharmacochemistry of TCM was designed to screen the efficacy material base of TCMs from the constituents absorbed into the blood after oral administration [33,34]. Thus the hypothesis suggests that the pharmacodynamic components of AJH, including bergenin, quercetin, epigallocatechingallate, rutin, cianidanol, myricitrin, ethyl gallate, kaempferol, taxifolin, cynaroside, myricetin, fraxetin, astilbin, and 13 other components (Figure 10), may have an effect when combined with serum medicinal chemistry [18]. However, further research is needed to determine the specific mechanisms of action.

## 4. Materials and Methods

### 4.1. Materials and Instruments

The herbs were purchased from Anhui Sheng’Antang Drug Co., Ltd. (Longhui, Hunan, China, batch no. 210301), and identified by Prof. Chen Suiqing of the Henan University of Traditional Chinese Medicine as the dried whole plant of AJH. Gallic acid, fraxetin, and kaempferide were purchased from Chengdu Manstar Biotechnology Co., Ltd. (Chengdu, China) and Chengdu Kroma Biotechnology Co. (Chengdu, China) Caryophyllene oxide (batch no. J16GB155133, purity ≥ 90%) and taxifolin were purchased from Shanghai Yuanye Biotechnology Co. (Shanghai, China) (batch no. C15J11Q107526, purity ≥ 98%). Petroleum ether (batch no. 20220108, Tianjin Jindong Tianzheng Fine Chemical Reagent Factory, analytical pure), ethyl acetate (batch no. 20210820, Tianjin Hengxing Chemical Reagent Manufacturing Co., Ltd. (Tianjin, China), analytical pure), *n*-butanol (batch no. 20210104, Tianjin Hengxing Chemical Reagent Manufacturing Co., Ltd. (Tianjin, China), batch no. F22M6O102), mass spectrometry grade acetonitrile (Thermo Fisher Scientific Co., Ltd. (Shanghai, China) batch no. F22M8L203), mass spectrometry grade formic acid (Thermo Fisher Scientific Co., Ltd. (Shanghai, China), batch no. 214911), lipopolysaccharides (LPS, batch no. 039M4004V, Sigma-Aldrich, St. Louis, MO, USA), Mouse IL-6 reagent kit (batch no. A206H20634, Hangzhou C&T Biotechnology Co., Ltd. (Hangzhou, China)), Mouse TNF-α kit (batch no. UX0448RH0115, Wuhan Elite Bioscience and Technology Co., Ltd. (Wuhan, China)), CCK-8 (batch no. GK100011, Shanghai Hongye Bio-Technology Co., Ltd. (Shanghai, China)), fetal bovine serum (batch no. 21050701, Zhejiang Ltd. (Hangzhou, China)), RPMI 1640 medium (batch no. 20220424, Beijing Solebao Technology Co., Ltd. (Beijing, China)), and mouse alveolar macrophage (MH-S cell) lines were purchased from Procell Life Science&Technology Co., Ltd. (Wuhan, China) We also used UPLC–Orbitrap Fusion–MS (Thermo Scientific, Waltham, MA, USA), MS105DU one-hundred-thousandth analytical balance (Mettler Instruments Ltd., Greifensee, Switzerland), Heraeus Multifuge X_1_R refrigerated tabletop high-speed centrifuge (Thermo Scientific, Waltham, MA, USA), Model SPD2010-230 Centrifugal Cryoconcentrator (Thermo Scientific, Waltham, MA, USA), Milli-QPOD Ultrapure Water Preparator (Millipore, Darmstadt, Germany), inverted microscope (ZEISS, Oberkochen, Germany), carbon dioxide incubator (Thermo Scientific, Waltham, MA, USA), and multi-function enzyme labeling instrument (Berten Instruments Ltd., Burlington, VT, USA).

### 4.2. Analysis of Components in Different Polar Parts of AJH

#### 4.2.1. Sample Preparation

Take 2500 g of dried AJH and add 12 times the amount of 70% ethanol. Let it soak in the cold for 12 h, then heat it three times for 2 h each for the reflux. Filter the mixture and combine the three extracts. Let it sit for 12 h, allowing the ethanol to evaporate and remove any alcohol flavor. Concentrate the mixture under reduced pressure until it reaches the desired volume of 1500 mL. This will yield the extract of the AJH herbs. The AJH was subjected to sequential extraction using three organic solvents, namely petroleum ether, ethyl acetate, and *n*-butanol [35,36], in a 1:1.5 volume ratio. This extraction process was repeated five times. The extracts obtained using the same solvent were combined and concentrated under reduced pressure. The resulting concentrate was then freeze-dried to obtain powdered extracts of different parts of the AJH: petroleum ether site (PE, 26.45 g), ethyl acetate site (EA, 45.75 g), *n*-butanol site (NB, 71.80 g), and water site (WS, 169.40 g). The powder yields of the four components were 1.06%, 1.83%, 2.87%, and 6.78%, respectively. The powders from various components were measured and treated with ultrasound in methanol. The solutions from different components were created, filtered through a 0.22 μm microporous membrane, and examined using mass spectrometry.

#### 4.2.2. Mass Spectrometry

The chromatographic conditions used in this experiment were as follows: a Hypersil GOLD C_18_ column with dimensions of 2.1 mm × 100 mm and a particle size of 3 μm was employed. The mobile phase consisted of methanol (A) and a 0.1% aqueous solution of formic acid (B). A gradient elution method was employed, with the following time intervals and corresponding changes in solvent composition: 0–2 min (95–75% B), 2–10 min (75–20% B), 10–22 min (20–12% B), 22–24 min (12–11.5% B), and 24–25 min (11.5–0% B). The flow rate was set at 0.2 mL/min. The column temperature was maintained at 25 ℃. The sample injection volume was 5 μL.

Mass spectrometry was conducted under the following conditions: electrospray ionization (ESI) was used, with nitrogen as the carrier gas. The sheath gas flow rate was set at 35 Arb. The auxiliary gas flow rate was set at 5 Arb. The spray voltage was 3.50 kv for positive ions and 2.80 kv for negative ions. The ion transfer tube was maintained at a temperature of 300 ℃, while the gasification temperature was set at 275 °C. The scanning mode used was Full MS/dd-MS^2^ mode, which detected positive and negative ions separately. The first-stage full-scanning resolution was 120,000, with a scanning range of *m/z* 100–1000. The secondary scanning resolution was 60,000, and the HCD collision energy was set at 35%.

#### 4.2.3. Mass Spectrometry Data Analysis

The raw data gathered were integrated into Compound Discoverer version 3.3 software. The procedure of identifying and analyzing unknown compounds was established using databases such as Chemspider (http://www.chemspider.com, accessed on 30 December 2023) and mzCloud (https://www.mzcloud.org/, accessed on 30 December 2023) [37,38]. The raw data underwent peak alignment and peak extraction. The information from the characteristic fragmentation ion peaks was synthesized and analyzed. Xcalibur software version 4.0 was utilized to calculate the possible chemical compositions with an error of less than 5 ppm. Additionally, Mass Frontier software was employed to infer the structure by examining the cleavage pattern [39]. This allowed for the qualitative identification of the chemical compositions of the four extracted parts of AJH.

### 4.3. Analysis of the Activity of Different Parts of AJH

#### 4.3.1. Preparation of Reserve Solution of Different Extracted Parts of AJH

Measure 5 g of AJH powder. Combine it with 100 mL of 70% ethanol for reflux extraction for 2 h. Filter the mixture and then evaporate the resulting filtrate until it becomes a powder. Measure 20 mg of the extracted powder from AJH. Add 400 mL of dimethyl sulfoxide (DMSO) to dissolve the powder. This will result in a concentration of 50 mg/mL for the solution. Measure 20 mg of powdered AJH for each of the following solvents: petroleum ether, ethyl acetate, n-butanol, and water. Add 400 µL of DMSO to each sample to create a stock solution with a concentration of 50 mg/mL.

#### 4.3.2. Mapping of Administration Concentration

The MH-S cells were cultivated in a cell culture incubator at 37 °C, 5% CO_2_, and saturated humidity using RPMI-1640 media supplemented with 10% fetal bovine serum. Once the cell fusion degree reached 80–90%, the cells were transferred to a new culture dish, and only cells in the logarithmic growth phase were used for the studies. The cell suspension was diluted to a density of 2.5 × 10^6^ cells/mL using RPMI-1640 media. The cells were then added to 96-well culture plates at a volume of 100 μL per well. Subsequently, the plates were placed in an incubator and grown for 24 h. The initial culture solution was discarded, and the cells were allocated into several groups: a control group, a group treated with varying concentrations of AJH extracts, a group treated with varying concentrations of petroleum ether, a group treated with varying concentrations of acetic acid, and a group treated with varying concentrations of acetate. The control group was supplemented with a medium containing 10% fetal bovine serum. The other groups received varying concentrations of drugs. Six wells were prepared for each experimental group. After drug administration, incubation was carried out for 24 h. Following this, the CCK-8 reagent solution was added and incubated for 3 h in the incubator. Finally, the enzyme marker was introduced into the incubator. Following drug administration, the cells were placed in an incubator for 24 h. Subsequently, a solution of CCK-8 reagent was given to the cells. They were further cultured in the incubator for 3 h. The absorbance of each group was quantified at a wavelength of 450 nm using an enzyme marker. The cell viability of each group was determined, and is shown in Table 6.

#### 4.3.3. Expression of IL-6 and TNF-α in MH-S Cells

The administration concentration was determined based on the results from screening the administration concentration of each site of MH-S cells using the CCK-8 technique. Cells in the logarithmic growth phase, namely MH-S cells, were used for digestion. The density of the cell suspension was adjusted to 1 × 10^5^ cells/mL. Next, 1 mL of the cell suspension was inoculated into each well of 12-well plates. The plates were then placed in a cell incubator and cultivated for 24 h. The cells were divided randomly into several groups: the control group, the LPS group, the LPS+AJH group, the LPS+ group, the LPS+EA group, the LPS+NB group, and the LPS+WS group. The control group was treated with medium containing 10% fetal bovine serum. The LPS group and the LPS+ administration group were induced to create the MH-S cell inflammation model by adding 100 ng/mL of LPS. After adding the drug, the cells were incubated for 24 h. The cell supernatants were then collected to measure the levels of IL-6 and TNF-α inflammatory factors.

The study also introduced the parameter *p*_0_ value to comprehensively evaluate the anti-inflammatory activity of the four extracted portions of AJH. The equation *p*_0_ = $\frac{P2}{P1}$ represents the relationship between *p*-values in the context of one-way ANOVA. *p*_1_ refers to the *p*-value obtained from comparing each dosing group to the model group. A smaller *p*_1_ value indicates a greater difference between each dosing group and the model group. On the other hand, *p*_2_ represents the *p*-value obtained from comparing each dosing group to the blank group. A larger *p*_2_ value suggests that the inhibition of inflammatory factor release in each dosing group after administration is more like that of the blank group. The expression of IL-6 and TNF-α inflammatory factors is the same. To analyze the active components of AJH, we utilized direct summing to compute comprehensive scores for each group based on the expression of IL-6 and TNF-α inflammatory factors. Additionally, we evaluated the *p*_0_ value and comprehensive scoring results for each group, considering both factors equally important.

### 4.4. Study on the Spectrum-Effect Relationship between the Anti-Inflammatory Activities of the Active Parts of AJH

#### 4.4.1. Preparation of Test Solution

Weigh precisely 0.1 g of AJH powder. Heat it twice in methanol under reflux for 1 h each time. Filter the mixture and combine the filtrate. Concentrate and evaporate the solution. Redissolve the residue in methanol and adjust the volume to 10 mL using a volumetric flask. Centrifuge the solution at 14,000 r/min for 15 min and collect the supernatant. Filter the supernatant using a 0.22 μm micropore filter membrane to obtain the test solution of AJH. Take approximately 0.009 g of the ethyl acetate component from AJH powder. Use precise weighing techniques. Add 50 mL of methanol and subject it to ultrasonic treatment for 10 min. Compensate for any weight loss by adding more methanol. Filter the mixture and then centrifuge it at a speed of 14,000 revolutions per minute for 15 min. Collect the liquid portion (supernatant) and pass it through a 0.22 μm micropore filter membrane. This will yield the ethyl acetate component of AJH, which could be used as the test solution.

To ensure accuracy, the following substances were carefully weighed: gallic acid, ethyl gallate, fraxetin, palmitic acid, kaempferide, eriodictyol, catechin, bergenin, epicatechin gallate, luteolin-7-*O*-glucoside, astilbin, myricitrin, nicotiflorin, rutin, isoeugenol, caryophyllene oxide, quercetin, taxifolin, and myricetin. Additionally, a control solution was prepared by adding an appropriate amount to a 2 mL volumetric flask and filling it with methanol. The solution was then thoroughly mixed. Thoroughly agitate the control stock solution and store it in a refrigerator at a temperature of 4 °C for future use. Before usage, prepare the mixed standard solution with an appropriate concentration.

#### 4.4.2. Mass Spectrometry

The chromatographic conditions used in this study were as follows: a Hypersil GOLD C_18_ column with dimensions of 2.1 mm × 100 mm and a particle size of 3 μm was employed. The mobile phase consisted of a mixture of methanol (A) and a 0.1% aqueous solution of formic acid (B). A gradient elution method was employed, with the following time intervals and corresponding percentages of B: 0–2 min (95% B), 2–10 min (95–60% B), 10–12 min (60–30% B), 12–18 min (30–5% B), and 18–21 min (30–30% B). The composition of the sample was 5% B for 18–21 min, 5% B for 21–22 min, and 95% B for 22–25 min. The flow rate was 0.2 mL/min, the column temperature was 25 ℃, and the injection volume was 1 μL.

Mass spectrometry was performed under the following conditions: electrospray ionization source (ESI), nitrogen as the carrier gas, a sheath gas flow rate of 25 Arb, an auxiliary gas flow rate of 10 Arb, a spray voltage of 3.50 kv (+) and 2.50 kv (−), an ion transfer tube temperature of 325 ℃, and a gasification temperature of 325 °C. The scanning mode used was SIM mode, which involved the switching of positive and negative ions. The resolution of the scanning mode was 60,000. Table 7 displayed the ion information of the components that were measured for quantitative analysis.

#### 4.4.3. Correlation Analysis of “Anti-Inflammatory Activity-Constituents” in the EA of AJH

A gray correlation study was conducted using the SPSS PRO program [40]. The raw data were transformed into a dimensionless format. The expression levels of IL-6 and TNF-α inflammatory factors were designated as the parent sequence Y(k), while the results of determining the content of the 19 components in the ethyl acetate part of AJH were designated as the subsequence Xi(k), where i represents the component. The absolute difference sequence Δi(k) was defined as the absolute value of the difference between the elements of the parent sequence Y(k) and the subsequence Xi(k). The calculation of the correlation coefficient is as follows:

Ai(k) = [Δi(k)min + ρ × Δi(k)max]/[Δi(k) + ρ × Δi(k)max]

The discrimination coefficient, denoted as ρ, is defined in this experiment as a value between 0 and 1. In this case, ρ was set to 0.5. A correlation coefficient greater than 0.8 indicated a strong relationship between the subsequence and the parent sequence. A correlation coefficient between 0.6 and 0.8 indicated a moderate relationship, while a correlation coefficient less than 0.6 indicated a weak relationship [41].

### 4.5. Pharmacological Activities of the Ethyl Acetate Parts of AJH

#### 4.5.1. Preparation of AJH and Its Related Medicinal Fluid

Take 500 g of AJH herbs, combine with 12 times the volume of water, and perform a heating and reflux extraction process. The filtrate is then concentrated under reduced pressure three times, with each concentration lasting for 2 h. This process results in a final concentration of 2 g of AJH per milliliter, which is the water extract of AJH. Take 500 g of AJH herbs and add 12 times the volume of 70% ethanol. Heat the mixture and carry out a reflux extraction. The filtrate is subjected to decompression and concentration three times, with each iteration lasting for 2 h, to remove any alcohol flavor. The resulting concentrate will have a concentration of 2 g of AJH per milliliter, equivalent to a 70% alcohol extract of AJH. To prepare the ethyl acetate portion of AJH powder, follow the same procedure as described in Section 4.2.1. Weigh the powder and then add a 0.5% solution of CMC-Na to create a suspension equivalent to 2 g of AJH/mL. This suspension will serve as the ethyl acetate portion of the AJH liquid.

#### 4.5.2. Animal Administration and Sample Collection

Male Sprague Dawley rats were acclimated and provided with food for 7 days (the Approval Code is DWLLGZR202303103 and the Approval Date is 1 March 2023. All animal experiments also complied with ethical guidelines for the use of animals in research according to policies of the UK). The participants were divided into 8 groups (*n* = 8) through a random process. These groups included a blank group, a model group, a low-dose group receiving 5 g of AJH raw drug/kg/d of ethyl acetate, a medium-dose group receiving 10 g of AJH raw drug/kg/d of ethyl acetate, a high-dose group receiving 20 g of AJH raw drug/kg/d of ethyl acetate, a group receiving AJH 70% alcoholic extract at a dose of 20 g/kg/d, a group receiving AJH aqueous extract at a dose of 20 g/kg/d, and a dexamethasone group receiving a dose of 2.3 × 10^−4^ g/kg/d. The low, medium, and high doses of EA, 70% alcoholic extract of the AJH group, aqueous extract of the AJH group, and the dexamethasone administration group were orally administered with their respective doses. The blank and model groups were orally administered with the same dose of distilled water for 7 consecutive days.

After 1 h following the final dose, the rats were rendered unconscious by injecting 20% ursodiol into their peritoneal cavity. The ALI rat model was induced by administering lipopolysaccharide (LPS) by a tracheal drip at a dosage of 2 mg/kg in each experimental group, except for the control group, which received a tracheal drip of saline. Following the rats’ natural awakening and a 24 h fasting period, blood samples were obtained from the abdominal aorta. One portion of the blood was collected using a lithium heparin negative-pressure tube to assess routine pharmacodynamic indexes of blood, including white blood cell count (WBC), neutrophil ratio (NEU%), lymphocyte ratio (LYM%), and monocyte ratio (MONO%). The other portion of the blood serum was collected in a negative-pressure tube without sodium heparin and left in the tube for 2 h. Subsequently, the serum was subjected to centrifugation at a temperature of 4 °C for 15 min. Following this, it was transferred to a negative-pressure tube devoid of sodium heparin and left undisturbed for 2 min. The other portion was placed in a negative pressure tube without sodium heparin to collect serum. It was then left for 2 h and centrifuged at 4 °C for 15 min at a speed of 3000 revolutions per minute. The resulting supernatant was used to detect the levels of IL-6, IL-10, CRP, and TNF-α inflammatory factors. The trachea and whole lungs of the rats were removed by opening the chest. The bronchoalveolar lavage fluid (BALF) from the left lungs was withdrawn after ligating the right main bronchus, and was also used to detect the levels of IL-6, IL-10, CRP, and TNF-α. The left lungs were then perfused with 4% paraformaldehyde for 30 min. The tissue samples were treated with paraformaldehyde for 30 min and then fixed in a 4% paraformaldehyde solution after perfusion. These samples were intended for pathological examination. The upper lobes of the right lung, spleen, and thymus were rinsed with saline solution. Excess surface water was removed using filter paper. The samples were then weighed to determine the lung wet/dry weight ratio and organ indexes.

#### 4.5.3. Testing Indicators

##### Lung Function

After 24 h following the end of modeling, the animal whole-body volume scanning detection system non-invasive (WBP) was used to detect the lung function parameters of each group of rats, including airway narrowing index (Penh), maximal expiratory flow rate (PEF), expiratory flow rate in mid-expiration (EF50), and apnea (PAU) [42].

##### Blood Routine

Blood was taken from the abdominal aorta for routine blood tests to count white blood cell count (WBC), neutrophil ratio (NEU%), lymphocyte percentage (LYM%), and monocyte percentage (MONO%).

##### Measurement of IL-10, IL-6, CRP, and TNF-α Levels in Serum and BALF

An enzyme-linked immunosorbent assay (ELISA) was used to determine IL-10, IL-6, CRP, and TNF-α expression according to the respective manual in serum and BALF.

##### Lung Wet/Dry Weight Ratio and Organ Index

The upper lobes of the right lung of the rats in each group were excised and weighed to obtain the wet weight (W), then placed in the oven at 60 °C for 48 h and weighed to obtain the dry weight (D). The degree of edema of lung tissue was evaluated by calculating the ratio of W/D. The thymus and spleen of rats in each group were taken and weighed to calculate the rat organ index, and the calculated results were statistically analyzed.

##### Histopathological Analysis of Lungs

The left lung was fixed with 4% paraformaldehyde, routinely dehydrated, paraffin-embedded, sectioned at 4 μm, and routinely HE-stained, and the pathological changes in the lung tissues of rats in each group were observed under the microscope.

### 4.6. Data Analysis

All data were presented as mean ± SD ($\bar{X}$ ± SD) and analyzed by a one-way analysis of variance, followed by Tukey’s multiple comparisons as a post hoc test. All statistical analyses and graphs were performed using GraphPad Prism version 7.0 software (GraphPad Software, Inc., San Diego, CA, USA). *p* values less than 0.05 were considered statistically significant.

## 5. Conclusions

This study determined the chemical compositions of several solvent extraction methods in AJH. There were a total of 528 chemical components examined. Within the petroleum ether, ethyl acetate, *n*-butanol, and aqueous environments, a total of 215, 289, 128, and 69 components were identified, respectively. The research revealed that the ethyl acetate extraction site consisted of a high concentration of flavonoids, phenylpropanoids, and quinones. Furthermore, the anti-inflammatory activities of the four solvents of AJH were evaluated using the cellular tests conducted. The findings demonstrated that the EA site of AJH shown significant efficacy in anti-inflammatory treatment. Hence, a comprehensive analysis was conducted on a total of 19 chemical constituents at the EA site. The gray relational analysis was used to examine the correlation between 19 constituents and their anti-inflammatory activity. A total of 17 substances have been identified as pharmacodynamic components with anti-inflammatory properties. Relying on prior studies on serum medicinal chemistry, a total of 13 components were ultimately recognized as having biological activity in AJH for the purpose of treating acute lung injury. Additionally, the intermediate and high doses of the EA site of AJH exhibited a more significant effect in enhancing lung function, blood routine, and lung histology.

## Figures and Tables

**Table 1:** Compositional enrichment results of PE, EA, NB, and WS.

Sites	Phenylpropanoid	Terpene	Flavonoid	Steroid	Quinone	Phenolic Acids, Carboxylic Acids, and Their Derivatives	Total
PE	23	33	4	16	5	134	215
EA	40	31	31	13	7	167	289
NB	21	13	7	1	5	81	128
WS	11	3	3	2	1	49	69
Total	63	59	35	28	14	329	528

**Table 2:** Design of optimal dosing concentration for each dosing group (μg/mL).

Sites	1	2	3	4
AJH	100	50	25	12.5
PE	25	12.5	6.25	3.13
EA	6.25	3.13	1.56	0.78
NB	50	25	12.5	6.25
WS	200	100	50	25

**Table 3:** Comprehensive scoring results of PE, EA, NB, and WS on the expression levels of IL-6 and TNF-α in AJH.

Sites	IL-6				TNF-α				*P*_0*IL*-6_ + *P*_0*TNF-α*_
	*p*_1_	*p*_2_	*p*_0_	SUM*P*_0_	*p*_1_	*p*_2_	*p*_0_	SUM*P*_0_	
PE 25 μg/mL	0.374	0.039	0.104	0.136	0.01	0.004	0.400	0.806	0.942
PE 12.5 μg/mL	0.475	0.003	0.006		0.01	0.003	0.300		
PE 6.25 μg/mL	0.740	0.000	0.000		0.018	0.001	0.056		
PE 3.13 μg/mL	0.997	0.025	0.025		0.06	0.003	0.050		
EA 6.25 μg/mL	0.031	0.014	0.452	0.525	0.001	0.002	2.000	2.429	2.954
EA 3.13 μg/mL	0.039	0.002	0.051		0.003	0.000	0.000		
EA 1.56 μg/mL	0.059	0.001	0.017		0.007	0.003	0.429		
EA 0.78 μg/mL	0.184	0.001	0.005		0.032	0.000	0.000		
NB 50 μg/mL	0.043	0.001	0.023	0.023	0.003	0.002	0.667	0.952	0.975
NB 25 μg/mL	0.065	0.000	0.000		0.005	0.000	0.000		
NB 12.5 μg/mL	0.091	0.000	0.000		0.007	0.002	0.286		
NB 6.25 μg/mL	0.181	0.000	0.000		0.333	0.000	0.000		
WS 200 μg/mL	−0.807	0.000	0.000	−0.039	−0.017	0.002	0.118	−0.118	−0.157
WS 100 μg/mL	−0.794	0.006	0.008		−0.1	0.000	0.000		
WS 50 μg/mL	−0.483	0.015	0.031		−0.335	0.000	0.000		
WS 25 μg/mL	−0.076	0.000	0.000		−0.976	0.000	0.000		
AJH 100 μg/mL	0.024	0.037	1.542	2.127	0.02	0.000	0.000	0.059	2.186
AJH 50 μg/mL	0.029	0.005	0.172		0.017	0.001	0.059		
AJH 25 μg/mL	0.031	0.012	0.387		0.045	0.000	0.000		
AJH12.5 μg/mL	0.038	0.001	0.026		0.315	0.000	0.000		

**Table 4:** Results of the determination of the content of AJH and EA ($\bar{X}$ ± SD, *n* = 3).

Compounds	EA	AJH (Obversion)	AJH
	Content (μg/g EA Powder)	Content (μg/g Raw AJH)	
Isoeugenol	758.18 ± 4.75	13.98 ± 0.11	100.33 ± 0.02
Caryophyllene oxide	453.91 ± 1.84	8.27 ± 0.1	81.32 ± 1.54
Quercetin	6057.59 ± 71.5	112.15 ± 0.08	155.49 ± 1.93
Taxifolin	662.33 ± 8.79	12.07 ± 0.21	11.18 ± 0.07
Myricetin	4521.58 ± 9.77	83.15 ± 0.89	80.89 ± 0.25
Gallic acid	8300.42 ± 23.04	153.48 ± 0.35	174.36 ± 1.01
Ethyl gallate	1398.6 ± 1.67	24.24 ± 1.28	30.86 ± 0.03
Fraxetin	918.08 ± 10.3	16.34 ± 0.09	18.34 ± 0.12
Palmitic acid	15,525.36 ± 39.26	282.86 ± 0.61	269.68 ± 0.06
Kaempferol	23,916.89 ± 42.79	439.05 ± 2.34	476.97 ± 1.09
Eriodictyol	446.7 ± 1.78	8.23 ± 0.04	8.2 ± 0.03
Cianidanol	2897.69 ± 6.14	52.52 ± 0.01	49.02 ± 0.1
Bergenin	285,582.94 ± 2309.97	5064.71 ± 59.58	5150.12 ± 23.68
Cynaroside	17,346.17 ± 88.96	318.37 ± 1.35	311.3 ± 0.36
Astilbin	2343.77 ± 1.24	43.11 ± 0.16	54.9 ± 0.09
Epigallocatechingallate	12,936.53 ± 55.07	238.32 ± 0.14	234.04 ± 0.69
Myricitrin	12,030.85 ± 8.51	220.46 ± 0.28	265.71 ± 4.68
Nictoflorin	397.4 ± 2.15	7.27 ± 0.03	8.71 ± 0.02
Rutin	618.27 ± 0.82	11.28 ± 0.02	6.73 ± 0.03

Note: 2500 g of raw AJH could yield 45.75 g of EA powder.

**Table 5:** Results of the correlation between the content of 19 chemical components in the EA and anti-inflammatory activity.

Compounds	Correlation	Range	Compounds	Correlation	Range
Caryophyllene oxide	1	1	Taxifolin	0.833	11
Bergenin	0.878	2	Isoeugenol	0.831	12
Gallic acid	0.863	3	Palmitic acid	0.828	13
Quercetin	0.86	4	Luteolin-7-*O*-glucoside	0.824	14
Epigallocatechin gallate	0.846	5	Myricetin	0.824	15
Rutin	0.84	6	Fraxetin	0.821	16
Catechin	0.84	7	Astilbin	0.82	17
Myricitrin	0.839	8	Eriodictyol	0.753	18
Ethyl gallate	0.836	9	Nicotiflorin	0.626	19
Kaempferide	0.835	10			

**Table 6:** Different concentration designs of dosing groups (μg/mL).

Sites	1	2	3	4	5	6	7	8
AJH	200	150	125	100	75	50	25	12.5
PE	50	25	12.5	6.25	3.13	-	-	-
EA	50	25	12.5	6.25	3.13	-	-	-
NB	200	150	125	100	75	50	25	12.5
WS	200	150	125	100	75	50	25	12.5

**Table 7:** Ion information of the components to be measured for quantitative analysis.

Compounds	Test Mode	Test Ion	*m*/*z*	Rt (min)
Isoeugenol	[M + H]^+^	C_10_H_13_O_2_^+^	165.0910	15.73
Caryophyllene oxide	[M + H]^+^	C_15_H_25_O^+^	221.1900	17.01
Quercetin	[M + H]^+^	C_15_H_11_O_7_^+^	303.0499	8.67
Taxifolin	[M + H]^+^	C_15_H_13_O_7_^+^	305.0656	9.19
Myricetin	[M + H]^+^	C_15_H_11_O_8_^+^	319.0448	8.86
Gallic acid	[M − H]^−^	C_7_H_5_O_5_^−^	169.0142	2.34
Ethyl gallate	[M − H]^−^	C_9_H_9_O_5_^−^	197.0455	8.89
Fraxetin	[M − H]^−^	C_10_H_7_O_5_^−^	207.0299	8.15
Palmiticacid	[M − H]^−^	C_16_H_31_O_2_^−^	255.2330	20.25
Kaempferol	[M − H]^−^	C_15_H_9_O_6_^−^	285.0405	9.3
Eriodictyol	[M − H]^−^	C_15_H_11_O_6_^−^	287.0561	11.37
Cianidanol	[M − H]^−^	C_15_H_13_O_6_^−^	289.0718	6.95
Bergenin	[M − H]^−^	C_14_H_15_O_9_^−^	327.0722	6.42
Cynaroside	[M − H]^−^	C_21_H_19_O_11_^−^	447.0933	8.98
Astilbin	[M − H]^−^	C_21_H_21_O_11_^−^	449.1089	9.24
Epigallocatechingallate	[M − H]^−^	C_22_H_17_O_11_^−^	457.0776	7.86
Myricitrin	[M − H]^−^	C_21_H_19_O_12_^−^	463.0882	8.8
Nictoflorin	[M − H]^−^	C_27_H_29_O_15_^−^	593.1512	9.09
Rutin	[M − H]^−^	C_27_H_29_O_16_^−^	609.1461	8.66

## Data Availability

The data presented in this study are available in the article and Supplementary Material.

## Supplementary Materials

The supplementary materials for this study can be downloaded at: https://www.mdpi.com/article/10.3390/molecules29040770/s1, Compositional enrichment results for different sites of AJH.

## Abbreviations

AJH: Ardisiae Japonicae Herba, ALI: acute lung injury, PE: petroleum ether site, EA: ethyl acetate site, NB: *n*-butanol site, WS: water site, WBC: white blood cells, NEU%: neutrophil percentage, MONO%: monocyte percentage, LYM%: lymphocyte percentage, EF50: mid-expiration, PAU: the rats’ apnea, PEFb: the rats’ maximum expiratory flow rate, Penh: airway narrowing index, NEU%: neutrophil ratio, LYM%: lymphocyte ratio, DMSO: dimethyl sulfoxide, ESI: electrospray ionization, TIC: total ion current, LPS: lipopolysaccharide, BALF: bronchoalveolar lavage fluid.
