# Supplementary material for: Discovery of the Active Compounds of the Ethyl Acetate Extract Site of Ardisia japonica (Thunb.) Blume for the Treatment of Acute Lung Injury
Source: Molecules. 2024 Feb 7;29(4):770. doi: 10.3390/molecules29040770 (PMC10891587; doi:10.3390/molecules29040770)
Supplement: Supplementary file 1 [file molecules-29-00770-s001.zip › molecules-2825828-supplementary.pdf]

PE site			Compound categoi	Name	formular	Ion mode	RT	Response	Theoretical value	Measured value	MS/MS
1	1	5	phenylpropanoid	(E)-p-coumaric acid	C9 H8 O3	+	7.52	4.51E+06	165.0546	165.0538	147.04334,119.04849,93.03310
2	2	10	phenylpropanoid	Esculetin	C9 H6 O4	+	6.47	1.73E+06	179.0339	179.0329	151.03815,135.04334
3	3	11	phenylpropanoid	IN00458	C9 H6 O3	+	8.36	1.63E+06	163.0390	163.0381	135.04334,105.03292,77.03817
4	4	12	phenylpropanoid	Umbelliferone	C9 H6 O3	+	12.89	3.58E+07	163.0390	163.0381	135.04327
5	5	13	phenylpropanoid	Coumarin	C9 H6 O2	+	9.85	1.10E+07	147.0441	147.0432	119.04853,103.05367
6	6	75	phenylpropanoid	3-Phenyl-1-propanol	C9 H12 O	+	9.07	2.73E+07	137.0961	137.0954	119.08493,93.06943,91.05378,79.05384,77.03822
7	7	99	phenylpropanoid	3-Phenylpropanoic acid	C9 H10 O2	+	8.85	5.01E+06	151.0754	151.0746	133.06416,105.06929,84.95926,79.05381,77.03818,57.03319,55.01753
8	8	2205	phenylpropanoid	cinnamyl tiglate	C14 H16 O2	+	10.48	2.75E+06	217.1223	217.1214	135.0797,117.0693,105.0693,91.0538,79.0538
9	9	2379	phenylpropanoid	CHEMBRDG-BB 6484841	C13 H14 O2	+	10.28	6.97E+06	203.1067	203.1059	175.0744,172.0873,161.0589,145.0641,117.0693,91.0538
10	10	2391	phenylpropanoid	Bergapten	C12 H8 O4	+	9.71	7.06E+07	217.0495	217.0487	173.0588,171.0434,147.0433
11	11	2496	phenylpropanoid	Asarone	C12 H16 O3	+	10.60	6.39E+07	209.1172	209.1163	194.0928,163.0745,151.0745,139.0746,121.0641,109.0643,95.0487,79.0539
12	12	2552	phenylpropanoid	6,7,8-trimethoxy-2H-chromen-2-one	C12 H12 O5	+	7.97	1.76E+07	237.0758	237.0747	205.0489,162.0615,162.0303,137.0589,107.0485
13	13	2719	phenylpropanoid	4-methoxy-6-(prop-2-en-1-yl)-2H-1,3-benzodioxole	C11 H12 O3	+	20.52	9.32E+06	193.0859	193.0850	161.0589,135.0435
14	14	2728	phenylpropanoid	Ethyl cinnamate	C11 H12 O2	+	6.42	3.52E+06	177.0910	177.0900	149.0589,103.0537,91.0537,77.0382
15	15	2749	phenylpropanoid	Scoparone	C11 H10 O4	+	7.37	1.83E+07	207.0652	207.0644	179.0693,163.0746,148.0511,119.0485
16	16	2755	phenylpropanoid	Fraxetin	C10 H8 O5	+	4.62	1.79E+07	209.0444	209.0433	191.0331,177.0173,165.0537,149.0589,153.0538,139.0383,109.0278
17	17	2761	phenylpropanoid	Scopoletin	C10 H8 O4	+	9.36	3.67E+06	193.0495	193.0486	165.1365,150.0303,135.0435,123.0591,107.0486,95.0487
18	18	2763	phenylpropanoid	Herniarin	C10 H8 O3	+	9.84	1.80E+07	177.0546	177.0537	149.0589,145.0277,131.0485,103.0537,77.0381
19	19	2887	phenylpropanoid	Isoeugenol	C10 H12 O2	+	14.99	9.72E+06	165.091	165.0901	147.0797,137.0590,135.0797,123.0433,107.0486,93.0329,77.0382
20	20	2906	phenylpropanoid	(E)-2-Methoxycinnamaldehyde	C10 H10 O2	+	22.11	3.33E+06	163.0754	163.0744	135.0435,107.0485,105.0328,790538,77.0382
21	21	1507	phenylpropanoid	1256801	C17 H20 O4	+	11.90	3.56E+06	289.1434	289.1420	145.0640,177.1264,133.0641
22	22	1975	phenylpropanoid	Hexyl cinnamaldehyde	C15 H20 O	+	11.44	4.20E+07	217.1587	217.1578	175.1108,147.0797,133.1005,105.0693,91.0538
23	23	2388	phenylpropanoid	AKOS BC-2979	C13 H12 O3	+	10.83	1.21E+07	217.0859	217.0850	175.0380,173.0952,147.0433
24	1	43	terpene	Isophorone	C9 H14 O	+	9.46	3.21E+06	139.1117	139.1110	97.10066,97.06427,83.04871,81.06947,67.05386,55.05393
25	2	280	terpene	(3beta)-28-Hydroxylup-20(29)-en-3-yl (2E)-3-(4-hydr	C39 H56 O4	+	19.60	1.01E+08	589.4251	589.4253	121.0638,109.1005,81.0694,79.0538,69.0696,67.0539,55.0540
26	3	293	terpene	Euphorbiaproliferin F	C36 H44 O11	+	24.44	6.89E+07	653.2956	653.2946	93.0694,69.0696,67.0538,55.0540,53.0378
27	4	319	terpene	3-Acetoxyurs-12-en-23-oic acid	C32 H50 O4	+	18.70	1.19E+07	499.3782	499.3755	123.1162,109.1006,95.0850,83.0851,69.0695,67.0539,57.0696,55.0540
28	5	352	terpene	Betulin	C30 H50 O2	+	23.71	2.00E+07	443.3884	443.3857	121.1006,109.1006,81.0695,69.0695,67.0539
29	6	355	terpene	1916451	C30 H50 O	+	19.81	3.43E+06	427.3934	427.3914	111.1163,109.1007,105.0694,81.0695,69.0696,67.0539
30	7	360	terpene	Asiatic acid	C30 H48 O5	+	12.25	2.27E+06	489.3575	489.3553	139.0747,97.1007,95.0850,89.0593,85.1008,83.0851,69.0696,67.0539,57.0696,55.0539
31	8	585	terpene	NP-009800	C23 H36 O4	+	12.36	1.79E+07	399.2686	399.2484	109.1006,93.0694,81.0695,79.0538,70.0648,67.0539,55.0539
32	9	767	terpene	(4aS,5R,6S,8aS)-5-[(3E)-5-methoxy-3-methyl-5-oxope	C21 H32 O4	+	17.47	2.98E+08	349.2373	331.2225	149.0954,95.0850,8.0851,81.0694,69.0695,67.0539,57.0696,55.0539
33	10	773	terpene	NP-006817	C21 H32 O3	+	17.17	9.95E+07	333.2424	333.2411	83.0851,81.0694,69.0695,57.0696,55.0539
34	11	865	terpene	Lagochilin	C20 H36 O5	+	14.20	1.15E+66	357.2636	357.2617	117.0540,105.0693,97.1007,81.0695,67.0539,57.0696,57.0332,55.0540
35	12	1181	terpene	7-Hydroxy-4a,6a,7-trimethyltetradecahydroindeno[4,5-	C19 H30 O3	+	16.35	1.11E+07	307.2268	307.2254	139.0746,123.1162,97.1007,95.0850,69.0695,55.0540
36	13	1648	terpene	1-(5,5,8,8-Tetramethyl-5,6,7,8-tetrahydro-2-naphthale	C16 H22 O	+	10.48	1.46E+07	231.1743	231.1733	175.0643,133.0643,105.0330
37	14	1715	terpene	NP-004917	C15 H26 O3	+	14.68	2.53E+07	255.1955	255.1942	219.1734,97.1006,83.0851,71.0488,69.0695,55.0539,55.0176
38	15	1743	terpene	(2E,6E)-Farnesol	C15 H26 O	+	15.72	1.36E+06	223.2056	223.2047	187.1472,85.0644,
39	16	2002	terpene	DEHYDROCOSTUS LACTONE	C15 H18 O2	+	10.80	3.15E+07	231.1380	231.1369	175.0747,159.1159,131.0849
40	17	2337	terpene	2-[(1E)-1-Buten-3-yn-1-yl]-1,3,3-trimethylcyclohexen	C13 H18	+	11.45	3.71E+07	175.1481	175.1472	175.1472
41	18	2791	terpene	Isomenthone DI	C10 H18 O	+	12.04	6.47E+06	155.1430	155.1422	111.0799,83.0487,69.0332
42	19	2807	terpene	Pulegone	C10 H16 O	+	8.46	3.96E+06	153.12739	153.1266	109.0642,95.0850,81.0694,69.0695
43	20	2816	terpene	2-bornene	C10 H16	+	18.02	2.78E+06	137.1325	137.1319	137.1319
44	21	2657	terpene	1-carboxy-3-hydroxyadamantane	C11 H16 O3	+	7.06	7.34E+07	197.1172	197.1163	179.1057,161.0952,151.1110,133.1005,79.0538,77.0382
45	22	2222	terpene	3748	C13 H24 O3	+	9.90	2.02E+05	229.1798	229.1786	123.1162,71.0488
46	23	2171	terpene	1,1,4,4-Tetramethyltetralin	C14 H20	+	14.50	1.87E+07	189.1638	189.1630	147.1160,133.1004,77.0382
47	24	1823	terpene	(-)-Caryophyllene oxide	C15 H24 O	+	11.98	3E+08	221.18999	221.1891	175.1472,161.1316,147.1160,123.1162,121.1005,109.1006
48	25	1901	terpene	9-hydroxy-2,10,10-trimethyltricyclo[6.3.0.0Âa,âµ]undec	C15 H22 O3	+	12.62	2.57E+07	251.1642	251.1635	205.1578,163.1473,137.1317,109.1006,95.0850
49	26	530	terpene	4-{[5-(7-hydroxy-5,5,8a-trimethyl-2-methylidene-deca	C24 H40 O5	+	19.16	2.36E+07	409.2949	409.2932	181.0850,169.0850,154.0617,109.1006,95.0851,83.0851,81.0695,69.0695,57.0696,55.0539
50	27	291	terpene	(3beta)-3-[(3,3-Dimethylbutanoyl)oxy]urs-12-en-28-o	C36 H58 O4	+	17.63	1.24E+06	555.4408	555.4383	555.4383
51	28	255	terpene	Lup-20(29)-ene-3,28-diyl (2E,2'E)bis(3-phenylacrylate)	C48 H62 O4	+	18.62	3.89E+07	703.4721	703.4714	81.0695,79.0539,69.0695,67.0539,55.0539
52	29	1472	terpene	NP-019547	C17 H26 O5	+	11.43	2.20E+06	311.1853	311.1839	149.0952,119.0848,107.0848,95.0850,93.0694,81.0695
53	30	312	terpene	citranaxanthin	C33 H44 O	+	19.76	7.80E+07	457.3465	457.3472	121.1006,109.1006,107.0850,95.0850,81.0695
54	31	2294	terpene	2595	C13 H20 O	+	9.01	1.12E+07	193.1587	193.1577	165.1266,151.1109,109.0642,83.0487,69.0331
55	32	2441	terpene	3,5-Dimethyladamantan-1-ol	C12 H20 O	+	12.64	4.10E+06	181.1587	181.1577	153.1265,125.0954,57.0332
56	33	407	terpene	ganoderal A	C30 H44 O2	+	16.67	5.39E+06	437.3414	437.3392	123.1161,109.1006,95.0850,83.0851,81.0695,71.0851,69.0695,67.0539,57.0696,55.0539
57	1	386	steroid	NP-005821	C30 H46 O3	+	19.73	2.78E+07	455.3520	455.3495	135.1162,123.1162,107.0850,89.0593,81.0695,69.0695,67.0539,55.0540
58	2	420	steroid	Sitostenone	C29 H48 O	+	19.76	1.87E+08	413.3778	413.3757	123.0798,109.0643,95.0851,83.0488,81.0695
59	3	442	steroid	(2beta,3beta,5beta,16beta,22R,24xi)-2,3,14,16,20,22-He	C28 H46 O7	+	20.74	1.42E+07	495.3316	495.3296	89.0593,85.1008,71.0852,57.0696
60	4	443	steroid	(2β,3β,9ξ,17ξ,22R)-2,3,14,20,22-Pentahydroxyergost-7-	C28 H46 O6	+	12.77	5.68E+06	479.3367	479.3346	109.1006,97.0279,95.0850,85.0280,81.0695,69.0331,67.0539
61	5	452	steroid	(3alpha,5alpha)-21-(4-Methylphenyl)pregn-20-yn-3-c	C28 H38 O	+	20.05	2.03E+06	391.2995	391.2979	157.1003,23.1160,105.0693,97.1007,93.0694,91.0538,79.0538,
62	6	458	steroid	(3beta,5alpha,6alpha,15alpha,16beta,24R,25R)-24,27-C	C27 H46 O6	+	22.02	7.26E+06	467.3367	467.3383	121.1006,109.1006,107.0850,81.0695,69.0696
63	7	464	steroid	Cholest-4-en-3-one	C27 H44 O	+	24.47	5.93E+06	385.3465	385.3449	123.0798,109.0642,95.0850,85.1007,83.0487,81.0695,71.0852,57.0696,55.050
64	8	531	steroid	3a,7a-Dihydroxycholanoic acid	C24 H40 O4	+	15.93	3.22E+08	393.2999	393.3027	109.1006,97.0643,95.0850,83.0487,69.0695,67.0539,57.0696,55.0540
65	9	554	steroid	(17beta)-3-Methoxy-17-(tetrahydro-2H-pyran-2-yloxy	C24 H34 O3	+	16.99	1.64E+06	371.2581	371.2568	109.0592,83.0488,71.0852,59.0489,57.0696,55.0540
66	10	812	steroid	Norgestrel	C21 H28 O2	+	13.18	6.64E+07	313.2162	313.2197	159.1160,147.1160,119.0849,105.0693,95.0851,93.0694,81.0695,79.0539,67.0539
67	11	1113	steroid	Exemestane	C20 H24 O2	+	15.13	1.38E+06	297.1849	297.1835	131.0849,125.0955,121.1006,119.0849,97.1007,83.0487,81.0695,67.0539,55.0540
68	12	1215	steroid	Boldione	C19 H24 O2	+	14.90	1.20E+07	285.1849	285.1839	187.1112,149.1316,
69	13	1387	steroid	Estriol	C18 H24 O3	+	11.80	1.43E+07	289.1798	289.1765	173.0952,147.0796,109.0642,107.0485,97.0643,95.0850,81.0694,55.0539
70	14	1401	steroid	19-Norandrostenedione	C18 H24 O2	+	11.49	3.35E+07	273.1849	273.1835	175.1108,173.1316,107.0850,95.0850,93.0694,81.0695,79.0539,55.0539
71	15	1413	steroid	2-Hydroxyestrone	C18 H22 O3	+	12.22	7.71E+05	287.1642	287.1629	193.1213,189.0901,123.0434
72	16	1420	steroid	3-deoxyestrone	C18 H22 O	+	11.54	1.55E+07	255.1743	255.1733	161.1316,159.1160,131.0849,105.0694
73	1	487	flavonoid	Nictoflorin	C27 H30 O15	+	8.08	4.39E+05	595.1657	595.1627	95.0487,89.0592,57.0332
74	2	472	flavonoid	Phloretin	C15 H14 O5	-	11.22	1.50E+06	273.0768	273.0740	273.0740
75	3	490	flavonoid	Genistein	C15 H10 O5	-	6.22	5.11E+04	269.0455	269.0456	269.0456
76	4	157	flavonoid	5-(5,7-Dihydroxy-3-methoxy-4-oxo-4H-chromen-2-y	C21 H20 O11	-	8.06	3.62E+07	447.0933	447.0917	447.0917
77	1	658	quinone	2-Hydroxy-5-methoxy-3-(10-pentadecenyl)benzo-1,4	C22 H34 O4	+	17.36	6.51E+08	363.2530	363.2514	179.0693,165.0537,153.0538,125.0590,123.0434,107.0486
78	2	1479	quinone	Embelin	C17 H26 O4	+	14.26	1.71E+07	295.1904	295.1863	295.1863,195.1370167.1421,155.0332,
79	3	2146	quinone	2,6-Di-tert-butyl-1,4-benzoquinone	C14 H20 O2	+	11.49	3.37E+07	221.1536	221.1528	193.158
80	4	288	quinone	Idebenone	C19 H30 O5	-	14.91	1.98E+08	337.2020	337.1986	337.1986
81	5	289	quinone	Rapanone	C19 H30 O4	-	16.05	6.88E+06	321.2071	321.2076	321.2076
82	1	17	phenolic acids, cart	2,4,6,8,10,12,14,16-Octaoxaheptadecane	C9 H20 O8	+	2.09	3.03E+05	257.1231	257.1228	75.04367
83	2	20	phenolic acids, cart	9-Oxononanoic acid	C9 H16 O3	+	11.75	4.86E+06	173.1172	173.1165	155.10584,85.06443,83.08513,71.04880,69.06953,57.03323,55.05394
84	3	30	phenolic acids, cart	Ethyl 2-oxocyclohexanecarboxylate	C9 H14 O3	+	13.87	3.52E+06	171.1016	171.1016	111.07986,93.06942,81.06949,71.04881,57.03324,55.01757,55.05395
85	4	36	phenolic acids, cart	RI2735000	C9 H14 O2	+	11.76	7.57E+06	155.1067	155.1059	99.04375,95.08505,85.02786
86	5	50	phenolic acids, cart	1,2,3,4-Tetramethyl-1,3-cyclopentadiene	C9 H14	+	13.84	9.20E+06	123.1168	123.1162	95.08504,81.06946,67.05389,55.05395
87	6	63	phenolic acids, cart	DC2810000	C9 H12 O3	+	7.71	5.57E+06	169.0859	169.0851	137.05902
88	7	69	phenolic acids, cart	4-Ethylguaiacol	C9 H12 O2	+	10.56	7.13E+06	153.0910	153.0903	135.07968,125.05910
89	8	78	phenolic acids, cart	1,8-NONADIYNE	C9 H12	+	14.65	7.81E+06	121.1012	121.1005	67.05388,53.03828
90	9	80	phenolic acids, cart	Cumene	C9 H12	+	11.96	5.86E+06	121.1012	121.1005	79.05381
91	10	85	phenolic acids, cart	3,4-Dimethoxybenzoic acid	C9 H10 O4	+	17.97	7.75E+06	183.0652	183.0652	165.05376,137.05896
92	11	89	phenolic acids, cart	3,5-Dimethoxybenzoic acid	C9 H10 O4	+	6.25	4.32E+06	183.0652	183.0643	165.05371,151.03825,137.05907
93	12	94	phenolic acids, cart	Apocynin	C9 H10 O3	+	9.26	3.48E+07	167.0703	167.0694	149.05891
94	13	111	phenolic acids, cart	2-Anisic acid	C8 H8 O3	+	6.14	1.86E+07	153.0546	153.0539	135.04341,120.04382,107.04868,105.03298,77.03827
95	14	113	phenolic acids, cart	Vanillin	C8 H8 O3	+	6.14	1.86E+07	153.0546	153.0539	135.04341,125.05911,121.02781
96	15	115	phenolic acids, cart	Methyl salicylate	C8 H8 O3	+	18.04	3.90E+06	153.0546	153.0539	135.04332,123.04349,107.04865,93.03297
97	16	121	phenolic acids, cart	4-Methoxybenzaldehyde	C8 H8 O2	+	15.69	6.36E+07	137.0597	137.0590	109.06413
98	17	123	phenolic acids, cart	2145	C8 H8 O2	+	18.45	3.67E+07	137.0597	137.0590	91.05382
99	18	124	phenolic acids, cart	2-Methoxybenzaldehyde	C8 H8 O2	+	9.18	1.00E+07	137.0597	137.0590	121.06416,109.06414,107.04855,91.05373,77.03818
100	19	128	phenolic acids, cart	Acetophenone	C8 H8 O	+	10.15	2.27E+06	121.0648	121.0642	105.06931,77.03820
101	20	134	phenolic acids, cart	ortho-Phthalaldehyde	C8 H6 O2	+	6.85	5.46E+07	135.0441	135.0434	107.04856,91.05383
102	21	135	phenolic acids, cart	2-Hydroxybicyclo[4.2.0]octa-1,3,5-triene-7,8-dione	C8 H4 O3	+	12.93	2.74E+08	149.0233	149.0225	121.02777,93.03300
103	22	137	phenolic acids, cart	Bicyclo(4.2.0)octa-1,3,5-triene-7,8-dione	C8 H4 O2	+	8.36	1.84E+06	133.0284	133.0277	105.03297,77.03820
104	23	142	phenolic acids, cart	n-butyl methacrylate	C8 H14 O2	+	10.32	1.46E+06	143.1067	143.1058	125.09525,115.07465,85.06433,73.06447,71.04878,69.03313,57.06963,55.05394
105	24	153	phenolic acids, cart	NP-019360	C8 H12 O4	+	24.08	3.10E+06	173.0808	173.0801	145.04875,113.05913,111.07987,109.06441,95.04854,69.03307,67.05388
106	25	160	phenolic acids, cart	Ethyl sorbate	C8 H12 O2	+	7.47	2.73E+06	141.0910	141.0603	123.07976,113.05907,99.07992,95.04867,81.06946,67.05387,57.03321,55.05394
107	26	161	phenolic acids, cart	Dimedone	C8 H12 O2	+	13.65	1.22E+06	141.0910	141.0903	123.07976,99.07992,81.06946,57.03321,55.05394
108	27	162	phenolic acids, cart	GV9100000	C8 H12 O	+	10.57	5.83E+06	125.0961	125.0954	81.03307,51.02266,107.08492,69.03317
109	28	170	phenolic acids, cart	Penicillic acid	C8 H10 O4	+	5.03	2.36E+06	171.0652	171.0642	153.05366,139.03812,125.05901,101.02256,95.04858,69.03311
110	29	175	phenolic acids, cart	Veratrole	C8 H10 O2	+	7.09	1.49E+06	139.0754	139.0746	107.04851,77.03816
111	30	176	phenolic acids, cart	Guaethol	C8 H10 O2	+	9.69	6.33E+06	139.0754	139.0746	121.06414,111.04344,109.02773
112	31	178	phenolic acids, cart	Tyrosol	C8 H10 O2	+	10.24	1.50E+06	139.0754	139.0746	121.06414,95.04864
113	32	183	phenolic acids, cart	2-Methoxyresorcinol	C7 H8 O3	+	4.64	4.50E+06	141.0546	141.0538	123.04339,109.02782
114	33	186	phenolic acids, cart	Guaiacol	C7 H8 O2	+	6.14	6.19E+06	125.0597	125.0590	107.04852,93.03294
115	34	192	phenolic acids, cart	Gallic acid	C7 H6 O5	+	3.05	5.81E+05	171.0288	171.0279	153.01739,125.02268
116	35	196	phenolic acids, cart	gentisaldehyde	C7 H6 O3	+	8.97	1.49E+06	139.0390	139.0384	121.02773,111.04344
117	36	212	phenolic acids, cart	Succinylacetone	C7 H10 O4	+	19.92	1.64E+07	159.0652	159.0643	109.02772,95.04856,81.06935,71.04878,67.05388,57.03323
118	37	214	phenolic acids, cart	2-OXOCYCLOHEXANECARBOXYLICACID	C7 H10 O3	+	22.56	8.34E+06	143.0703	143.0696	129.0540,115.0748,111.0435,97.0643,83.0488,73.0645,67.0539,55.0177,55.0540
119	38	218	phenolic acids, cart	ß,ß-Dimethyl-γ-methylene-γ-butyrolactone	C7 H10 O2	+	11.26	8.15E+05	127.0754	127.0747	81.0695,81.0331,69.0695,69.0332,67.0539,57.0332,55.0540,53.0383
120	39	219	phenolic acids, cart	1-Cyclohexenecarboxylic Acid	C7 H10 O2	+	11.63	1.65E+06	127.0754	127.0747	81.0331,81.0695,69.0332,69.0695,57.0332,55.0540,53.0383
121	40	222	phenolic acids, cart	lactide	C6 H8 O4	+	1.43	4.20E+06	145.0495	145.0487	127.0383,99.0435,85.0279,81.0694,71.0488,69.0331,57.0332,55.0175
122	41	226	phenolic acids, cart	Furaneol	C6 H8 O3	+	21.60	1.17E+07	129.0546	129.0540	101.0592,83.0487,71.0488,69.0695,59.0488,55.0539
123	42	227	phenolic acids, cart	Kojic acid	C6 H6 O4	+	4.34	3.25E+06	143.0339	143.0331	125.0226,97.0279,69.0331,55.0176
124	43	230	phenolic acids, cart	Maltol	C6 H6 O3	+	4.29	1.55E+06	127.0390	127.0383	109.0278,69.0332,53.0383
125	44	232	phenolic acids, cart	Hydroxymethylfurfural	C6 H6 O3	+	1.43	4.19E+06	127.0390	127.0383	109.0279,99.0435,97.0279,81.0331,69.0332,57.0332,55.0176,
126	45	242	phenolic acids, cart	Adipic acid	C6 H10 O4	+	22.57	2.54E+08	147.0652	147.0645	129.0540,111.0435,101.0592,95.0487,83.0487,79.0537,59.0488,55.0539
127	46	274	phenolic acids, cart	L-threonic acid	C4 H8 O5	+	1.45	6.04E+05	137.0444	137.0450	119.0346,109.0502,73.0280,69.0331,57.0331,55.0176
128	47	522	phenolic acids, cart	4,5,6-trimethyl-3-pentadecylcatechol	C24 H42 O2	+	24.60	7.60E+05	363.3258	363.3243	137.0590,85.1008,71.0852,69.0695,57.0696,55.0540
129	48	543	phenolic acids, cart	Bis(2-ethylhexyl) phthalate	C24 H38 O4	+	20.55	1.52E+09	391.2843	391.2829	177.0902,153.0539,149.0591,149.0228,137.0591,123.0435,125.0592,107.0484,57.0696,
130	49	616	phenolic acids, cart	Bis(2-ethylhexyl)adipate	C22 H42 O4	+	22.57	1.79E+09	371.3156	371.3144	147.0644,143.0696,129.0540,111.0435,101.0592,83.0487,71.0852,57.0696,55.0540
131	50	649	phenolic acids, cart	(7E,10E,13E,16E)-7,10,13,16-Docosatetraenoic acid	C22 H36 O2	+	18.43	8.24E+08	333.2788	333.2775	125.0591,83.0851,69.0695,55.0540
132	51	938	phenolic acids, cart	(-)-Prostaglandin E2	C20 H32 O5	+	15.33	8.06E+06	353.2323	353.2309	153.0539,109.1006,107.0850,95.0851,93.0694,81.0695,67.0539
133	52	969	phenolic acids, cart	2K2DJ01BB7	C20 H32 O2	+	15.48	3.53E+07	305.2475	305.2460	109.1006,95.0851,93.0694,81.0695,69.0695,67.0539,55.0540
134	53	1066	phenolic acids, cart	(5E,12E,14E)-11-Oxoprosta-5,9,12,14-tetraen-1-oic ac	C20 H28 O3	+	19.76	1.48E+06	317.2111	317.2096	109.1006,95.0851,81.0695,57.0696,55.0540
135	54	1185	phenolic acids, cart	Tridecanophenone	C19 H30 O	+	14.11	1.37E+06	275.2369	275.2359	147.1160,117.0693,105.0693,91.0538,81.0695,70.0538,67.0539,55.0540
136	55	1191	phenolic acids, cart	ubiquinol 10	C19 H28 O4	+	14.92	4.56E+08	321.2060	321.2063	191.0693,119.0849,95.0850,93.0694,91.0537,81.0694,69.0695,57.0696,55.0539
137	56	1229	phenolic acids, cart	Nonaethylene Glycol	C18 H38 O10	+	5.91	6.14E+07	415.2538	415.2518	175.0955,117.0903,73.0644,
138	57	1258	phenolic acids, cart	(+/-)12(13)-DiHOME	C18 H34 O4	+	19.39	2.38E+08	315.2530	315.2530	205.1576
139	58	1407	phenolic acids, cart	octyl-2-naphthol	C18 H24 O	+	14.44	1.66E+07	257.1900	257.1889	187.1108,169.1003,109.1006,95.0850,83.0851,81.0695,69.0695,67.0539,5.0696,55.0540
140	59	1421	phenolic acids, cart	2,3-Dimethyl-2,3-diphenylbutane	C18 H22	+	13.06	1.76E+06	239.1794	239.1784	161.1317,119.0849,79.0538
141	60	1501	phenolic acids, cart	(1E,4E)-1,4-Undecadien-1-ylbenzene	C17 H24	+	13.05	2.87E+07	229.1951	229.1939	91.0538,79.0539
142	61	1518	phenolic acids, cart	1-Ethoxy-1,3-dimethoxydodecane	C16 H34 O3	+	16.35	9.69E+06	275.2581	275.2567	89.0593,85.1007,81.0695,71.0852,69.0695,67.0539,57.0696,55.0540
143	62	1532	phenolic acids, cart	Ethyl myristate	C16 H32 O2	+	2.09	7.93E+05	257.2475	257.2465	103.0748,95.0850,89.0592,81.0695,71.0488,71.0852,69.0695,57.0696,55.0539
144	63	1544	phenolic acids, cart	Caprylic anhydride	C16 H30 O3	+	13.28	6.59E+06	271.2268	271.2255	253.2148,155.0694,109.1006
145	64	1551	phenolic acids, cart	Palmitoleic acid	C16 H30 O2	+	17.11	1.14E+07	255.2319	255.2306	135.1161,121.1005,97.1007,95.0850,93.0694,83.0851,81.0695
146	65	1631	phenolic acids, cart	Decanophenone	C16 H24 O	+	14.17	1.27E+07	233.1900	233.1889	159.1159,95.0486,117.0692,91.0537
147	66	1633	phenolic acids, cart	4-N-OCTYLSTYRENE	C16 H24	+	11.28	5.40E+06	217.1951	217.1942	189.1628,77.0382,50.5850
148	67	1650	phenolic acids, cart	1-Ethynyl-4-octylbenzene	C16 H22	+	14.12	4.36E+06	215.1794	215.1785	185.1315,145.1004,103.0536,77.0382
149	68	1662	phenolic acids, cart	NSC 1775	C16 H18 O2	+	10.65	7.69E+05	243.1380	243.1374	149.0953,107.0485,93.0330,79.0538
150	69	1690	phenolic acids, cart	14-Pentadecenoicacid	C15 H28 O2	+	15.67	1.57E+06	241.2162	241.2150	171.1159
151	70	1789	phenolic acids, cart	6-(7-methyloctyl)-1H,3H,4H,6H-furo[3,4-c]furan-1-on	C15 H24 O3	+	11.54	2.91E+07	253.1798	253.1787	197.1163,141.0540,123.0435
152	71	1840	phenolic acids, cart	Nonylbenzene	C15 H24	+	14.62	2.82E+07	205.1951	205.1943	135.1161,121.1005,93.0694,79.0538
153	72	1902	phenolic acids, cart	3,5-ditert-butyl-4-hydroxybenzaldehyde	C15 H22 O2	+	14.48	5.80E+07	235.1693	235.1682	207.1734,179.1057,161.0952,133.1005,77.0382
154	73	1903	phenolic acids, cart	3,5-Di-tert-butylbenzaldehyde	C15 H22 O	+	13.14	9.20E+07	219.1743	219.1734	191.1785,163.1109,133.1005,79.0538,77.0382
155	74	1905	phenolic acids, cart	S-Curcumene	C15 H22	+	11.98	3.52E+08	203.1794	203.1786	147.1160,133.1005,121.1005,105.0693,93.0694,77.0382
156	75	1966	phenolic acids, cart	Turmerone	C15 H20 O	+	14.48	3.13E+07	217.15869	217.1577	175.1108,161.0952,133.1004,105.0693,91.0537
157	76	1986	phenolic acids, cart	4-Heptylphenylacetylene	C15 H20	+	10.84	4.12E+07	201.1638	201.1631	159.1160,145.1004,119.0849,117.0693,91.0538,79.0539
158	77	2006	phenolic acids, cart	1,2,3,4,5,6,7,8-Octahydro-9-phenanthrenecarbaldehyd	C15 H18 O	+	11.99	1.89E+07	215.1430	215.1421	186.1030,173.0952,159.0796
159	78	2012	phenolic acids, cart	1-[(E)-2-(2-Cyclohexen-1-yl)vinyl]-3-methylbenzene	C15 H18	+	14.49	1.19E+07	199.1481	199.1473	157.1003,133.1005,131.0848,119.0849,105.0693,91.0537,77.0382
160	79	2023	phenolic acids, cart	Dibenzylmethane	C15 H16	+	**12.32**	**3.07E+06**	**197.1325**	**197.1317**	19.0848,105.0693,91.0537,77.0381
161	80	2062	phenolic acids, cart	3-Oxotetradecanoic acid	C14 H26 O3	+	12.12	4.95E+06	243.1955	243.1943	85.0644,57.0696
162	81	2088	phenolic acids, cart	4'-ethyl-1,1'-bi(cyclohexyl)-4-one	C14 H24 O	+	14.48	3.96E+06	209.1900	209.1891	191.1784,165.1263,163.1472,111.1162
163	82	2101	phenolic acids, cart	(+/-)-C75	C14 H22 O4	+	10.70	7.42E+06	255.1591	255.1582	181.1214,123.1162
164	83	2121	phenolic acids, cart	4-Octylphenol	C14 H22 O	+	12.37	6.39E+06	207.1743	207.1735	189.1628,121.0642,91.0538,77.0382
165	84	2128	phenolic acids, cart	Octylbenzene	C14 H22	+	**14.47**	**3.37E+06**	**191.1794**	**191.1785**	135.1161,93.0694,79.0538
166	85	2140	phenolic acids, cart	NP-020240	C14 H20 O3	+	11.83	8.18E+06	237.1485	237.1476	163.1109,135.1162,121.1006,95.0851
167	86	2161	phenolic acids, cart	Octanophenone	C14 H20 O	+	11.72	1.02E+07	205.1587	205.1578	107.0486
168	87	2187	phenolic acids, cart	2-Pentyl-1-indanone	C14 H18 O	+	12.35	1.01E+07	203.1430	203.1423	175.1472,161.1316,147.1160,133.1005,119.0849,105.0693,79.0538

169	88	2192	phenolic acids, carb	1-Ethynyl-4-hexylbenzene	C14 H18	+	11.70	8.61E+06	187.1481	187.1472	145.1005,119.0849,91.0538,79.0539
170	89	2215	phenolic acids, carb	4-Methylbenzophenone	C14 H12 O	+	11.65	2.47E+06	197.0961	197.0953	166.0767,105.0330,77.0381
171	90	2224	phenolic acids, carb	YQ2978400	C13 H24 O2	+	9.24	1.46E+06	213.1849	213.1841	153.1267,69.0696
172	91	2244	phenolic acids, carb	3408	C13 H22 O3	+	12.85	1.01E+07	227.1642	227.1632	181.1213,153.1266,83.0851,57.0696
173	92	2246	phenolic acids, carb	12-Tridecynoic acid	C13 H22 O2	+	10.20	1.35E+07	211.1693	211.1683	123.1162,109.1006,95.0850,81.0695,67.0539
174	93	2250	phenolic acids, carb	OB1804500	C13 H22 O	+	10.81	1.47E+06	195.1743	195.1734	111.0799,83.0852,69.0696
175	94	2288	phenolic acids, carb	645341	C13 H20 O3	+	8.79	6.34E+06	225.1485	225.1475	167.1058,149.0954,123.0799,109.0643
176	95	2293	phenolic acids, carb	Spherophorol	C13 H20 O2	+	12.52	4.21E+07	209.1536	209.1527	137.0589,135.0797,125.0590,111.0433,107.0485,95.0486
177	96	2302	phenolic acids, carb	Heptylbenzene	C13 H20	+	19.39	1.46E+07	177.1638	177.1628	135.1162,121.1005,93.0694,79.0538
178	97	2322	phenolic acids, carb	p-Hexyloxybenzoic Acid	C13 H18 O3	+	11.29	5.04E+07	223.1329	223.1319	177.1629,109.0642,95.0487,77.0381
179	98	2326	phenolic acids, carb	Heptanophenone	C13 H18 O	+	8.89	1.05E+08	191.1430	191.1421	149.0953,135.0797,117.0692,91.0537
180	99	2360	phenolic acids, carb	1-[4-hydroxy-3-(3-methylbut-2-en-1-yl)phenyl]ethan	C13 H16 O2	+	10.41	2.37E+07	205.1223	205.1215	173.0951,145.1004,91.0538
181	100	2366	phenolic acids, carb	Cyclohexyl phenyl ketone	C13 H16 O	+	9.50	1.44E+07	189.1274	189.1264	111.0798,105.0329,83.0851,79.0538
182	101	2373	phenolic acids, carb	4-Pentylphenylacetylene	C13 H16	+	11.57	1.24E+07	173.1325	173.1315	157.1003,143.0848,117.0693,91.0538,77.0382
183	102	2382	phenolic acids, carb	4-hex-1-ynylBenzaldehyde	C13 H14 O	+	10.56	1.17E+06	187.1117	187.1108	159.1160,103.0538,91.0538,77.0381
184	103	2407	phenolic acids, carb	NP-004036	C12 H22 O4	+	10.56	6.52E+06	231.1591	231.1582	117.0693,97.1007
185	104	2438	phenolic acids, carb	acetic acid geranyl ester	C12 H20 O2	+	10.75	6.92E+06	197.1536	197.1527	179.1058,125.0591,111.0435
186	105	2451	phenolic acids, carb	3-(3-methoxypropoxy)-4-methoxyphenylmethanol	C12 H18 O4	+	10.70	3.79E+06	227.1278	227.1267	135.0799,121.0643,109.0643,79.0539,77.0383
187	106	2459	phenolic acids, carb	Sedanolide	C12 H18 O2	+	12.02	4.27E+07	195.1380	195.1373	125.0590,111.0434,71.0852
188	107	2476	phenolic acids, carb	Propofol	C12 H18 O	+	13.64	8.25E+07	179.1430	179.1421	161.1318,147.1160,133.1004,119.0849
189	108	2481	phenolic acids, carb	1-t-butyl-4-ethylbenzene	C12 H18	+	11.45	1.06E+07	163.1481	163.1472	135.1161,57.0696,55.0539
190	109	2497	phenolic acids, carb	6-Phenylhexanoic acid	C12 H16 O2	+	14.07	1.27E+08	193.1223	193.1217	165.1265,147.1160,133.1005,119.0849,105.0693,91.0537,77.0382
191	110	2504	phenolic acids, carb	4'-tert-Butylacetophenone	C12 H16 O	+	10.70	1.14E+07	177.1274	177.1267	133.1004,119.0849,91.0538
192	111	2511	phenolic acids, carb	CZ1330000	C12 H16	+	11.45	2.15E+07	161.1325	161.1316	159.1161,145.1006,119.0849,105.0693,91.0538
193	112	2544	phenolic acids, carb	Butylphthalide	C12 H14 O2	+	8.88	8.63E+06	191.1067	191.1058	145.1004,91.0537
194	113	2545	phenolic acids, carb	4-Phenylcyclohexanone	C12 H14 O	+	9.86	4.33E+06	175.1117	175.1109	147.1160,133.1004,119.0849,91.0537
195	114	2572	phenolic acids, carb	(3S,4R)-3-(1-hydroxyhexyl)-4-(hydroxymethyl)oxolan-2	C11 H20 O4	+	10.56	1.91E+07	217.1434	217.1425	181.1214,155.0697,111.0435,
196	115	2605	phenolic acids, carb	Ethyl 3-cyclohexyl-3-oxopropanoate	C11 H18 O3	+	10.24	2.27E+07	199.1329	199.1319	139.0747,111.0797
197	116	2619	phenolic acids, carb	3422	C11 H18 O	+	13.99	2.55E+06	167.1430	167.1424	121.0641,95.0486,67.0538
198	117	2663	phenolic acids, carb	5-Pentylresorcinol	C11 H16 O2	+	9.30	5.20E+08	181.1223	181.1220	163.1108,125.0590,111.0799,107.0486,95.0486
199	118	2669	phenolic acids, carb	SM6750000	C11 H16 O	+	10.81	8.32E+06	165.1274	165.1267	147.1161,91.0538,77.0382
200	119	2684	phenolic acids, carb	Butylparaben	C11 H14 O3	+	9.60	1.07E+08	195.1016	195.1007	177.0901,149.0954,95.0487,79.0538
201	120	2686	phenolic acids, carb	2-Isopropyl-5-methylbenzoic acid	C11 H14 O2	+	14.08	3.96E+07	179.1067	179.1059	161.0952,137.0589,133.1004,105.0693,91.0537,77.0381
202	121	2687	phenolic acids, carb	Butyl benzoate	C11 H14 O2	+	9.00	2.51E+07	179.1067	179.1057	133.1004,105.0693
203	122	2698	phenolic acids, carb	1-Phenyl-2-pentanone	C11 H14 O	+	10.24	1.37E+07	163.1117	163.1109	135.0797,105.0330,77.0381
204	123	2708	phenolic acids, carb	6-METHYLTETRALIN	C11 H14	+	11.44	2.16E+07	147.1168	147.1160	145.1004,119.0849,105.063,91.0537
205	124	2736	phenolic acids, carb	4-Methyl-1-Tetralone	C11 H12 O	+	12.28	4.75E+06	161.0961	161.0952	133.1004,119.0849,91.0537,77.0382
206	125	2773	phenolic acids, carb	Sebacic acid	C10 H18 O4	+	9.69	6.95E+06	203.1278	203.1269	121.1006,95.0851,81.0695,67.0539
207	126	2800	phenolic acids, carb	Ethyl 2-oxocycloheptanecarboxylate	C10 H16 O3	+	10.56	1.44E+07	185.1172	185.1163	139.0746,111.0798,85.0643,57.0696
208	127	2801	phenolic acids, carb	Massoia lactone	C10 H16 O2	+	8.30	2.28E+07	169.1223	169.1215	151.1110,123.1162,69.0696
209	128	2827	phenolic acids, carb	Guaifenesin	C10 H14 O4	+	8.41	2.69E+06	199.0965	199.0957	184.0720,168.0772,153.0538,125.0590,95.0487,79.0538
210	129	2836	phenolic acids, carb	6-Pentyl-2H-pyran-2-one	C10 H14 O2	+	8.84	3.43E+06	167.1067	167.1058	149.0953,138.0542,121.1006,107.0850,95.0487
211	130	2856	phenolic acids, carb	p-cymene	C10 H14	+	9.31	8.45E+06	135.1168	135.1161	93.0694
212	131	2879	phenolic acids, carb	3',4'-Dimethoxyacetophenone	C10 H12 O3	+	9.55	6.25E+07	181.0859	181.0852	135.0433,133.0640,123.0433,95.0486,79.0538
213	132	2880	phenolic acids, carb	4-Phenylbutyric acid	C10 H12 O2	+	14.89	1.00E+07	165.0910	165.0901	137.0954,122.0720,119.0849,107.0486,91.0537,77.0381
214	133	2894	phenolic acids, carb	Butyrophenone	C10 H12 O	+	8.88	4.73E+06	149.10	149.10	105.0694,103.0536,95.0487,91.0538
215	134	2895	phenolic acids, carb	2-ISOPROPENYLTOLUENE	C10 H12	+	9.09	4.57E+07	133.1012	133.1004	131.0848,117.0692,105.0693,91.0537,77.0382

EA site			Compound catec	Name	formular	Ion mode	RT	Response	Theoretical valu	Measured value	MS/MS
1	16	1	phenylpropanoid	4-Coumaric acid	C9 H8 O3	+	5.97	7.66E+06	165.0546	165.0538	147.0433,135.0434,121.0642,119.0486,107.0486,93.0331,77.0382,51.0227
2	18	2	phenylpropanoid	Cinnamic acid	C9 H8 O2	+	9.83	1.52E+06	149.0597	149.0591	103.0537,91.0538,77.0382
3	21	3	phenylpropanoid	trans-Cinnamaldehyde	C9 H8 O	+	7.74	1.93E+06	133.0648	133.0642	119.0184,103.0537,91.0538,77.0382,51.0227
4	25	4	phenylpropanoid	3,5,7-Trihydroxy-4H-chromen-4-one	C9 H6 O5	+	3.66	8.26E+06	195.0288	195.0279	177.0174,153.0175,149.0226,139.0384,123.0434,109.0279,93.0331
5	30	5	phenylpropanoid	5-Hydroxy-1-benzofuran-2-carboxylic acid	C9 H6 O4	+	6.45	5.01E+07	179.0339	179.0330	151.0383,133.0277,123.0435,119.0486,117.0329,107.0486,91.0538
6	32	6	phenylpropanoid	IN00458	C9 H6 O3	+	12.91	4.38E+07	163.0390	163.0381	135.0434,95.0484,94.0409,77.0382,51.0227
7	36	7	phenylpropanoid	Coumarin	C9 H6 O2	+	6.79	1.02E+07	147.0441	147.0433	107.0485,103.0537,95.0486,79.0538,77.0382,51.0227
8	92	8	phenylpropanoid	3-Phenyl-1-propanol	C9 H12 O	+	9.09	2.90E+07	137.0961	137.0954	135.0798,119.0850,105.0694,91.0538,77.0383,51.0227
9	1907	9	phenylpropanoid	Isopentyl cinnamate	C14 H18 O2	+	9.22	9.48E+06	219.1380	219.1371	163.0744,147.0797,103.0537,91.0538,77.0382
10	1931	10	phenylpropanoid	cinnamyl tiglate	C14 H16 O2	+	8.40	9.10E+06	217.1223	217.1215	161.0590,135.0794,117.0694,91.0539,77.0383,83.0488,55.0540
11	1935	11	phenylpropanoid	(2S)-2-(2-hydroxypropan-2-yl)-2H,3H,7H-furo[3,	C14 H14 O4	+	8.65	2.73E+07	247.0965	247.0954	203.1057,185.0952,85.0644,55.0540
12	1946	12	phenylpropanoid	8,8-dimethyl-2H,8H-pyrano[3,2-g]chromen-2-on	C14 H12 O3	+	10.80	2.64E+07	229.0859	229.0851	187.0744,171.0435,67.0539,55.0540
13	2114	13	phenylpropanoid	5,6,7,8-Tetramethoxycoumarin	C13 H14 O6	+	4.62	1.60E+06	267.0863	267.0855	221.0799,193.0486,177.0538,163.0746,153.0539,137.0590
14	2133	14	phenylpropanoid	(6-Oxo-6H-[1,3]dioxolo[4,5-g]chromen-8-yl)aceti	C12 H8 O6	+	3.66	8.99E+06	249.0394	249.0381	217.0123,203.0330,187.0381,179.0329,175.0381,121.0278
15	2134	15	phenylpropanoid	Bergapten	C12 H8 O4	+	9.71	2.71E+07	217.0495	217.0489	189.0537,173.0589,147.0436
16	2254	16	phenylpropanoid	Asarone	C12 H16 O3	+	9.07	3.62E+07	209.1172	209.1164	135.0798,119.0850,109.0643,79.0539
17	2318	17	phenylpropanoid	6,7,8-trimethoxy-2H-chromen-2-one	C12 H12 O5	+	7.97	1.85E+07	237.0758	237.0747	193.0486,176.0459,147.0434,137.0590
18	2338	18	phenylpropanoid	6,7-Dihydroxycoumarin-4-acetic Acid	C11 H8 O6	+	3.66	1.63E+07	237.0394	237.0383	191.0329,177.0537,175.0382,163.0381,123.0434
19	2456	19	phenylpropanoid	NP-014891	C11 H14 O3	+	6.45	7.77E+07	195.1016	195.1006	177.0902,163.0747,151.0747,137.0591,133.0644,57.0332
20	2505	20	phenylpropanoid	Ethyl cinnamate	C11 H12 O2	+	6.45	6.04E+06	177.0910	177.0902	149.0590,131.0486,117.0693,103.0538,91.0538,77.0383
21	2517	21	phenylpropanoid	Scoparone	C11 H10 O4	+	7.35	8.41E+07	207.0652	207.0644	179.0694,175.0380,163.0746,145.0277
22	2528	22	phenylpropanoid	Fraxetin	C10 H8 O5	+	4.59	8.61E+07	209.0444	209.0437	191.0329,181.0486,177.0174
23	2534	23	phenylpropanoid	Scopoletin	C10 H8 O4	+	8.26	5.14E+06	193.0495	193.0486	165.0538,151.0338,149.0590
24	2684	24	phenylpropanoid	Isoeugenol	C10 H12 O2	+	7.00	1.32E+07	165.0910	165.0902	147.0791,137.0591,133.0641,123.0435
25	474	25	phenylpropanoid	Bergenin	C14 H16 O9	−	10.45	5.33E+06	327.0722	327.0708	327.0708
26	2120	26	phenylpropanoid	CHEMBRDG-BB 6484841	C13 H14 O2	+	8.78	7.52E+06	203.1067	203.1059	135.0798,117.0693,91.0538,77.0383,57.0333
27	2330	27	phenylpropanoid	(5,7-Dihydroxy-4-methyl-2-oxo-2H-chromen-3-	C12 H10 O6	+	4.59	1.53E+08	251.0550	251.0539	190.0252,177.0537,175.0381,163.0381
28	2342	28	phenylpropanoid	6-Methoxy-4-oxo-4H-chromene-2-carboxylic ac	C11 H8 O5	+	4.34	1.32E+07	221.0444	221.0436	175.0380,151.0381,123.0432,107.0485
29	468	29	phenylpropanoid	(8S,16E)-4-(3-Isopropoxy-4-methoxyphenyl)-5-m	C32 H38 O8	+	23.79	1.16E+06	551.2639	551.2634	147.1162,135.1162,133.1005,123.1162,121.1006,109.1006,107.0850,95.0851,93.0695,81.0695,57.0696
30	843	30	phenylpropanoid	Ethyl 3-{7-[2-(allyloxy)-2-oxoethoxy]-4,8-dimethy	C21 H24 O7	+	7.41	1.88E+07	389.1595	389.1578	203.1058,188.0824,177.0903,151.0748,137.0592,13.0515,122.0358,107.0487
31	844	31	phenylpropanoid	(3R,4R)-4-[(3,4-dimethoxyphenyl)methyl]-3-[(4-h	C21 H24 O6	+	9.20	3.90E+07	373.1646	373.1578	177.0902,165.0538,151.0747,149.0590,137.0591,121.0643,91.0538
32	1053	32	phenylpropanoid	Matairesinol	C20 H22 O6	+	8.56	5.80E+06	359.1489	359.1474	235.0956,205.0850,189.0902,177.0902,163.0746,151.0746,137.0592,123.0434
33	1797	33	phenylpropanoid	Luvangetin	C15 H14 O4	+	11.31	2.22E+08	259.0965	259.0954	203.0329,191.0331,67.0538,55.0540
34	1897	34	phenylpropanoid	Lily aldehyde	C14 H20 O	+	8.85	2.46E+06	205.1587	205.1579	177.1630,149.1317,135.1162,71.0488
35	1904	35	phenylpropanoid	Trolox	C14 H18 O4	+	8.63	1.75E+06	251.1278	251.1200	187.1109,177.0901,173.0952,133.1005,119.0850
36	2401	36	phenylpropanoid	threo-Syringylglycerol	C11 H16 O6	+	4.51	1.45E+07	245.1020	245.1012	227.0905,209.0800,183.0642,167.0696,153.0543
37	2623	37	phenylpropanoid	Hydroconiferyl Alcohol	C10 H14 O3	+	7.04	1.17E+07	183.1016	183.1008	165.0904,151.0747,137.0593,123.0436
38	2536	38	phenylpropanoid	4-Methylumbelliferone hydrate	C10 H8 O3	+	6.35	6.99E+06	177.0546	177.0538	159.0433,149.0590,133.0642,109.0278
39	2679	39	phenylpropanoid	4-Methoxyhydrocinnamic acid	C10 H12 O3	+	4.53	8.63E+06	181.0859	181.0850	163.0746,153.0903,149.0590,135.0798,121.0642,107.0486,55.0176
40	5	40	phenylpropanoid	NP-021030	C9 H8 O4	+	5.69	5.57E+06	181.0495	181.0486	163.0381,145.0276,153.0538,135.0434,123.0434,111.0435
41	429	1	terpene	Lup-20(29)-ene-3,28-diyl (2E,2'E)bis(3-phenylacry	C48 H62 O4	+	18.84	3.76E+07	703.4721	703.4720	125.0955,121.1005,97.1007,69.0695
42	451	2	terpene	(3beta)-3-(beta-D-Xylopyranosyloxy)olean-12-en	C35 H56 O7	+	18.00	6.35E+05	589.4099	589.4123	127.0384,97.0278,95.0851,89.0593,71.0852,57.0696,55.0540
43	500	3	terpene	Ursolic acid	C30 H48 O3	+	21.34	3.68E+06	457.36762	457.36554	163.1473,109.1006,95.0851,69.0696,67.0539,57.0696
44	513	4	terpene	NP-020112	C30 H48 O2	+	22.98	1.03E+07	441.3727	441.3708	257.2255,189.1629,149.1318,137.1318,123.1162,69.0696,55.0540
45	516	5	terpene	(1R,2R,5S,8R,10R,14R)-20-hydroxy-1,2,14,18,18-pent	C30 H46 O4	+	15.56	3.11E+06	471.3469	471.3468	125.0591,109.0643,107.0851,83.0852,81.0695,79.0539,69.0696,57.0696,55.0540
46	517	6	terpene	(1S,4S,5R,10S,13S,17S,19S,20R)-10-hydroxy-4,5,9,	C30 H46 O3	+	13.68	3.18E+06	455.3520	455.3498	217.1941,153.1268,137.1317,109.1007,97.1007,95.0851,83.0851,81.0695
47	719	7	terpene	(3alpha,5beta,9beta,10alpha,13alpha,15R)-3,15-Di	C22 H36 O4	+	19.68	4.65E+06	365.2686	365.2663	141.0540,125.0592,123.0436,121.0357,89.0593
48	722	8	terpene	3-Methyl-5-(5,5,8a-trimethyl-2-methylene-7-oxo	C22 H36 O3	+	13.33	1.06E+07	349.2737	349.2723	125.0591,109.1007,95.0851,81.0695,55.0540
49	935	9	terpene	(1R,4aS,5R,8aS)-5-(5-hydroxy-3-methylpentyl)-1,4	C20 H34 O3	+	13.42	4.15E+07	323.2581	323.2565	181.1215,177.1630,16.1474,149.1318,69.0696
50	1104	10	terpene	(1R,3aR,7aR)-1-[(2R,3E,5R)-5,6-Dimethyl-3-hepte	C19 H32 O	+	13.43	6.38E+06	277.2526	277.2513	207.1734,179.1424,123.0798,107.0850,81.0695,55.0540
51	1562	11	terpene	NP-004917	C15 H26 O3	+	14.68	5.48E+07	255.1955	255.1943	147.1160,137.0952,101.0591,83.0851,71.0488,69.0695
52	1644	12	terpene	NP-004713	C15 H24 O2	+	8.30	1.02E+07	283.1540	283.1500	181.1214,163.1109,149.1317,123.1162,79.0539,55.0540
53	1653	13	terpene	(-)-Caryophyllene oxide	C15 H24 O	+	11.98	1.77E+08	221.1900	221.1800	177.1629,165.1266,147.1162,121.1006,55.0540
54	1684	14	terpene	(3aR,4aS,5R,6S,8S,9aR)-5,6-dihydroxy-4a,8-dimetl	C15 H22 O4	+	8.88	4.08E+06	267.1591	267.1580	81.0694,57.0332
55	1739	15	terpene	(4Z)-1a,5-Dimethyl-8-methylene-2,3,6,7,7a,8,10a	C15 H20 O3	+	9.94	1.15E+07	249.1485	249.1474	165.0902,137.0954,123.0435,109.0643,83.0488,67.0539
56	1774	16	terpene	DEHYDROCOSTUS LACTONE	C15 H18 O2	+	10.80	9.46E+06	231.1380	231.1369	185.0951,135.1162,95.0487,79.0539,67.0539
57	2590	17	terpene	2-bornene	C10 H16	+	10.27	6.41E+06	137.1325	137.1318	109.1006,95.0851,69.0696,55.0540
58	179	18	terpene	(4aS,5R,6S,8aS)-5-[(3E)-5-methoxy-3-methyl-5-c	C21 H32 O4	−	24.51	1.58E+06	347.2228	347.2222	347.2222
59	197	19	terpene	Octabenzone	C21 H26 O3	−	18.44	1.73E+07	325.1809	325.1810	325.1810
60	76	20	terpene	Isophorone	C9 H14 O	+	8.68	6.54E+06	139.1117	139.1112	83.0488,69.0696,55.0540
61	2073	21	terpene	2-[(1E)-1-Buten-3-yn-1-yl]-1,3,3-trimethylcycloh	C13 H18	+	9.07	9.75E+06	175.1481	175.1472	157.1005,147.1161,133.1005,119.0849,105.0694,91.0538,69.0696
62	2428	22	terpene	1-carboxy-3-hydroxyadamantane	C11 H16 O3	+	7.05	2.30E+08	197.1172	197.1163	179.1058,161.0954,151.1111,133.1006,69.0696
63	1900	23	terpene	1,1,4,4-Tetramethyltetralin	C14 H20	+	8.73	2.41E+06	189.1638	189.0901	161.1317,147.1161,133.1006,119.0850,77.0383
64	454	24	terpene	(1S,2R,3R,3aS,4R,4aR,9aR,10R,10aR)-9a-(Acetoxym	C35 H42 O11	+	21.29	1.46E+07	639.2800	639.2798	538.2555,465.2262,121.0641,97.0643,91.0538,79.0539,71.0488,57.0331
65	457	25	terpene	(2Z)-4-[(1R,2R,7S,16S,18S)-11-Hydroxy-16-metho	C34 H40 O9	+	22.24	1.55E+09	593.2745	593.2738	533.2526,459.2161
66	458	26	terpene	1,2-Bis(benzyloxy)-4-[(5E)-3,6,10-trimethyl-1,5,9-	C34 H40 O2	+	21.27	2.64E+07	481.3101	481.3111	83.0851,81.0695,71.0852,69.0695,67.0539,57.0696,55.0540
67	528	27	terpene	2-phytyl-1,4-dihydroxynaphthalene	C30 H46 O2	+	22.28	4.49E+06	439.3571	439.3553	123.1162,111.1163,109.1006,95.0851,81.0695,57.0696
68	529	28	terpene	(2R)-2-[(2R,3R,3aR,6S,7S,9bR)-6-(2-Carboxyethyl)	C30 H44 O5	+	18.33	6.72E+06	485.3262	485.3235	177.1265,165.1265,151.0747,139.0747,95.0851,93.0694,69.0695
69	1658	29	terpene	(E,E)-alpha-Farnesene	C15 H24	+	10.55	1.87E+06	205.1951	205.1942	163.1473,149.1317,121.1006,95.0851,81.0695,67.0589
70	2222	30	terpene	(3-Hydroxyadamantan-1-yl)acetic acid	C12 H18 O3	+	9.21	7.38E+06	211.1329	211.1321	193.1215,151.1111,133.1006,107.0851,93.0695,55.0540
71	2036	31	terpene	trans-Damascone	C13 H20 O	+	9.07	9.58E+06	193.1587	193.1577	175.1112,138.0988,95.0487,79.0539,67.0589,55.0540
72	536	1	steroid	Sitostenone	C29 H48 O	+	18.45	4.52E+06	413.3778	413.3759	191.1421,137.0954,123.0798,109.0642,83.0487
73	537	2	steroid	OXOSITOSTENONE,6-(P)	C29 H46 O2	+	24.15	1.19E+06	427.3571	427.3552	227.1421,119.0849,95.0851,93.0694,83.0851,81.0695,55.0539
74	594	3	steroid	Bufotalin	C26 H36 O6	+	20.46	1.13E+06	445.2585	455.2570	177.0904,167.0695,149.1317,123.1163,123.0799,123.0435
75	607	4	steroid	Epitestosterone glucuronide	C25 H36 O8	+	11.82	4.10E+06	465.2483	465.2455	229.1942,203.1421,189.1266,137.0955,123.0799,109.0643,71.0489
76	646	5	steroid	Desonide	C24 H32 O6	+	12.94	1.10E+06	417.2272	417.2235	177.0901,163.0746,135.0798,121.0642,69.0696
77	681	6	steroid	2953	C23 H36 O2	+	19.54	1.24E+07	345.2788	345.2773	179.1424,165.1266,137.0954,125.0955,109.1006,81.0695,67.0539,55.0540
78	809	7	steroid	Tetrahydrodeoxycorticosterone	C21 H34 O3	+	20.38	7.60E+06	335.2581	335.2565	123.1163,97.1008,83.0488,68.9968,59.0489,55.0176
79	1044	8	steroid	Exemestane	C20 H24 O2	+	14.62	5.40E+05	297.1849	297.1836	163.1111,131.0850,135.1163,97.1008,81.0696
80	1112	9	steroid	5-Androstenediol	C19 H30 O2	+	15.99	6.41E+07	291.2319	291.2306	81.0695,55.0384
81	1141	10	steroid	Androstadienone	C19 H26 O	+	14.25	5.60E+08	271.2056	271.2045	243.2099,203.1785,189.1265,161.1316,149.0954,123.0799,95.0851,81.0695,67.0539,55.0175
82	1329	11	steroid	19-Norandrostenedione	C18 H24 O2	+	10.66	8.40E+06	273.1849	273.1838	217.1214,213.1628,201.1629,173.1316,123.0798,95.0851,81.0695
83	1341	12	steroid	(+)-Estrone	C18 H22 O2	+	14.90	2.93E+06	271.1693	271.1682	201.1269,149.0954,147.0797,137.0954,121.0642,107.0486,77.0381
84	281	13	steroid	Trenbolone Acetate	C20 H24 O3	−	16.83	1.08E+07	311.1653	311.1653	311.1653
85	437	1	quinone	MK 6	C41 H56 O2	+	15.06	1.43E+06	581.4353	581.4357	189.1629,159.0795,149.1317,109.1006,95.0851,57.0696
86	731	2	quinone	2-Hydroxy-5-methoxy-3-(10-pentadecenyl)benzo	C22 H34 O4	+	17.35	1.30E+08	363.2530	363.2500	205.0487,137.1319,135.1162,121.1007,95.0851,81.0332,67.0539
87	1370	3	quinone	Embelin	C17 H26 O4	+	11.47	9.59E+06	295.1904	295.1892	125.0591,111.0436,97.0643,69.0696
88	1838	4	quinone	1,2,4-Trihydroxyanthraquinone	C14 H8 O5	+	4.59	3.55E+06	257.0444	257.0432	125.0228,77.0382
89	1891	5	quinone	2,6-Di-tert-butyl-1,4-benzoquinone	C14 H20 O2	+	10.1	1.60E+07	221.1536	221.15276	193.1578,83.0852,57.0696
90	2329	6	quinone	J4L83T8MZ5	C12 H10 O7	+	3.67	5.38E+06	267.0499	267.0486	183.0279,141.0177,193.0486,111.0435,83.0488
91	2548	7	quinone	lawsone	C10 H6 O3	+	5.74	1.14E+06	175.0390	175.0388	147.0433,119.0486,105.0329,77.0382
92	576	1	flavonoid	Rutin	C27 H30 O16	+	7.44	4.19E+06	611.1607	611.1589	611.1589
93	578	2	flavonoid	Nictoflorin	C27 H30 O15	+	8.09	5.04E+06	595.1657	595.1637	595.1637
94	581	3	flavonoid	Baohuoside I	C27 H30 O10	+	6.26	834000	515.19117	515.18994	193.0851,192.0771,191.1057,153.0539,137.0227,135.0434,107.0486,127.0384,
95	695	4	flavonoid	5-hydroxy-2-(4-hydroxy-3-methoxyphenyl)-6-m	C23 H24 O12	+	10.23	3.51E+06	493.1341	493.1326	151.0380,147.0431,127.0383,125.0590,123.0432,121.0278,111.0435,107.0486,99.0436
96	758	5	flavonoid	Epigallocatechin gallate	C22 H18 O11	+	5.66	9.04E+07	459.0922	459.0905	147.0432,135.112,123.0436,111.0434,109.0279,81.0331
97	854	6	flavonoid	Astilbin	C21 H22 O11	+	7.33	4.41E+07	451.1235	451.1217	231.0642,195.0280,177.0175,161.0227,153.0176,149.0227,147.0647,123.0436
98	859	7	flavonoid	Myricitrin	C21 H20 O12	+	7.24	96200000	465.10275	465.10092	177.0175,153.0176,137.0228,129.0541,109.0280,85.0281,71.0489
99	868	8	flavonoid	Cynaroside	C21 H20 O11	+	8.04	1.31E+08	449.1078	449.1060	325.0698,179.0331,153.0176,135.0434,121.0279,85.0280
100	869	9	flavonoid	Afzelin	C21 H20 O10	+	8.68	5.89E+07	433.1129	433.1110	177.0173,165.0175,163.0593,145.0488,123.0435,85.0280,69.0332
101	1059	10	flavonoid	Tangeritin	C20 H20 O7	+	11.74	6.06E+06	373.1282	373.1264	133.0642,107.0487
102	1155	11	flavonoid	2-(2,6-dimethoxyphenyl)-5,6-dimethoxy-4H-chro	C19 H18 O6	+	11.57	2.15E+07	343.1176	343.1161	205.0487,181.0486,163.0745,153.0539,151.0380,137.0591,107.0486
103	1344	12	flavonoid	Eupatilin	C18 H16 O7	+	11.19	4.71E+06	345.0968	345.0955	327.0853,207.0643,175.0382,161.0589,153.0176,151.0382,149.0591,131.0486,123.0435,107.0486
104	1520	13	flavonoid	Isorhamnetin	C16 H12 O7	+	9.90	1.12E+07	317.0656	317.0642	165.0176,153.0175,137.0591,123.0435,109.0278
105	1526	14	flavonoid	Hydroxygenkwanin	C16 H12 O6	+	10.00	1.36E+07	301.0707	301.0793	167.0330,135.0434,123.0435
106	1535	15	flavonoid	Formononetin	C16 H12 O4	+	10.31	4.56E+06	269.0808	269.0797	137.0227,133.0642,109.0279,107.0486
107	1785	16	flavonoid	NP-000358	C15 H14 O7	+	3.64	1.30E+08	307.0812	307.0800	181.0487,169.0487,163.0382,153.0539,151.0383,139.0384,135.0435
108	1793	17	flavonoid	Cianidanol	C15 H14 O6	+	4.66	2.84E+07	291.0863	291.0849	181.0487,167.0330,165.0538,163.0382,153.0540,151.0382,139.0383,123.0435,109.0279
110	1794	18	flavonoid	Phloretin	C15 H14 O5	+	7.76	1.55E+06	275.0914	275.0901	151.0381,149.0590,133.0641,121.0642,107.0486
111	1798	19	flavonoid	(±)-Equol	C15 H14 O3	+	8.83	4.57E+06	243.1016	243.1005	149.0590,133.0642,123.0435,119.0487,109.0279,107.0486
112	1803	20	flavonoid	Taxifolin	C15 H12 O7	+	7.16	8.46E+07	305.0656	305.0642	195.0279,167.0331,165.0177,153.0539,153.0175,149.0226,139.0383,137.0227,135.0435
114	1810	21	flavonoid	Eriodictyol	C15 H12 O6	+	5.22	4.40E+07	289.0707	289.0693	247.0597,179.0330,153.0175,137.0229,135.0434,109.0280
115	1811	22	flavonoid	Naringenin	C15 H12 O5	+	9.17	1.54E+07	273.0758	273.0746	231.0645,153.0175,147.0434,133.1006,111.0436,91.0538
116	1815	23	flavonoid	liquiritigenin	C15 H12 O4	+	10.81	4.12E+06	257.0808	257.0797	135.0435,121.0642,119.0486,109.0279
117	1816	24	flavonoid	Myricetin	C15 H10 O8	+	7.24	3.32E+08	319.0448	319.0436	193.0124,165.0174,153.0175,139.0383,137.0227,125.0227,109.0279
118	1821	25	flavonoid	Quercetin	C15 H10 O7	+	8.04	4.88E+08	303.0499	303.0491	165.0173,153.0175,151.0382,137.0227,125.0227,123.0435,109.0279
119	1825	26	flavonoid	Kaempferol	C15 H10 O6	+	8.68	2.96E+08	287.0550	287.0537	165.0175,153.0175,137.0227,135.0435,109.0278,107.0486
120	1831	27	flavonoid	Apigenin	C15 H10 O5	+	9.98	3.34E+07	271.0601	271.0590	153.0175,125.0226,119.0486,109.0278
121	421	28	flavonoid	5,7-Dihydroxy-2-(3-hydroxy-4-methoxyphenyl)ch	C16 H14 O6	−	9.91	1.19E+06	301.0718	301.0716	301.0716
122	467	29	flavonoid	Genistein	C15 H10 O5	−	13.23	6.42E+07	269.0455	269.0444	269.0444
123	549	30	flavonoid	(2E)-1-(3-Ethyl-5,5,8,8-tetramethyl-5,6,7,8-tetrah	C29 H32 O	+	12.01	1.86E+07	397.2526	397.2536	187.1472,173.0954,83.0851,69.0695,67.0539
124	1956	31	flavonoid	Bellidin	C13 H8 O6	+	3.66	1.11E+07	261.0394	261.0382	137.0226,109.0278,97.0279,65.0383
125	1	O1	phenolic acids,	5-carboxyvanillic acid	C9 H8 O6	+	3.68	6.91E+05	213.0394	213.0391	167.0331,150.0305,151.0383,122.0357,107.0487,95.0487,93.0331,79.0539,77.0383
126	3	O2	phenolic acids,	4-oxo-4,5,6,7-tetrahydrobenzo[b]furan-3-carbox	C9 H8 O4	+	4.59	8.63E+06	181.0495	181.0487	163.038,135.0434,125.0591,95.0487,83.0487,81.0331
127	43	O3	phenolic acids,	Cyclobuta[e][1,3]benzodioxole-6,7-dione	C9 H4 O4	+	4.56	3.17E+06	177.0182	177.0173	149.0226,131.0121,121.0278
128	44	O4	phenolic acids,	Azelaic acid	C9 H16 O4	+	8.44	3.72E+06	189.1121	189.1112	147.1161,139.1111,135.0798,95.0851,69.0696,55.0540
129	45	O5	phenolic acids,	9-Oxononanoic acid	C9 H16 O3	+	11.78	1.29E+07	173.1172	173.1165	155.1060,119.0850,109.1007,95.0851,81.0695,67.0539
130	67	O6	phenolic acids,	Ethyl 2-oxocyclohexanecarboxylate	C9 H14 O3	+	8.44	1.68E+07	171.1016	171.1009	97.0644,69.0696,57.0696
131	71	O7	phenolic acids,	(E)-3-cyclohexylacrylic acid	C9 H14 O2	+	11.78	1.56E+07	155.1067	155.1060	137.0955,109.1007,83.0852
132	79	O8	phenolic acids,	1,2,3,4-Tetramethyl-1,3-cyclopentadiene	C9 H14	+	6.70	1.79E+07	123.1168	123.1162	121.1005,108.0927,95.0805,81.0695,55.0539
133	86	O9	phenolic acids,	DC2810000	C9 H12 O3	+	8.68	4.09E+07	169.0859	169.0852	139.0384,137.0591,126.0305,110.0357,107.0486,93.0330,77.0383
134	91	O10	phenolic acids,	4-Ethylguaiacol	C9 H12 O2	+	8.44	7.58E+06	153.0910	153.0904	135.0435,105.0695,91.0538,77.0383
135	104	O11	phenolic acids, ca	Cumene	C9 H12	+	6.09	8100000	121.10117	121.1006	79.0539
136	112	O12	phenolic acids, ca	Ethyl gallate	C9 H10 O5	+	6.35	48900000	199.06009	199.0592	181.0488,171.0281,153.0539,137.0592,109.0280,125.0229,93.0330
137	125	O13	phenolic acids,	3,4-Dimethoxybenzoic acid	C9 H10 O4	+	6.26	1.90E+07	183.0652	183.0645	155.0695,140.0462,123.0435,95.0488,77.0383
138	142	O14	phenolic acids,	LW8000000	C8 H8 O5	+	4.73	1.82E+06	185.0444	185.0436	167.0332,153.0175,139.0384,125.0228,126.0306,123.0435,109.0279
139	143	O15	phenolic acids,	methyl 4-methyl-2-oxo-2H-pyran-6-carboxylate	C8 H8 O4	+	4.80	9.14E+06	169.0495	169.0488	141.0540,125.0592,109.0280,85.0280,65.0383
140	159	O16	phenolic acids,	4-Methoxybenzaldehyde	C8 H8 O2	+	15.67	1.53E+07	137.0597	137.0590	109.0643,107.0485,79.0538
141	170	O17	phenolic acids,	Acetophenone	C8 H8 O	+	6.66	1.52E+06	121.0648	121.0642	105.0694,95.0487,91.0538,77.0382,51.0227
142	173	O18	phenolic acids,	(p-Hydroxyphenyl)glyoxal	C8 H6 O3	+	5.66	1.33E+07	151.0390	151.0382	133.0277,105.0330,77.0382,51.0227
143	181	O19	phenolic acids,	Phthaldialdehyde	C8 H6 O2	+	6.87	1.59E+08	135.0441	135.0434	105.0330,107.0486,79.0539,77.0382,51.0227
144	182	O20	phenolic acids,	2-Hydroxybicyclo[4.2.0]octa-1,3,5-triene-7,8-dion	C8 H4 O3	+	12.91	2.99E+08	149.0233	149.0225	121.0278,93.0330,65.0382
145	194	O21	phenolic acids,	3683	C8 H14 O3	+	6.02	1.21E+06	159.1016	159.1006	113.0592,99.0436,85.0644,81.0332,55.0540
146	196	O22	phenolic acids,	Diethyl Oxalacetate	C8 H12 O5	+	8.56	1.04E+06	189.0758	185.0751	85.0280,81.0331,55.0540
147	201	O23	phenolic acids,	NP-019360	C8 H12 O4	+	4.65	2.60E+06	173.0808	173.0803	137.0592,127.0949,113.0593,111.0436,95.0488,67.0540,55.0540
148	223	O24	phenolic acids,	2-Carbethoxycyclopentanone	C8 H12 O3	+	7.24	3.46E+06	157.0859	157.0851	139.0747,95.0487,67.0539,55.0540
149	227	O25	phenolic acids,	Dimedone	C8 H12 O2	+	6.02	3.84E+06	141.0910	141.0903	123.0798,113.0955,97.0643,71.0488
150	231	O26	phenolic acids,	3721	C8 H12 O	+	8.43	1.06E+07	125.0961	125.0954	95.0850,83.0851,69.0695,57.0696,55.0539
151	237	O27	phenolic acids,	Penicillic acid	C8 H10 O4	+	5.03	1.99E+07	171.0652	171.0644	156.0410,138.0305,127.0384,111.0435,98.0596,69.0332
152	250	O28	phenolic acids,	Guaethol	C8 H10 O2	+	5.62	5.49E+06	139.0754	139.0748	111.0435,93.0331,77.0382,51.0227
153	258	O29	phenolic acids,	5-Ethoxy-2-furoic acid	C7 H8 O4	+	3.90	8.37E+06	157.0495	157.0488	142.0254,112.0150,111.0435,97.0643
154	265	O30	phenolic acids,	2-Methoxyresorcinol	C7 H8 O3	+	4.66	1.77E+07	141.0546	141.0540	126.0306,123.0435,109.0280,77.0383
155	272	O31	phenolic acids,	Gastrodigenin	C7 H8 O2	+	5.66	7.07E+06	125.0597	125.0591	107.0486,95.0487,93.0331,79.0537,77.0382,51.0227
156	282	O32	phenolic acids,	Gallic acid	C7 H6 O5	+	6.35	2.34E+07	171.02879	171.028	153.0176,127.0384,125.0384,109.0279,93.0330
157	291	O33	phenolic acids,	Gallaldehyde	C7 H6 O4	+	7.81	5.67E+06	155.0339	155.0331	137.0227,125.0227,109.0279,93.0330
158	296	O34	phenolic acids,	3,4-Dihydroxybenzaldehyde	C7 H6 O3	+	3.64	4.53E+07	139.0390	139.0383	121.0278,93.0330
159	308	O35	phenolic acids,	4-Hydroxybenzaldehyde	C7 H6 O2	+	16.01	9.39E+06	123.0441	123.0434	95.0487,79.0539,67.0539,65.0383

160	310	O36	phenolic acids,	Benzoic acid	C7 H6 O2	+	6.23	8.00E+06	123.0441	123.0434	95.0487,79.0538,77.0382,65.0383,51.0227
161	313	O37	phenolic acids,	5,6-Dioxo-1,3-cyclohexadiene-1-carboxylic acid	C7 H4 O4	+	7.57	1.38E+07	153.0182	153.0175	125.027,97.0279,83.0123
162	315	O38	phenolic acids,	5-Hydroxy-1,3-benzodioxol-2-one	C7 H4 O4	+	6.35	8.75E+06	153.0182	153.0177	92.0254,81.0332,3.0227
163	320	O39	phenolic acids,	D-pinitol	C7 H14 O6	+	1.14	4.35E+06	195.0863	195.0857	159.0645,148.051,120.0802,69.0332
164	326	O40	phenolic acids,	butyl acrylate	C7 H12 O2	+	6.05	9.22E+05	129.0910	129.0541	111.0435,73.0281,69.0696
165	330	O41	phenolic acids,	Succinylacetone	C7 H10 O4	+	4.00	4.96E+06	159.0652	159.0645	141.0540,132.1337,113.0592,85.0281,57.0332
166	331	O42	phenolic acids,	dimethyl itaconate	C7 H10 O4	+	21.29	4.27E+06	159.0652	159.0644	119.0849,115.0536,99.0436,710488,55.0540
167	341	O43	phenolic acids,	β,β-Dimethyl-γ-methylene-γ-butyrolactone	C7 H10 O2	+	6.17	3.76E+06	127.0754	127.0747	99.0436,85.0280,83.0852,55.0540
168	347	O44	phenolic acids,	5-hydroxy-4-methoxy-5,6-dihydro-2H-pyran-2-	C6 H8 O4	+	3.10	2.01E+07	145.0495	145.0489	127.0385,99.0437,85.0281,71.0486
169	355	O45	phenolic acids,	2,5-Bis(hydroxymethyl)furan	C6 H8 O3	+	22.61	5.07E+08	129.0546	129.0539	111.0435
170	356	O46	phenolic acids,	3,5-Dihydroxy-2-(hydroxymethyl)-4H-pyran-4-o	C6 H6 O5	+	2.44	1.63E+06	159.0288	159.0281	141.0176,113.0228,85.0283,67.0175,55.0176
171	359	O47	phenolic acids,	Kojic acid	C6 H6 O4	+	4.34	3.89E+07	143.0339	143.0332	125.0227,115.0384,87.0436,83.0488,53.0020
172	365	O48	phenolic acids,	5-Hydroxymethyl-2-furaldehyde	C6 H6 O3	+	6.35	2.90E+07	127.0390	127.0384	109.0643,99.0436,97.0280,81.0331,53.0383
173	379	O49	phenolic acids,	MFCD00177095	C6 H4 O4	+	2.48	3.36E+06	141.0182	141.0175	123.0071,113.0228,97.0280,95.0123,81.0321,85.0280,55.0176
174	385	O50	phenolic acids,	4-Oxo-4H-pyran-2-carbaldehyde	C6 H4 O3	+	2.62	1.37E+06	125.0233	125.0227	97.0279,83.0124,69.0332,53.0019
175	391	O51	phenolic acids,	Furo[3,2-b]furan-2,5-dione	C6 H2 O4	+	2.10	9.23E+05	139.0026	139.0019	111.0071,95.0123,68.9968,55.0176
176	443	O52	phenolic acids,	Antioxidant CA	C37 H52 O3	+	19.91	1.17E+08	545.3989	545.3999	55.0539
177	456	O53	phenolic acids,	3-Hydroxy-5-oxo-5-(tetradecyloxy)-3-[(tetradecy	C34 H64 O7	+	19.60	1.26E+07	585.4725	585.4733	97.1007,87.0437,85.1008,83.0852,73.0645,71.0852,69.0696,57.0696
178	558	O54	phenolic acids,	5-{(2R,3S)-6-Hydroxy-2-(4-hydroxyphenyl)-4-[(E	C28 H22 O6	+	8.57	1.19E+07	455.1489	455.1471	199.0744,139.0383,133.0278,123.0425,107.0487,95.0487
179	583	O55	phenolic acids,	C12E7	C26 H54 O8	+	16.44	9.32E+07	495.3891	495.3876	221.1374,177.1114,133.0854,89.0593,71.0852,57.0696
180	617	O56	phenolic acids,	C12E6	C24 H50 O7	+	16.44	8.14E+07	451.3629	451.3614	195.1219,177.1113,133.0853,89.0593,71.0852,57.0696
181	619	O57	phenolic acids,	2106657	C24 H42	+	13.65	3.29E+07	331.3359	331.3380	135.1161,121.1006,97.1006,93.0695,85.1008,79.0538,57.0696
182	631	O58	phenolic acids,	MFCD02063373	C24 H38 O4	+	20.52	3.15E+08	391.2843	391.2837	179.0695,167.0331,153.0539,137.0590,71.0852,57.0696
183	657	O59	phenolic acids,	2-Arachidonoyl glycerol	C23 H38 O4	+	13.83	2.42E+06	379.2843	379.2827	133.1006,121.1006,109.1007,107.0850,95.0851,87.0437
184	661	O60	phenolic acids,	5-[(8E)-8-Heptadecen-1-yl]-1,3-benzenediol	C23 H38 O2	+	22.28	1.16E+07	347.2945	347.2928	235.1684,179.1058,165.0008,163.0746,161.0596,151.0747,123.0436,83.0852
185	680	O61	phenolic acids,	4-(5,5-dimethyl-1,3-dioxan-2-yl)-4'-heptylbutyrc	C23 H36 O3	+	19.59	2.38E+07	361.2737	361.2720	175.1108,161.0953,159.0795,129.0693,91.0538,69.0696,57.0696
186	718	O62	phenolic acids,	Docosatrienoic acid	C22 H38 O2	+	20.94	3.64E+06	335.2945	335.2929	125.0592,89.0593,71.0852,57.0696
187	727	O63	phenolic acids,	MFCD00038340	C22 H36 O2	+	18.44	1.74E+08	333.2788	333.2774	151.0746,125.0591,109.0643,107.0486,83.0851,69.0695,55.0540
188	740	O64	phenolic acids,	Docosapentaenoic acid	C22 H34 O2	+	13.11	1.01E+07	331.2632	331.2617	147.1162,133.1006,121.1006,107.0851,95.0851,93.0695,67.0539,55.0540
189	786	O65	phenolic acids,	1-Linoleoyl glycerol	C21 H38 O4	+	18.08	1.82E+07	355.2843	355.2826	175.1474,147.1162,135.1163,123.1163,109.1007,95.0851,81.0695,67.0539
190	812	O66	phenolic acids,	5-[(Z)-Pentadec-8-enyl]benzene-1,3-diol	C21 H34 O2	+	18.87	1.60E+08	319.2632	319.2617	179.1059,165.0902,151.0747,137.0591,123.0435,69.0696,55.0540
191	825	O67	phenolic acids,	5-[(8Z,11Z)-pentadeca-8,11-dien-1-yl]benzene-1	C21 H32 O2	+	16.54	1.22E+07	317.2475	317.2460	177.0902,163.0746,149.0590,137.0591,123.0435,83.0852
192	889	O68	phenolic acids,	Ethyl oleate	C20 H38 O2	+	11.61	1.15E+06	311.2945	311.2929	149.0954,137.0955,125.0955,107.0850,95.0851,81.0695,67.0539,55.0540
193	911	O69	phenolic acids,	Ethyl Linoleate	C20 H36 O2	+	24.50	4.42E+06	309.2788	309.2774	161.1318,147.1162,133.1006,109.1007,95.0851,81.0695,69.0696
194	946	O70	phenolic acids,	8Z,11Z,14Z-Eicosatrienoic acid	C20 H34 O2	+	10.16	5.21E+06	307.2632	307.2616	109.0643,95.0488,69.0696,55.0540
195	964	O71	phenolic acids,	2K2DJ01BB7	C20 H32 O2	+	15.66	1.35E+08	305.2475	305.2463	95.0851,83.0852,69.0696,67.0539,55.0540
196	1072	O72	phenolic acids,	PROPYLENEGLYCOLDIOCTANOATE	C19 H36 O4	+	15.98	7.83E+06	329.2686	329.2673	117.0541,85.0281,61.0281,57.0332
197	1078	O73	phenolic acids,	6132055	C19 H36 O3	+	19.31	4.59E+07	313.2737	313.2723	125.1319,111.1163,97.1007,85.1008,71.0852,57.0696
198	1181	O74	phenolic acids,	(+/-)12(13)-DiHOME	C18 H34 O4	+	19.38	1.09E+07	315.2530	315.2514	111.0799,109.1007,101.0593,97.0644,95.0851,83.0852,57.0696
199	1189	O75	phenolic acids,	ricinelaidic acid	C18 H34 O3	+	13.45	1.18E+07	299.2581	299.2566	165.1267,155.1424,141.1268,111.1164,97.1008,71.0852,57.0696
200	1250	O76	phenolic acids,	Koiganal II	C18 H32 O	+	20.20	9.63E+06	265.2526	265.2513	153.1267,139.1111,125.0955,111.0799,97.0643,71.0852,57.0696
201	1265	O77	phenolic acids,	NP-001445	C18 H30 O5	+	14.62	4.40E+07	327.2166	327.2151	169.0851,155.0695,127.0384,109.1007,95.0851,69.0696
202	1279	O78	phenolic acids,	9-Oxo-10(E),12(E)-octadecadienoic acid	C18 H30 O3	+	10.94	8.20E+07	295.2268	295.2250	179.1421,151.1110,111.0799,99.0800,71.0852,57.0696
203	1286	O79	phenolic acids,	Pinolenic acid	C18 H30 O2	+	12.76	2.60E+07	279.2319	279.2305	137.1320,111.1163,97.1008,57.0696
204	1324	O80	phenolic acids,	3,4-Dihydro-6-octyl-1(2H)-naphthalenone	C18 H26 O	+	10.95	2.75E+06	259.2056	259.2042	189.1629,147.0797,133.1006,119.0850,109.1006,91.0538
205	1342	O81	phenolic acids,	4,4'-Diisopropylbiphenyl	C18 H22	+	10.56	1.33E+06	239.1794	239.1783	197.1315,119.0850,105.0694,77.0381
206	1381	082	phenolic acids,	Ethyl 2,5-dimethoxy-Î¶-oxobenzeneheptanoate	C17 H24 05	+	11.72	5.59E+06	309.16965	309.16827	179.1422,165.0539,155.1059,139.1111,137.0591,107.0487
207	1403	083	phenolic acids,	Palmitic acid	C16 H32 02	+	16.41	8.25E+06	257.2475	257.24634	61.0281
208	1438	O84	phenolic acids,	(Z)-Isoambrettolide	C16 H28 O2	+	11.30	3.84E+07	253.2162	253.2150	139.1110,137.1318,111.0799,97.1007,69.0695
209	1460	O85	phenolic acids,	Benzo-18-crown-6	C16 H24 O6	+	9.58	9.48E+05	313.1646	313.1631	163.0745.137.0591.119.0486.115.0747.71.0488.59.0489
210	1466	O86	phenolic acids,	NP-004038	C16 H24 O3	+	8.40	1.73E+06	265.1798	265.1786	191.1058,175.1472,153.0903,151.0746
211	1467	O87	phenolic acids,	10-Phenyldecanoic acid	C16 H24 O2	+	11.13	6.68E+06	249.1849	249.1838	203.1420,175.1473,147.1161,133.1005,139.1111,97.0643
212	1508	O88	phenolic acids,	1-O-[2-(Ethoxycarbonyl)benzoyl]-beta-D-glucopy	C16 H18 O10	+	6.14	3.77E+07	371.0973	371.0955	219.0642,209.0435,197.0437,177.0539,133.0278,111.0436,5.0281
213	1607	O89	phenolic acids,	1,9-NONANEDIOL DIACRYLATE	C15 H24 O4	+	10.47	5.19E+06	269.1747	269.1734	195.1370,167.1058,155.1059,141.0902,123.1162,83.0852,69.0696
214	1694	O90	phenolic acids,	Gemfibrozil	C15 H22 O3	+	9.78	8.61E+06	251.1642	251.1631	205.1577,149.0954,123.0799,105.0694,87.0437,69.0332
215	1705	O91	phenolic acids,	3,5-ditert-butyl-4-hydroxybenzaldehyde	C15 H22 O2	+	9.57	2.74E+07	235.1693	235.1681	207.1736,179.1058,161.0954,151.1110,57.0697
216	1708	O92	phenolic acids,	4-Octylbenzaldehyde	C15 H22 O	+	8.73	3.76E+07	219.1743	219.1733	191.1786,121.0643,107.0487,91.0538,57.0696
217	1709	O93	phenolic acids,	1,8-Cyclopentadecadiyne	C15 H22	+	11.99	2.16E+08	203.1794	203.1787	175.1472,161.1317,147.1161
218	1745	094	phenolic acids,	Turmerone	C15 H20 0	+	8.83	8.61E+06	217.15869	217.15793	175.1109,161.0955,133.1006,125.0956,119.0851,91.0539,83.0489,55.0540
219	1748	095	phenolic acids,	(1E,3E)-1,3-Nonadien-1-ylbenzene	C15 H20	+	8.72	2.08E+07	201.16377	201.16309	145.1004,131.0849,123.1162,117.0693,103.0537,97.1006,91.0538,77.0382,71.0851
220	1773	096	phenolic acids,	Loxoprofen	C15 H18 03	+	8.63	4.75E+07	247.13287	247.13197	201.1264,187.1109,163.0746,105.064,91.0538,83.0488,69.0332
221	1781	097	phenolic acids,	4-methoxy-6-[2-(4-methoxyphenyl)ethyl]-2H-p	C15 H16 04	+	8.5	1.21E+06	261.11213	261.11105	163.0746,135.0798,121.0642,107.0486,111.0435,83.0488
222	1783	098	phenolic acids,	bisphenol A	C15 H16 02	+	8.65	1.99E+07	229.1223	229.12143	211.1116,135.0799
223	1874	099	phenolic acids,	MFCD00014547	C14 H22 03	+	9.51	7.17E+06	239.16417	239.16319	193.1579,141.0903,127.0384,85.0281,71.0853
224	1884	O100	phenolic acids,	NP-020240	C14 H20 O3	+	9.25	2.50E+07	237.1485	237.1475	181.0850,177.1265,163.0746,95.0851
225	1905	O101	phenolic acids,	4-(4-Butylphenyl)-4-oxobutanoic acid	C14 H18 O3	+	8.39	2.78E+07	235.1329	235.1318	189.1265,179.0693,175.1109,161.0953,133.1005
226	1909	O102	phenolic acids,	2-Pentyl-1-indanone	C14 H18 O	+	10.09	1.47E+07	203.1430	203.1420	175.1473,117.0693,107.0486,77.0383
227	1916	O103	phenolic acids,	(2S,4R,5S,6S,7R)-5,6,12,14-tetrahydroxy-4-(hydro:	C14 H16 O9	+	4.56	5.24E+08	329.0867	329.0855	207.0642,197.0435,139.0384,85.0280,69.0332
228	1941	O104	phenolic acids,	1-(1,8-dihydroxy-3,6-dimethyl-2-naphthyl)ethan	C14 H14 O3	+	10.45	3.43E+06	231.1016	231.1006	187.0746
229	1989	O105	phenolic acids,	12-Tridecynoic acid	C13 H22 O2	+	8.39	1.41E+07	211.1693	211.1684	193.1578,133.1006,111.0435,109.1007,95.0851,67.0539
230	2014	O106	phenolic acids,	NP-007909	C13 H20 O3	+	6.70	2.38E+07	225.1485	225.1476	207.1370,125.0591,123.1163,109.1007,95.0851,85.0281,81.0695
231	2044	O107	phenolic acids,	Heptylbenzene	C13 H20	+	9.26	2.67E+06	177.1638	177.1629	135.1162,121.1006,93.0694,79.0538,97.1006
232	2052	O108	phenolic acids,	1-(3-ethyl-2,4-dihydroxy-6-methoxyphenyl)butar	C13 H18 O4	+	8.78	9.92E+06	239.1278	239.1268	135.0799,111.0436,105.0694,83.0488,55.0540
233	2063	O109	phenolic acids,	p-Hexyloxybenzoic Acid	C13 H18 O3	+	6.68	2.07E+07	223.1329	223.1320	205.1213,165.0903,97.0643,95.0487,59.0489
234	2066	O110	phenolic acids,	Heptanophenone	C13 H18 O	+	9.09	3.97E+07	191.1430	191.1421	133.1006,105.0694,91.0538
235	2091	O111	phenolic acids,	1-[4-hydroxy-3-(3-methylbut-2-en-1-yl)phenyl]e	C13 H16 O2	+	6.66	9.29E+06	205.1223	205.1216	163.1109,145.1005,135.1162,121.0642,107.0850,105.0694,91.0538
236	2097	O112	phenolic acids,	Cyclohexyl phenyl ketone	C13 H16 O	+	6.69	3.46E+07	189.1274	189.1264	159.0797,143.0848,131.0849,117.0693,91.0538
237	2103	O113	phenolic acids,	4-Pentylphenylacetylene	C13 H16	+	9.08	8.52E+06	173.1325	173.1316	143.0849,117.0693,91.0538,67.0539
238	2117	O114	phenolic acids,	(2R)-5-Methoxy-2-methyl-2,3,8,9-tetrahydro-4H	C13 H14 O4	+	8.05	1.00E+09	235.0965	235.0956	191.0694,175.0747,67.0176,55.0540
239	2128	O115	phenolic acids,	(7-Methoxy-1-naphthyl)acetic acid	C13 H12 O3	+	10.81	3.37E+06	217.0859	217.0851	199.0744,187.0745,185.0588,157.0640
240	2129	O116	phenolic acids,	Monobenzone	C13 H12 O2	+	6.83	1.70E+06	201.0910	201.0906	123.0434,109.0279,91.0538,77.0383
241	2164	O117	phenolic acids,	4-oxododecanedioic acid	C12 H20 O5	+	7.93	1.23E+07	245.1384	245.1373	189.1112,171.1008,125.0955,55.0540
242	2181	O118	phenolic acids,	NP-000124	C12 H20 O4	+	9.21	3.33E+06	229.1434	229.1425	201.1264,123.0799,107.0860,95.0851,67.0539,55.0176
243	2189	O119	phenolic acids,	Traumatin	C12 H20 O3	+	10.94	1.89E+07	213.1485	213.1476	153.1266,149.0954,121.1006,93.0694,69.0695
244	2192	O120	phenolic acids,	3-(8a-Hydroxy-2-oxohexahydro-2H-chromen-4a	C12 H18 O5	+	8.59	4.48E+06	243.1227	243.1217	195.1163,179.1058,165.0902,151.0747,137.0954,109.1007,79.0539
245	2204	O121	phenolic acids,	NP-021018	C12 H18 O4	+	8.68	5.94E+07	227.1278	227.1267	191.1058,181.1215,169.0851,163.1110,121.1006,111.0436,67.0539
246	2224	O122	phenolic acids,	Sedanolide	C12 H18 O2	+	10.94	3.32E+07	195.1380	195.1370	149.1318,107.0850,93.0695,83.0488,81.0695,55.0540
247	2235	O123	phenolic acids,	6-Phenyl-1-hexanol	C12 H18 O	+	13.68	1.07E+07	179.1430	179.1421	161.0589,147.0432,133.1005,105.0693,91.0538,77.0382
248	2240	O124	phenolic acids,	1-t-butyl-4-ethylbenzene	C12 H18	+	9.91	3.18E+06	163.1481	163.1110	135.1162,55.0540
249	2250	O125	phenolic acids,	5-hydroxy-4-[3-(2-hydroxypropan-2-yl)oxiran-2-	C12 H16 O5	+	4.57	1.45E+06	241.1071	241.1061	181.0850,177.0901,163.0745,111.0435,83.0488
250	2252	O126	phenolic acids,	Senkyunolide H	C12 H16 O4	+	8.26	8.48E+06	225.1121	225.1112	189.0901,179.1059,165.0903,161.0954,151.0747,137.0955,105.0695,67.0539
251	2255	O127	phenolic acids,	6-Phenylhexanoic acid	C12 H16 O2	+	9.19	5.24E+07	193.1223	193.1213	151.0747,133.1005,119.0849,105.0694,91.0538,77.0382
252	2264	O128	phenolic acids,	4'-tert-Butylacetophenone	C12 H16 O	+	10.94	1.05E+07	177.1274	177.1264	159.1161,133.1005,119.0486,105.0330,77.0383,57.0695
253	2268	O129	phenolic acids,	MFCD00128082	C12 H16	+	6.57	8.10E+06	161.1325	161.1317	159.1161,133.1006,119.0850,91.0538,69.0696
254	2277	O130	phenolic acids,	Diethyl phthalate	C12 H14 O4	+	8.54	3.42E+06	223.0965	223.0955	187.0745,163.0382,135.0434
255	2307	O131	phenolic acids,	2-Methoxy-6,7,8,9-Tetrahydro-Benzocyclohepten	C12 H14 O2	+	8.19	2.88E+07	191.1067	191.1057	176.0823,163.1109,133.1006,107.0486
256	2312	O132	phenolic acids,	5,8-DIMETHYL-1-TETRALONE	C12 H14 O	+	7.78	3.02E+06	175.1117	175.1108	147.1163,133.1006,105.0693,55.0540
257	2313	O133	phenolic acids,	GW6600000	C12 H14	+	10.94	1.05E+07	159.1168	159.1160	131.0849,129.0692,117.0693,105.0694,91.0538,77.0382,67.0539
258	2328	O134	phenolic acids,	naphthalen-1-ethanol	C12 H12 O	+	8.58	2.80E+06	173.0961	173.0952	153.0689,141.0692
259	2344	O135	phenolic acids,	NP-001846	C11 H20 O4	+	8.46	4.28E+06	217.1434	217.1426	181.1215,171.1009,143.1061,125.0956,115.1112,97.1008
260	2358	O136	phenolic acids,	Ethyl 2-acetylheptanoate	C11 H20 O3	+	8.15	6.59E+05	201.1485	201.1478	201.1477,155.1060,111.0799,85.0280,71.0488,57.0696
261	2366	O137	phenolic acids,	NP-000919	C11 H18 O4	+	8.29	2.17E+06	215.1278	215.1269	179.1058,169.1214,151.1110,101.0227,73.0281
262	2381	O138	phenolic acids,	3,3-Dimethyl-1,5-dioxaspiro(5.5)undecan-9-one	C11 H18 O3	+	11.98	2.82E+05	199.1329	199.1319	181.1216,125.0592,115.0748,95.0488,85.0645
263	2383	O139	phenolic acids,	YQ3683000	C11 H18 O2	+	7.82	1.09E+07	183.1380	183.1371	165.1266,137.1319,123.1162,115.0748,109.1007,95.0851,81.0695,67.0539
264	2393	O140	phenolic acids,	3422	C11 H18 O	+	6.10	1.31E+07	167.1430	167.1422	69.0332,67.0539,53.0383
265	2417	O141	phenolic acids,	2,3,4,5-Tetramethoxytoluene	C11 H16 O4	+	7.37	6.67E+06	213.1121	213.1112	181.0851,151.0747
266	2431	O142	phenolic acids,	5-Pentylresorcinol	C11 H16 O2	+	9.33	9.54E+07	181.1223	181.1214	163.1110,125.0591,111.0436
267	2436	O143	phenolic acids,	Jasmone	C11 H16 O	+	10.80	9.18E+06	165.1274	165.1266	137.1317,123.0798,109.0643,95.0851,67.0539
268	2446	O144	phenolic acids,	n-Amylbenzene	C11 H16	+	10.94	1.42E+07	149.1325	149.1318	93.0695,79.0539
269	2460	O145	phenolic acids,	Butyl benzoate	C11 H14 O2	+	7.07	2.46E+07	179.1067	179.1058	137.0591,135.0435,123.0435,105.0331,91.0538,77.0383
270	2465	O146	phenolic acids,	Valerophenone	C11 H14 O	+	8.68	1.04E+07	163.1117	163.1110	105.0330,77.0382,69.0696
271	2474	O147	phenolic acids,	MFCD00027233	C11 H14	+	10.81	7.71E+06	147.1168	147.1160	119.0849,105.0693,91.0538,77.0383,69.0696
272	2492	O148	phenolic acids,	4-oxo-5-phenylpentanoic acid	C11 H12 O3	+	7.55	3.72E+07	193.0859	193.0850	175.0746,165.0902,147.0798,133.0642,119.0486,91.0538,77.0382
273	2508	O149	phenolic acids,	4-Methyl-1-Tetralone	C11 H12 O	+	8.26	1.93E+07	161.0961	161.0953	133.1005,119.0849,105.0693,83.0487,77.0383,55.0175
274	2524	O150	phenolic acids,	beta-styrylacrylic acid	C11 H10 O2	+	7.55	1.04E+07	175.0754	175.0745	147.0798,129.0692,103.0538,97.0280,77.0383
275	2551	O151	phenolic acids,	NP-019374	C10 H18 O4	+	8.44	1.16E+07	203.1278	203.1268	185.1164,171.1008,143.1059,125.0955,115.0749,87.0437
276	2566	O152	phenolic acids,	2361	C10 H18 O2	+	6.81	1.51E+06	171.1380	171.1370	153.1266,127.0749,111.1163,99.0436,83.0488,55.0176
277	2573	O153	phenolic acids,	DEEMM	C10 H16 O5	+	5.69	1.16E+06	217.1071	217.1063	157.0852,155.0695,143.0696,69.0332,57.0332
278	2575	O154	phenolic acids,	2,2'-(1,1-Cyclohexanediyl)diacetic acid	C10 H16 O4	+	7.01	9.42E+06	201.1121	201.1112	183.1007,165.0903,155.1060,141.0496,137.0955,95.0851
279	2578	O155	phenolic acids,	Ethyl 2-oxocycloheptanecarboxylate	C10 H16 O3	+	8.44	3.19E+07	185.1172	185.1166	157.1216,139.0748,125.0955,101.0592
280	2579	O156	phenolic acids,	trans-geranic acid	C10 H16 O2	+	7.33	2.52E+07	169.1223	169.1215	151.1111,123.1163,113.0592,85.0280,69.0332,57.0696
281	2580	O157	phenolic acids,	Citral	C10 H16 O	+	8.15	1.43E+07	153.1274	153.1267	135.1162,125.1319,111.0799,97.0644,71.0488,55.0540
282	2600	O158	phenolic acids,	Guaifenesin	C10 H14 O4	+	6.65	2.87E+06	199.0965	199.0958	181.0852,155.0696,137.0592,107.0487,95.0488
283	2629	O159	phenolic acids,	6-Pentyl-2H-pyran-2-one	C10 H14 O2	+	6.25	1.77E+07	167.1067	167.1059	149.0954,125.0955,95.0487,83.0852
284	2637	O160	phenolic acids,	2-sec-Butylphenol	C10 H14 O	+	6.10	1.35E+07	151.1117	151.1111	133.1005,103.0537,95.0487,77.0382
285	2642	O161	phenolic acids,	CY9070000	C10 H14	+	6.49	1.48E+07	135.1168	135.0434	93.0695,79.0539
286	2658	O162	phenolic acids,	2,3,4-Trimethoxybenzoic acid	C10 H12 O5	+	4.20	3.74E+06	213.0758	213.0749	195.0643,167.0695,165.0539,151.0383,137.0591
287	2685	O163	phenolic acids,	1-Phenyl-2-butanone	C10 H12 O	+	6.49	1.36E+07	149.09609	149.0954	121.0642,119.0487,91.0538,77.0383,57.0332
288	2692	O164	phenolic acids,	2-ISOPROPENYLTOLUENE	C10 H12	+	9.08	6.21E+07	133.1012	133.1005	91.0538
289	2697	O165	phenolic acids,	resorcinol-o,o'-diacetic acid	C10 H10 O6	+	4.60	4.38E+06	227.0550	227.0541	181.0487,165.0538,135.0435,123.0435,107.0486
290	2707	O166	phenolic acids,	Dimethyl terephthalate	C10 H10 O4	+	5.64	2.30E+06	195.06518	195.06442	163.0382,153.0539,135.0434
291	326	O167	phenolic acids,	(10E,15Z)-9,12,13-Trihydroxy-10,15-octadecadier	C18 H32 O5	-	23.71	4.62E+06	327.21769	327.21658	59.0129,57.0336

NB site			Compound category	Name	Formula	Ion mode	RT	Response	heoretical valu	Measured value	MS/MS
1	1	4	phenylpropanoid	3,5,7-Trihydroxy-4H-chromen-4-one	C9 H6 O5	+	3.69	2.48E+07	195.0288	195.0279	177.0173,149.0226,135.0434,123.0435,109.0279
2	2	8	phenylpropanoid	Umbelliferone	C9 H6 O3	+	4.42	8.69E+06	163.0390	163.0382	145.0277,123.0434,109.0279,93.0330,55.0176
3	3	15	phenylpropanoid	Coumarin	C9 H6 O2	+	5.21	1.20E+06	147.0441	147.0433	119.0485,107.0486,103.0537,77.0382
4	4	376	phenylpropanoid	Arctigenin-1	C21 H24 O6	+	7.94	2.57E+06	373.1646	373.1626	373.1626
5	5	610	phenylpropanoid	(1r,3R,4s,5S)-4-{[(2E)-3-(3,4-dihydroxyphenyl)prop-	C16 H18 O9	+	4.92	5.20E+06	355.1024	355.1007	193.0486,163.0382,135.0434,85.0281
6	6	827	phenylpropanoid	Diethyl benzalmalonate	C14 H16 O4	+	5.49	1.20E+07	249.1121	249.1110	203.0694,189.0901,175.0746,161.0590,147.0434,91.0538
7	7	925	phenylpropanoid	CHEMBRDG-BB 6367113	C13 H14 O3	+	5.72	1.25E+07	219.1016	219.1006	201.0902,191.1057,123.0435,107.0487
8	8	1012	phenylpropanoid	7-Hydroxy-4-propylcoumarin	C12 H12 O3	+	12.91	1.77E+07	205.0859	205.0852	177.0903,163.0745,109.0279
9	9	1014	phenylpropanoid	(5,7-Dihydroxy-4-methyl-2-oxo-2H-chromen-3-yl)	C12 H10 O6	+	4.62	1.44E+08	251.0550	251.0542	191.0329,177.0537,135.0433,71.0124
10	10	1016	phenylpropanoid	6,7-Dihydroxycoumarin-4-acetic Acid	C11 H8 O6	+	3.67	4.78E+07	237.0394	237.0384	191.0329,177.0539,109.0278
11	11	1018	phenylpropanoid	6-Methoxy-4-oxo-4H-chromene-2-carboxylic acid	C11 H8 O5	+	4.60	8.20E+06	221.0444	221.0436	193.0486,177.0538,175.0383,149.0591,123.0434,107.0486
12	12	1080	phenylpropanoid	Scoparone	C11 H10 O4	+	4.42	1.07E+07	207.0652	207.0646	179.0693,175.0383,165.0538
13	13	1085	phenylpropanoid	(4,6-Dihydroxy-3-oxo-1,3-dihydro-2-benzofuran-1	C10 H8 O6	+	4.60	7.82E+06	225.0394	225.0385	207.0279,181.0488,179.0330,165.0173,137.0225,109.0279
14	14	1087	phenylpropanoid	Fraxetin	C10 H8 O5	+	4.62	8.27E+07	209.04444	209.04379	191.0329,181.0485,177.0173,165.0538
15	15	1095	phenylpropanoid	Scopoletin	C10 H8 O4	+	6.21	2.16E+06	193.0495	193.0485	175.0381,165.0539,149.0589
16	16	1099	phenylpropanoid	hymecromone	C10 H8 O3	+	4.55	2.56E+06	177.0546	177.0537	149.0589,109.0279,93.0331
17	17	1104	phenylpropanoid	2-oxo-2H-chromene-3-carboxylic acid	C10 H6 O4	+	4.60	1.47E+07	191.0339	191.0329	163.0381,147.0435,145.0277,105.0330
18	18	1176	phenylpropanoid	(E)-2-Methoxycinnamaldehyde	C10 H10 O2	+	4.53	2.37E+06	163.0754	163.0747	131.0485,121.1006,107.0486,91.0538,77.0382,55.0176
19	19	31	phenylpropanoid	3-Phenyl-1-propanol	C9 H12 O	+	8.04	7.94E+06	137.0961	137.0954	119.0849,107.0486,105.0694,91.0538,77.0383,51.0226
20	20	43	phenylpropanoid	3-Phenylpropanoic acid	C9 H10 O2	+	7.42	1.27E+06	151.0754	151.0746	123.0798,105.0694,103.0538,91.0538,77.0383
111	21	1011	phenylpropanoid	2-(2-Methoxyphenyl)-5-oxotetrahydro-3-furancarb	C12 H12 O5	+	6.34	1.75E+06	237.0758	237.0748	163.0746,137.0591,121.0642,109.0643,85.0280
21	1	210	terpene	Oleanolic acid benzoate	C37 H52 O4	+	16.47	2.13E+08	561.3938	561.3947	561.3947
22	2	233	terpene	Ursolic acid	C30 H48 O3	+	10.19	1.99E+06	457.3676	457.3656	181.1215,163.1472,95.0850,55.0539
23	3	241	terpene	(1S,4S,5R,10S,13S,17S,19S,20R)-10-hydroxy-4,5,9,9,1	C30 H46 O3	+	10.98	1.20E+07	455.3520	455.3500	301.2158,193.1216,179.1057,137.1318,95.0851,55.0540
24	4	639	terpene	NP-004917	C15 H26 O3	+	7.07	2.50E+06	255.1955	255.1945	179.1423,151.1109,147.1161,143.1059,137.0954,101.0592,87.0436,69.0695
25	5	651	terpene	1,4-dihydroxy-1,4-dimethyl-7-(propan-2-ylidene)-	C15 H24 O3	+	7.35	1.91E+06	253.1798	253.1786	199.1472,189.1629,177.1265,175.1472,125.0954,111.0799,81.0695,67.0539
26	6	661	terpene	(-)-Caryophyllene oxide	C15 H24 O	+	9.30	2.40E+06	221.1900	221.1892	193.1584,177.1628,163.1474,147.1161,123.1162,109.1006
27	7	720	terpene	(3aR,8R,8aR,9aR)-8-Hydroxy-8a-methyl-3,5-bis(me	C15 H20 O3	+	6.19	4.24E+06	249.1485	249.1476	203.1421,185.1316,163.0746,159.1162,123.0435,97.0280,67.0539,55.0540
28	8	863	terpene	4-(4-Hydroxy-4-methylpentyl)cyclohex-3-ene-1-ca	C13 H22 O2	+	8.17	1.04E+07	211.1693	211.1685	175.1472,151.1474,109.1006,83.0851,69.0695,55.0540
29	9	891	terpene	2595	C13 H20 O	+	8.17	4.19E+06	193.1587	193.1577	151.1111,149.1315,137.0953,123.0798,109.0642,69.0696
30	10	902	terpene	2-[(1E)-1-Buten-3-yn-1-yl]-1,3,3-trimethylcyclohex	C13 H18	+	8.16	2.08E+06	175.1481	175.1472	147.1160,133.1005,119.0849,105.0693,91.0538,83.0851,69.0695
31	11	141	terpene	NP-019547	C17 H26 O5	-	16.23	8.15E+05	309.1707	309.1708	101.0228,59.0129
91	12	838	terpene	3748	C13 H24 O3	+	12.33	1.18E+06	229.1798	229.1789	155.1423,99.0436,95.0851,71.0852,57.0696
123	13	1125	terpene	Pulegone	C10 H16 O	+	8.16	1.18E+07	153.1274	153.1266	125.0954,97.0643,81.0695,69.0332,55.0540
32	1	465	steroid	17-Hydroxy-17-methylestra-4,9,11-trien-3-one	C19 H24 O2	+	17.47	1.78E+05	285.1849	285.1836	131.0848,105.0693,95.0486,71.0487,59.0487,57.0332
33	1	59	quinone	2-Ethynyl-1,4-benzoquinone	C8 H4 O2	+	4.00	1.65E+06	133.0284	133.0278	105.0330,79.0175,55.0176,51.0227
34	2	539	quinone	Embelin	C17 H26 O4	+	11.79	2.17E+06	295.1904	295.1990	125.0225,101.0228,85.1009,73.0280,71.0852,57.0332
35	3	1101	quinone	2,5,6,8-Tetrahydroxy-1,4-naphthoquinone	C10 H6 O6	+	4.60	1.91E+06	223.0237	223.0229	179.0332,167.0694,153.0178,151.0382,125.0227
36	4	1102	quinone	FLAVIOLIN	C10 H6 O5	+	3.68	2.87E+06	207.0288	207.0278	179.0330,163.0382,151.0384,136.0863,109.0279
37	5	1106	quinone	lawsone	C10 H6 O3	+	6.83	1.26E+06	175.0390	175.0381	131.0484,119.0486,105.0329,103.0536,77.0383
38	1	379	flavonoid	Astilbin	C21 H22 O11	+	5.66	9.91E+05	451.1235	451.1214	451.1214
39	2	380	flavonoid	Myricitrin	C21 H20 O12	+	7.27	5.11E+06	465.1028	465.1005	465.1005
40	3	617	flavonoid	NP-000924	C16 H14 O5	+	10.78	6.45E+05	287.0914	287.0900	167.0332,139.0384,121.1007,119.0487,93.0330
41	4	747	flavonoid	(-)-Fustin	C15 H12 O6	+	5.68	1.16E+07	289.0707	289.0791	167.0330,153.0539,149.0227,137.0228,127.0384,109.0279,93.0330
42	5	756	flavonoid	Quercetin	C15 H10 O7	+	8.08	2.70E+07	303.0499	303.0485	165.0174,153.0174,137.0226,125.0229,13.0435,109.0280
43	6	758	flavonoid	Kaempferol	C15 H10 O6	+	9.78	3.09E+06	287.0550	287.0536	167.0330,151.0384,139.0383,123.0434,121.0277,111.0435,105.0329
44	7	761	flavonoid	Apigenin	C15 H10 O5	+	5.68	1.06E+07	271.0601	271.0588	153.0176,125.0227,119.0486,109.0279,93.0329
45	1	17	phenolic acids, carb	1H-Isochromene-1,3,4-trione	C9 H4 O4	+	3.69	3.78E+06	177.01823	177.0174	149.0226,133.0278,121.0279,93.0331,51.0228
46	2	35	phenolic acids, carb	Ethyl gallate	C9 H10 O5	+	5.06	3.04E+06	199.06009	199.0593	181.0488,171.0280,153.0540,125.0228,109.0280,93.0331
47	3	38	phenolic acids, carb	3,4-Dimethoxybenzoic acid	C9 H10 O4	+	8.65	4.14E+06	183.06518	183.0643	155.0695,137.0590,123.0434,93.0329,77.0382
48	4	47	phenolic acids, carb	2145	C8 H8 O2	+	9.83	3.11E+06	137.0597	137.059	109.0642,91.0538,77.0383,51.0227
49	5	52	phenolic acids, carb	Phenylglyoxylic acid	C8 H6 O3	+	10.7	3.11E+06	151.03897	151.0382	135.0434,105.0329,91.0538,77.0383,51.0226
50	6	54	phenolic acids, carb	Phthaldialdehyde	C8 H6 O2	+	6.88	4.02E+06	135.04405	135.0434	105.0330,77.0382,51.0227
51	7	57	phenolic acids, carb	Coumarandione	C8 H4 O3	+	12.91	4.39E+07	149.02332	149.0226	121.0278,93.0330
52	8	67	phenolic acids, carb	2347	C8 H14 O2	+	6.68	7.81E+06	143.10665	143.1057	125.0956,97.1007,87.0436,83.0851,69.0696,55.0540
53	9	73	phenolic acids, carb	NP-019360	C8 H12 O4	+	11.18	1.01E+07	173.08083	173.08017	113.0591,89.0229,81.0695,71.0124,67.0539,57.0696
54	10	76	phenolic acids, carb	Tetrahydrofurfuryl acrylate	C8 H12 O3	+	12.01	7.12E+06	157.08592	157.08537	113.0591,111.0435,101.0228,73.0280,57.0696
55	11	78	phenolic acids, carb	Dimedone	C8 H12 O2	+	9.52	8.22E+05	141.091	141.0905	141.0902,95.0851,81.0331,53.0383
56	12	79	phenolic acids, carb	cis-dihomoaconitic acid	C8 H10 O6	+	9.07	1.10E+06	203.05501	203.05425	139.0019,129.0540,101.0228,83.0124,67.0589,55.0540
57	13	85	phenolic acids, carb	Tyrosol	C8 H10 O2	+	10.83	2.71E+06	139.07535	139.0747	121.0277,111.0435,93.0331,77.0382,51.0227
58	14	88	phenolic acids, carb	128330	C7 H8 O4	+	3.91	3.28E+06	157.04953	157.0488	113.0228,97.0279,83.0488,67.0175
59	15	93	phenolic acids, carb	Gallic acid	C7 H6 O5	+	8.89	2.19E+06	171.02879	171.0279	153.0175,143.0332,125.0227,109.0279
60	16	95	phenolic acids, carb	Salicylic acid	C7 H6 O3	+	3.78	3.42E+06	139.03897	139.0384	121.0278,109.0278,93.0331,77.0382,51.0227
61	17	100	phenolic acids, carb	D-pinitol	C7 H14 O6	+	1.55	9.66E+06	195.08631	195.08551	141.0540,85.0281,71.0489,57.0333
62	18	101	phenolic acids, carb	n-Butyl lactate	C7 H14 O3	+	8.99	1.45E+06	147.10157	147.10094	115.0383,91.0385,73.0280,57.0696
63	19	108	phenolic acids, carb	Ethyl acetonoxalate	C7 H10 O4	+	9.16	5.48E+06	159.06518	159.0645	141.0539,127.0383,113.0591,99.0436,85.0280,71.0488
64	20	119	phenolic acids, carb	5-hydroxy-4-methoxy-5,6-dihydro-2H-pyran-2-on	C6 H8 O4	+	6.21	3.67E+07	145.04953	145.0489	127.0384,99.0436,97.0280,85.0280,69.0332,55.0176
65	21	130	phenolic acids, carb	Kojic acid	C6 H6 O4	+	4.34	8.16E+06	143.03388	143.0332	125.0227,115.0385,85.0644,69.0332,53.0020
66	22	134	phenolic acids, carb	Phloroglucinol	C6 H6 O3	+	9.53	6.67E+07	127.03897	127.0383	109.0278
67	23	165	phenolic acids, carb	Adipic acid	C6 H10 O4	+	22.57	3.72E+08	147.06518	147.0643	129.0540,111.0435,101.0592,83.0487
68	24	182	phenolic acids, carb	Monomethyl succinate	C5 H8 O4	+	4.9	5.28E+06	133.04953	133.0489	115.0384,101.0228,87.0436,73.0281,57.0332
69	25	196	phenolic acids, carb	pentane-1,2,3,4,5-pentol	C5 H12 O5	+	1.54	1.31E+06	153.07575	153.075	135.0646,117.0541,99.0436,69.0332,57.0332
70	26	275	phenolic acids, carb	C12E7	C26 H54 O8	+	16.45	6.62E+07	495.38914	495.38727	177.1113,133.0853,89.0593,85.1008
71	27	276	phenolic acids, carb	C14E6	C26 H54 O7	+	19.63	1.78E+07	479.39423	479.39.230	177.1113,133.0853,89.0593
72	28	326	phenolic acids, carb	BUTYL OLEATE	C22 H42 O2	+	21.3	9.25E+05	339.32575	339.32431	153.1266,139.1110,125.0954,115.0748,85.1007,71.0851,69.0695
73	29	328	phenolic acids, carb	MFCD00038340	C22 H36 O2	+	18.44	6.40E+06	333.2788	333.27734	111.1163,97.1007,101.0592,71.0852,67.0539,57.0696
74	30	339	phenolic acids, carb	CM2100000	C21 H40 O4	+	17.56	9.93E+06	357.29993	357.2984	115.0748,101.0748,83.0851,61.0281,57.0332
75	31	345	phenolic acids, carb	PROPYLENE GLYCOL 1-OLEATE	C21 H40 O3	+	22.93	2.67E+06	341.30502	341.30371	141.167,99.0801,85.1008,71.0852,57.0696
76	32	354	phenolic acids, carb	1-Linoleoyl glycerol	C21 H38 O4	+	15.84	1.06E+07	355.28428	355.28317	179.1785,165.1630,97.1007,57.0696
77	33	363	phenolic acids, carb	glycidyl oleate	C21 H38 O3	+	17.56	5.71E+06	339.28937	339.28799	111.0799,97.0643,85.1007,57.0696
78	34	369	phenolic acids, carb	5-[(Z)-Pentadec-8-enyl]benzene-1,3-diol	C21 H34 O2	+	18.91	6.39E+06	319.26315	319.26166	179.1057,165.0901,151.0746,137.0590,123.0435,111.1163,85.1008,71.0852,57.0696
79	35	477	phenolic acids, carb	C12E3	C18 H38 O4	+	16.38	1.85E+07	319.28428	319.28275	151.0958,133.0853,113.1320,107.0698,89.0593,85.1008
80	36	531	phenolic acids, carb	Undecyl b-D-glucopyranoside	C17 H34 O6	+	17.31	3.90E+06	335.24281	335.24149	139.0382,127.1477,113.1318,99.1163,85.1008,71.0852
81	37	542	phenolic acids, carb	HO-dPEG8-OH	C16 H34 O9	+	5.58	3.28E+07	371.22755	371.22589	221.1376,195.1222,177.1114,175.0957,133.0854,131.0698,101.0593,89.0593
82	38	671	phenolic acids, carb	Ethyl 3,4-bis(2-methoxyethoxy)benzoate	C15 H22 O6	+	8.02	4.01E+06	299.14891	299.14752	225.0748,197.0799,179.0694,165.0538,161.0589,127.0382,121.0277,109.0279,73.0281
83	39	694	phenolic acids, carb	3,5-di-tert-Butyl-4-hydroxybenzaldehyde	C15 H22 O2	+	8.7	2.95E+06	235.16925	235.16829	179.1059,161.0953,151.1109,57.0696
84	40	695	phenolic acids, carb	1,8-Cyclopentadecadiyne	C15 H22	+	11.25	6.64E+06	203.17942	203.1787	175.1473,161.1317,17.1161
85	41	723	phenolic acids, carb	1-Nonynylbenzene	C15 H20	+	7.68	1.04E+07	201.16377	201.1628	123.1162,117.0693,103.0537,71.0852,57.0696
86	42	766	phenolic acids, carb	3-(Isobutyryloxy)-2,2-dimethylpropyl 3-hydroxy-2,2	C14 H26 O5	+	10.79	2.49E+06	275.1853	275.18408	201.1112,187.1319,173.1164,101.0593,75.0437,71.0488
87	43	803	phenolic acids, carb	(2R,5R,6R)-3-[(1E,3E)-hepta-1,3-dien-1-yl]-5,6-dihy	C14 H22 O4	+	11.77	3.95E+06	255.15908	255.15794	199.0957,163.0746,135.1162,121.1006,111.0435,83.0487,71.0488,57.0696
88	44	810	phenolic acids, carb	Octylbenzene	C14 H22	+	12.15	5.88E+05	191.17942	191.1785	93.0694,79.0538,57.0696
89	45	814	phenolic acids, carb	MFCD00019210	C14 H20	+	6.32	1.22E+06	189.16377	189.1629	161.1317,147.1161,133.1005
90	46	815	phenolic acids, carb	4-(4-methoxy-2,3,6-trimethyl-phenyl)-but-3-en-2-	C14 H18 O2	+	3.7	1.05E+06	219.13795	219.13708	203.1421,149.0953,69.0332,53.0383
92	47	839	phenolic acids, carb	2-Tridecenoic acid	C13 H24 O2	+	7.96	1.30E+06	213.1849	213.1841	177.1629,97.0280,81.0695,69.0696,55.0176
93	48	859	phenolic acids, carb	5-(6-hydroxy-6-methyloctyl)-2,5-dihydrofuran-2-o	C13 H22 O3	+	7.43	2.90E+06	227.16417	227.16327	191.1421,181.1213,163.1476,153.1264,111.0435,83.0124
94	49	895	phenolic acids, carb	Ibuprofen	C13 H18 O2	+	5.85	1.96E+07	207.13795	207.13702	189.1264,161.1317,133.1005,73.0280
95	50	896	phenolic acids, carb	Heptanophenone	C13 H18 O	+	11.5	4.69E+07	191.14304	191.142	121.0642,107.0486,95.0487,77.0382,57.0696
96	51	917	phenolic acids, carb	1-Hydroxycyclohexyl Phenyl Ketone	C13 H16 O2	+	7.19	1.71E+06	205.1223	205.12157	177.0903,163.0745,159.0797,145.0641,131.0484,107.0486,77.0382
97	52	918	phenolic acids, carb	Cyclohexyl phenyl ketone	C13 H16 O	+	5.75	1.60E+07	189.12739	189.1265	161.0954,147.0798,133.0641,111.0799,107.0487,83.0851
98	53	921	phenolic acids, carb	4-Pentylphenylacetylene	C13 H16	+	11.5	2.13E+06	173.13247	173.1316	117.0693,57.0696
99	54	926	phenolic acids, carb	E-2-Benzylidenecyclohexanone	C13 H14 O	+	10.58	6.00E+05	187.11174	187.1108	159.1159,145.1004,131.0848,109.0643,103.0536,91.0538
100	55	927	phenolic acids, carb	2-Hydroxy-3-(8-hydroxy-6-methoxy-1-oxo-1H-isc	C13 H12 O7	+	4.62	5.11E+07	281.06557	281.06421	207.0642,193.0486,167.0331,165.0539,71.0124
101	56	930	phenolic acids, carb	5-[(1,3-Benzodioxol-5-yloxy)methyl]-2-furoic acid	C13 H10 O6	+	4.6	8.43E+07	263.05501	263.05374	207.0643,189.0537,163.0382,81.0330,55.0176
102	57	950	phenolic acids, carb	NP-000124	C12 H20 O4	+	9.81	3.82E+06	229.14343	229.1425	211.1321,169.1216,127.0747,85.0644
103	58	976	phenolic acids, carb	1-t-butyl-4-ethylbenzene	C12 H18	+	8.05	8.13E+05	163.14812	163.1476	135.1162,57.0696
104	59	979	phenolic acids, carb	NP-013538	C12 H16 O8	+	6.2	1.68E+07	289.09179	289.09058	181.0487,145.0489,127.0384,109.0279,97.0280,69.0332
105	60	986	phenolic acids, carb	6-Phenylhexanoic acid	C12 H16 O2	+	7.69	5.67E+06	193.1223	193.1214	147.1163,133.1006,119.0850,105.0694,91.0538,77.0383
106	61	996	phenolic acids, carb	4'-tert-Butylacetophenone	C12 H16 O	+	17.46	5.80E+05	177.12739	177.1265	133.1007,57.0696
107	62	997	phenolic acids, carb	CZ1330000	C12 H16	+	5.85	1.60E+06	161.13247	161.1319	119.0849,105.0694,91.0538,79.0539,55.0540
108	63	1001	phenolic acids, carb	Resorcinol diglycidyl ether	C12 H14 O4	+	5.63	1.99E+06	223.09648	223.09564	179.0694,149.0590,141.0540,123.0435,93.0330,77.0381,61.0394
109	64	1005	phenolic acids, carb	4-hydroxy-3-(3-methylbut-2-en-1-yl)benzoic acid	C12 H14 O3	+	7.12	2.99E+06	207.10157	207.1008	189.0900,161.0952,147.0434,69.0695,55.0540
110	65	1009	phenolic acids, carb	Butylphthalide	C12 H14 O2	+	6.82	1.78E+06	191.10665	191.1057	95.0488,57.0696
112	66	1028	phenolic acids, carb	Ethyl 2-acetylheptanoate	C11 H20 O3	+	10.84	1.01E+06	201.14852	201.1478	127.1111,57.0696
113	67	1048	phenolic acids, carb	3422	C11 H18 O	+	4.92	1.56E+06	167.14304	167.1422	121.0642,95.0487,67.0539,55.0540
114	68	1061	phenolic acids, carb	5-Pentylresorcinol	C11 H16 O2	+	9.33	3.43E+06	181.1223	181.1213	163.1109,125.0591,111.0435,57.0696
115	69	1062	phenolic acids, carb	n-Amylbenzene	C11 H16	+	8.04	1.45E+06	149.13247	149.13184	93.0695,79.0539
116	70	1067	phenolic acids, carb	2-Isopropyl-5-methylbenzoic acid	C11 H14 O2	+	10.88	2.75E+06	179.10665	179.1057	161.0953,151.1109,133.1005,109.0642
117	71	1068	phenolic acids, carb	Valerophenone	C11 H14 O	+	5.85	1.19E+06	163.11174	163.111	103.0538,81.0332,69.0332,57.0332
118	72	1069	phenolic acids, carb	6-METHYLTETRALIN	C11 H14	+	5.75	1.21E+06	147.11682	147.1161	119.0849,105.0693,91.0538,55.0540
119	73	1109	phenolic acids, carb	NP-019374	C10 H18 O4	+	16.72	5.44E+06	203.12778	203.12709	185.1163,157.1215,143.1058,101.0592,87.0436
120	74	1112	phenolic acids, carb	Queen Bee Acid	C10 H18 O3	+	5.26	1.81E+06	187.13287	187.13223	169.1214,141.0902,127.0748,113.0591,85.0280,69.0696
121	75	1117	phenolic acids, carb	(2E)-decenoic acid	C10 H18 O2	+	8.16	9.81E+05	171.13795	171.1372	87.0437,73.0281
122	76	1121	phenolic acids, carb	Ethyl 5,5-dimethyl-2,4-dioxohexanoate	C10 H16 O4	+	9.53	1.87E+07	201.11213	201.11134	145.0489,99.0436,85.0644,57.0696
124	77	1135	phenolic acids, carb	Guaifenesin	C10 H14 O4	+	4.45	1.84E+06	199.09648	199.0955	181.0850,167.0695,107.0486,95.0487,77.0382
125	78	1151	phenolic acids, carb	CY9070000	C10 H14	+	8.16	4.89E+06	135.11682	135.1162	93.0694,79.0538,57.0696
126	79	1159	phenolic acids, carb	Propylparaben	C10 H12 O3	+	9.38	5.81E+06	181.08592	181.0851	153.0537,137.0589,111.0435,93.0329,87.0437
127	80	1161	phenolic acids, carb	Butyrophenone	C10 H12 O	+	5.85	2.42E+06	149.09609	149.0954	107.0486
128	81	1165	phenolic acids, carb	3-Butenylbenzene	C10 H12	+	8.04	1.84E+07	133.10117	133.1005	105.0693,91.0538,77.0382

WS site			Compound categor	Name	formular	Ion mode	RT	Response	eoretical va	Measured value	MS/MS
1	1	5	phenylpropanoid	3,5,7-Trihydroxy-4H-chromen-4-one	C9 H6 O5	+	3.66	3.11E+06	195.0288	195.0281	167.0334,109.0280
2	2	6	phenylpropanoid	Umbelliferone	C9 H6 O3	+	12.89	1.57E+06	163.0390	163.0383	135.0434,119.0486
3	3	476	phenylpropanoid	6-t-Butyl-4-methylcoumarin	C14 H16 O2	+	11.09	9.98E+04	217.1223	217.1214	57.0696
4	4	512	phenylpropanoid	Norkhellol	C12 H8 O5	+	4.60	4.50E+06	233.0444	233.0435	215.0332,191.0332,135.0435,99.0071,57.0333
5	5	533	phenylpropanoid	NP-020400	C12 H18 O5	+	6.51	1.97E+06	243.1227	243.1216	169.0851,151.0746,137.0591,57.0332
6	6	547	phenylpropanoid	(5,7-Dihydroxy-4-methyl-2-oxo-2H-chromen-3-yl)ac	C12 H10 O6	+	4.59	3.46E+07	251.0550	251.0539	191.0329,179.0333,162.0305,135.0433,71.0124
7	7	550	phenylpropanoid	6-Methoxy-4-oxo-4H-chromene-2-carboxylic acid	C11 H8 O5	+	4.32	2.53E+06	221.0444	221.0437	193.0486,177.0537,175.0381,161.0588
8	8	575	phenylpropanoid	Scoparone	C11 H10 O4	+	7.32	1.33E+06	207.0652	207.0644	175.0381,165.0539
9	9	577	phenylpropanoid	2,4-Dihydroxy-6-pyruvoylbenzoic acid	C10 H8 O6	+	4.58	1.89E+06	225.0394	225.0385	197.0435,179.0330,163.0382,55.0176
10	10	578	phenylpropanoid	Fraxetin	C10 H8 O5	+	4.60	1.99E+07	209.0444	209.0438	194.0201,181.0486,177.0174,165.0538
11	11	605	phenylpropanoid	(E)-2-Methoxycinnamaldehyde	C10 H10 O2	+	8.42	3.39E+05	163.0754	163.0746	135.0797,131.0485,121.0642,107.0486,91.0538,77.0383,55.0176
12	1	62	steroid	Trenbolone Acetate	C20 H24 O3	-	18.26	6.28E+06	311.1653	311.1652	311.1652
13	2	259	steroid	Methandriol	C20 H32 O2	+	15.62	3.94E+05	305.2475	305.2462	121.1005,107.0850,95.0850,93.0694,57.0332,55.0540
14	1	77	terpene	NP-019547	C17 H26 O5	-	17.80	3.51E+05	309.1707	309.1704	309.1704
15	2	149	terpene	Lup-20(29)-ene-3,28-diyl (2E,2'E)bis(3-phenylacrylate)	C48 H62 O4	+	18.55	1.86E+07	703.4721	703.4717	121.1006,91.0538,69.0696,55.0539
16	3	503	terpene	3420	C13 H18 O	+	8.05	3.19E+05	191.1430	191.1412	135.0799,133.1007,109.0643,69.0332,67.0539
17	1	170	flavonoid	5,7-Dihydroxy-2-(4-hydroxyphenyl)-4-oxo-4H-chrom	C25 H26 O11	+	13.69	6.62E+05	503.1548	503.1550	133.0852,111.0799,107.0487,101.0592,97.0644,57.0696
18	2	441	flavonoid	Taxifolin	C15 H12 O7	+	7.14	5.23E+05	305.0656	305.0642	153.0175,151.0382,149.0225,139.0383,135.0435,127.0383,109.0278
19	3	443	flavonoid	Quercetin	C15 H10 O7	+	8.04	1.01E+07	303.0499	303.0485	165.0173,153.0175,123.0434,109.0278
20	1	469	quinone	DK3970000	C14 H20 O2	+	5.57	1.74E+05	221.1536	221.1528	83.0851,57.0696
21	1	13	phenolic acids,	(E)-3-cyclohexylacrylic acid	C9 H14 O2	+	5.15	5.23E+05	155.1067	155.1060	137.0954,127.0748,109.1007,83.0852
22	2	20	phenolic acids,	D-diacetyltartaric anhydride	C8 H8 O7	+	13.87	3.64E+05	217.0343	217.0335	129.0540,97.0278,85.0280
23	3	21	phenolic acids,	Isovanillic acid	C8 H8 O4	+	1.52	2.08E+06	169.0495	169.0487	151.0383,141.0539,137.0226,123.0434
24	4	26	phenolic acids,	Coumarandione	C8 H4 O3	+	12.89	1.23E+07	149.0233	149.0228	121.028
25	5	29	phenolic acids,	PEG-4	C8 H18 O5	+	16.35	1.75E+06	195.1227	195.1220	89.0593,87.0437,75.0438,57.0333
26	6	35	phenolic acids,	(2Z)-2-(1,4-Dioxan-2-yl)-2-butenedioic acid	C8 H10 O6	+	1.52	4.10E+05	189.0758	203.0542	129.0539,115.0384,87.0436,71.0487,57.0333
27	7	36	phenolic acids,	5-Ethoxy-2-furoic acid	C7 H8 O4	+	4.49	3.24E+06	157.0495	157.0488	139.0384,129.0177,111.0436,97.0644,83.0123
28	8	39	phenolic acids,	2-Methoxyresorcinol	C7 H8 O3	+	1.52	1.72E+07	141.0546	141.0540	123.0435,109.0279
29	9	44	phenolic acids,	D-pinitol	C7 H14 O6	+	1.52	2.72E+07	195.0863	195.0859	177.0752,159.0646,145.0490,141.0541,127.0385,85.0281,53.0384
30	10	49	phenolic acids,	Monomethyl adipate	C7 H12 O4	+	13.71	1.89E+06	161.0808	161.0801	143.0696,129.0540,115.0749,111.0436,101.0593,83.0488
31	11	57	phenolic acids,	Ethyl acetonoxalate	C7 H10 O4	+	1.52	1.85E+07	159.0652	159.0646	115.0384,113.0228,87.0437,85.0280,57.0333
32	12	71	phenolic acids,	5-hydroxy-4-methoxy-5,6-dihydro-2H-pyran-2-one	C6 H8 O4	+	3.10	7.28E+07	145.0495	145.0489	127.0384,83.0125,55.0176
33	13	86	phenolic acids,	Kojic acid	C6 H6 O4	+	4.33	1.87E+06	143.0339	143.0332	125.0227,115.0384,101.0228,85.0280,57.0332
34	14	87	phenolic acids,	5-Hydroxymethyl-2-furaldehyde	C6 H6 O3	+	1.52	3.42E+07	127.0390	127.0384	109.0179,99.0436,97.0280,81.0332,69.0332,55.0176
35	15	126	phenolic acids,	2,2-Bis(hydroxymethyl)propionic acid	C5 H10 O4	+	1.51	2.69E+06	135.0652	135.0645	117.0541,75.0437,57.0332
36	16	174	phenolic acids,	C12E6	C24 H50 O7	+	16.38	6.25E+07	451.3629	451.3610	177.1113,133.0854,89.0593,85.1008
37	17	196	phenolic acids,	Laureth-5	C22 H46 O6	+	16.38	6.17E+07	407.3367	407.3350	133.0853,89.0593,85.1008
38	18	198	phenolic acids,	undecaethylene glycol	C22 H46 O12	+	6.52	3.81E+07	503.3062	503.3047	177.1113,133.0853,89.0593
39	19	212	phenolic acids,	Methyl 15-phenylpentadecanoate	C22 H36 O2	+	18.34	8.61E+05	333.2788	333.2773	93.0694,79.0538,75.0436,57.0332
40	20	218	phenolic acids,	Ethyl îµ-oxo-9-phenanthrenehexanoate	C22 H22 O3	+	18.63	2.46E+05	335.1642	335.1653	89.0593
41	21	231	phenolic acids,	5-[(Z)-Pentadec-8-enyl]benzene-1,3-diol	C21 H34 O2	+	18.77	6.48E+05	319.2632	319.2618	235.1684,179.1058,165.0904,137.0591,123.0435,111.1163,71.0852
42	22	241	phenolic acids,	3,6,9,12,15,18-Hexaoxahexacosan-1-ol	C20 H42 O7	+	22.33	2.63E+07	395.3003	395.3009	71.0851,57.0696
43	23	243	phenolic acids,	C12E4	C20 H42 O5	+	16.32	6.47E+07	363.3105	363.3188	195.1218,177.1114,133.0854,89.0593,85.1008
44	24	275	phenolic acids,	6132055	C19 H36 O3	+	19.22	1.03E+07	313.2737	313.2723	97.1008,81.0695,71.0852,57.0696
45	25	297	phenolic acids,	Nonaethylene Glycol	C18 H38 O10	+	5.90	4.86E+07	415.2538	415.2522	177.1113,133.0854,89.0593,87.0437
46	26	300	phenolic acids,	(5R)-5-(1-Hydroxytridecyl)-alpha-D-xylopyranose	C18 H36 O6	+	17.23	7.21E+06	349.2585	349.2563	181.0703,179.0547,75.0438
47	27	302	phenolic acids,	(12Z)-9,10,11-trihydroxyoctadec-12-enoic acid	C18 H34 O5	+	17.25	6.10E+07	331.2479	331.2463	185.1164,101.0592,75.0427,71.0852,57.0696
48	28	341	phenolic acids,	13(S)-HOTrE	C18 H30 O3	+	13.60	1.35E+06	295.2268	295.2255	179.1421,161.1317,135.1162,121.1006,107.0850,81.0695,71.0850
49	29	342	phenolic acids,	1913256	C18 H30 O	+	14.41	1.35E+06	263.2369	263.2357	57.0697
50	30	348	phenolic acids,	Methyl (2S,3R,4S)-2-(beta-D-glucopyranosyloxy)-4-(2	C18 H26 O11	+	6.52	7.55E+06	419.1548	419.1533	131.0333,113.0228,97.0280,85.0281,73.0281,71.0124,57.0333
51	31	365	phenolic acids,	PEG n8	C16 H34 O9	+	5.58	9.62E+07	371.2276	371.2258	177.1113,133.0853,101.0593,89.0593
52	32	371	phenolic acids,	Gemcabene	C16 H30 O5	+	12.21	3.36E+06	303.2166	303.2153	101.0593,97.1008,87.0436,83.0487,55.0540
53	33	407	phenolic acids,	6-(7-methyloctyl)-1H,3H,4H,6H-furo[3,4-c]furan-1-on	C15 H24 O3	+	11.54	9.36E+04	253.1798	253.1788	127.0384,99.0436,97.0280,85.1007,71.0852,57.0696
54	34	422	phenolic acids,	3,5-di-tert-Butyl-4-hydroxybenzaldehyde	C15 H22 O2	+	12.39	2.97E+06	235.1693	235.1683	179.1058,161.0952,57.0696
55	35	431	phenolic acids,	Atractylon	C15 H20 O	+	12.63	1.52E+05	217.1587	217.1579	135.0798,109.1007,93.0695,95.0487,81.0696,55.0540
56	36	451	phenolic acids,	MFCD00054545	C14 H26 O5	+	10.75	2.58E+06	275.1853	275.1841	97.0644,89.0593,81.0695,71.0488,59.0488,55.0540
57	37	470	phenolic acids,	(2S,4R,5S,6S,7R)-5,6,12,14-tetrahydroxy-4-(hydroxyme	C14 H16 O9	+	4.59	1.43E+08	329.0867	329.0852	211.0228,209.0435,195.0280,103.0386,85.0281,69.0332
58	38	478	phenolic acids,	4-Methylbenzophenone	C14 H12 O	+	11.64	2.06E+07	197.0961	197.0952	19.0486,105.0330,91.0538,77.0382
59	39	484	phenolic acids,	1,4-Dioxacyclopentadecan-5-one	C13 H24 O3	+	12.34	1.58E+06	229.1798	229.1789	155.1423,99.0436,71.0852,57.0696
60	40	506	phenolic acids,	ionene	C13 H18	+	10.76	3.35E+05	175.1481	175.1475	133.1007,119.0850,105.0694,91.0538,83.0851
61	41	509	phenolic acids,	3a,8b-Dihydroxy-2-(hydroxymethyl)-4-oxo-4,8b-dihy	C13 H10 O7	+	3.66	5.71E+06	279.0499	279.0486	139.0384,121.0278,119.0485,107.0486,95.0487,85.0281
62	42	511	phenolic acids,	Benzophenone	C13 H10 O	+	10.86	1.13E+06	183.0804	183.0798	105.0330,77.0381
63	43	536	phenolic acids,	NP-021018	C12 H18 O4	+	15.25	1.06E+06	227.1278	227.1269	1167.0696,139.0747,121.1006,95.0487,83.0851,69.0696
64	44	556	phenolic acids,	Methyl 10-oxodecanoate	C11 H20 O3	+	10.82	1.95E+06	201.1485	201.1476	159.1371,141.1265,127.1111,125.0952,101.0592
65	45	560	phenolic acids,	3,3-Dimethyl-1,5-dioxaspiro(5.5)undecan-9-one	C11 H18 O3	+	13.56	1.45E+05	199.1329	199.1321	125.0591,111.0797,95.0487,85.0643,57.0332
66	46	568	phenolic acids,	4-Ethoxy ethylbenzoate	C11 H14 O3	+	11.47	1.18E+06	195.1016	195.1008	167.0695,149.0591,121.0279,113.0592
67	47	570	phenolic acids,	4-Isobutylbenzoic acid	C11 H14 O2	+	10.57	3.13E+05	179.1067	179.1058	133.1004,95.0487
68	48	585	phenolic acids,	PEG n5	C10 H22 O6	+	4.29	6.03E+06	239.1489	239.1489	221.1385,177.1113,151.0959,133.0853,89.0593
69	49	589	phenolic acids,	NP-019374	C10 H18 O4	+	16.67	6.24E+06	203.1278	203.1272	185.1163,157.1216,125.0954,111.0799,101.0593,83.0488
